# Synthesis, characterization and study of electrochemical applicability of novel asymmetrically substituted 1,3-dialkyl-1,2,3-benzotriazolium salts for supercapacitor fabrication†

**DOI:** 10.1039/d3ra01958f

**Published:** 2023-05-15

**Authors:** Anjitha Satheesh, Punnakkal Navaneeth, Punathil Vasu Suneesh, Sarathchandran C, Elango Kandasamy

**Affiliations:** a Department of Sciences, Amrita School of Physical Sciences Coimbatore, Amrita Vishwa Vidyapeetham 641112 India k_elango@cb.amrita.edu; b Amrita Biosensor Research Lab, Amrita School of Physical Sciences Coimbatore, Amrita Vishwa Vidyapeetham 641112 India; c Department of Sciences, Amrita School of Engineering, Amrita Vishwa Vidyapeetham Chennai India

## Abstract

Here we report the successful synthesis, fabrication, and testing of novel asymmetrically substituted 1,3-dialkyl-1,2,3-benzotriazolium-based ionic liquids. Their applicability in energy storage is tested as gel polymer electrolytes (ILGPE) immobilized in poly(vinylidene fluoride-*co*-hexa-fluoropropylene) (PVDF-HFP) copolymer as a solid-state electrolyte in electric double layer capacitors (EDLC). Asymmetrically substituted 1,3-dialkyl-1,2,3-benzotriazolium salts of tetrafluoroborates (BF_4_^−^) and hexafluorophosphates (PF_6_^−^) are synthesized by anion exchange metathesis reaction using 1,3-dialkyl-1,2,3-benzotriazolium bromide salts. *N*-Alkylation followed by quaternization reaction results in dialkyl substitution on 1,2,3-benzotriazole. The synthesized ionic liquids were characterized with ^1^H-NMR, ^13^C-NMR, and FTIR spectroscopy. Their electrochemical and thermal properties were studied using cyclic voltammetry, impedance spectroscopy, thermogravimetric analysis, and differential scanning calorimetry. The 4.0 V potential windows obtained for asymmetrically substituted 1,3-dialkyl-1,2,3-benzotriazolium salts of BF_4_^−^ and PF_6_^−^ are promising electrolytes for energy storage. ILGPE tested with symmetrical EDLC with a wide operating window from 0–6.0 V gave an effective specific capacitance of 8.85 F g^−1^ at a lower scan rate of 2 mV s^−1^, the energy density of 2.9 μW h and 11.2 mW g^−1^ power density. The fabricated supercapacitor was employed for lighting red LED (2 V, 20 mA).

## Abstract 2

Here we report the successful synthesis, fabrication, and testing of novel asymmetrically substituted 1,3-dialkyl-1,2,3-benzotriazolium-based ionic liquids.

## Graphical abstract

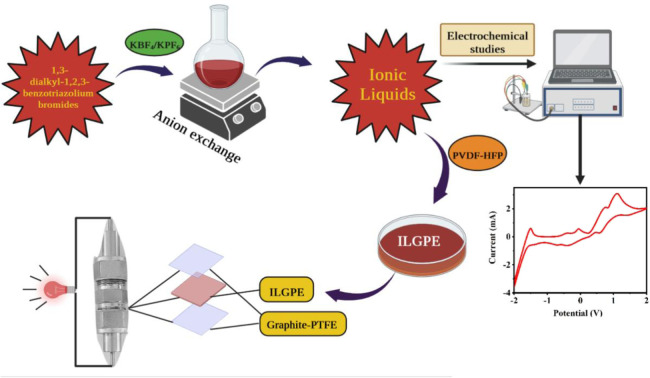

## 1. Introduction

Since their introduction, ionic liquids (ILs) have been investigated widely due to their leading-edge impact in generating promising technologies. This is evident by the exponential increase in recently published research articles.^1–8^ To resolve society's significant issues, ILs as potential candidates are emerging as an efficient, clean, and eco-friendly alternative resource of volatile organic solvents on account of their distinctive physical, chemical, thermal, and chemical–biological properties.^4^ Following their characteristic negligible vapor pressure, they have additional attractive features, like a highly ionized environment, a more comprehensive liquidus range, good thermal/electrochemical stability, and their affinity/solubility towards a vast range of synthetic and natural solvents. ILs stand out as a potential alternative due to extraordinary safety advantages over conventional organic electrolytes.^9–12^ Unlike inorganic salts, ILs cause the shielding of intermolecular force owing to their cation/anion bulk asymmetry. This, in turn, prevents slow-energy crystalline state ionic aggregation, lowering the temperature corresponding to their initial point of liquidus. Their van der Waals interaction, Lewis acidity/basicity, and other task-specific functionalities between cation and anion *via* multiple coupling can tune their physicochemical properties. Due to their unique electrochemical stability window, ILs effectively widen the operating voltage over 3.0 V.^13^ They can effectively be used as electrolytes for critical energy storage applications like batteries and supercapacitors.^14–17^ In spite of their different energy storage mechanisms, they have extended requirements for their electrolytes. Supercapacitors, by separating charges, store energy, while in batteries, electrochemical reactions are performed at electrodes.^18,19^ ILs are able to arrange them on the porous electrodes and are able to transport the redox-active species between the electrodes of supercapacitors.^20–24^ Among the various class of ILs, the potential applications of ILs, which are nitrogen-rich, have swiftly increased over prevailing energy storage materials, mainly because of their low hydrogen and carbon content, resulting in good oxygen balance.^25^

ILs with imidazolium cations are widely studied in accordance with their relatively high ionic conductivities, low melting points, and low viscosities.^21,26–30^ Consequently, they can be potentially applied as an electrolyte in supercapacitors with no additional solvent. 1-Ethyl-3-methylimidazolium bis(trifluoromethanesulfonyl)imide with pullulan-based PP-AC electrodes have shown a potential window up to 1.4 V.^31^ Zhang *et al.* (2005) reported the synthesis as well as electrochemical properties of substituted triazolium-based ILs and reported a potential window of 3 V.^32–34^ Nowell *et al.* (2018) investigated the usage of a eutectic mixture of ILs based on imidazolium and pyrrolidinium and reported a working temperature range between −70 and +80 °C with a potential window of 3.5 V.^35^ However, the poor thermal stability and low potential window of electrolytes restrict their usage in low temperatures (below 20 °C) and high temperatures (above 100 °C).

Benzotriazolium cations can form ionic liquids comparable to triazolium cations, even though relatively very few benzotriazolium salts have been published and are almost exclusively halides.^36,37^ For *N*-alkylation of benzotriazoles, different reagents and bases are used, such as sodium ethoxide, potassium hydroxide, potassium *t*-butoxide, and KF/Al_2_O_3_, by using benzotriazole as a base in surplus amounts, NCS/PPh_3_, Pd(PPh_3_), Pd/Cu(ii)/base, Cu(OAc)_2_/NaH and sodium hydroxide (in ionic liquid).^38–41^ The majority of these methods have several downsides, such as their lack of simplicity, usage of a toxic solvent, the need for catalysts, need for as base, lower yields of the products, or a large amount of waste from which the solvent cannot be recovered. Their low regioselectivity in *N*-alkylation of benzotriazole and long reaction time is still observed. Apart from these significant concerns, the derivatives of *N*^1^-alkyl benzotriazole are more significant than its *N*^2^-alkyls analogs in different aspects. To the best of our knowledge, for the highly regioselective synthesis of *N*^1^-isomers, only a few endeavors have been reported. Our ongoing interest is to synthesize a few asymmetrically substituted dialkyls of benzotriazolium salts with two different anions, such as tetrafluoroborates (BF_4_^−^) and hexafluorophosphates (PF_6_^−^), to study their electrochemical and thermal responses for fabricating a prototype with ionic liquid gel polymer electrolyte (ILGPE) for their practical application on energy storage devices.

## 2. Materials and methods

Chemicals such as 1,2,3-benzotriazole, tetrahydrofuran, 1,8-diazabicyclo[5.4.0]undec-7-ene (DBU), alkyl bromides, acetonitrile were purchased from AVERA, India. Potassium hexafluorophosphate, potassium tetrafluoroborate, and poly(vinylidene fluoride-*co*-hexa-fluoro-propylene) were procured from Sigma-Aldrich. All chemicals were used without further purification.

^1^H NMR and ^13^C NMR spectra are recorded on a Bruker AVANCE 400 FT NMR instrument in CDCl_3_. FTIR spectra are recorded on a Bruker Vector 22 FT-IR spectrometer in ATR mode operating at 400–4000 cm^−1^. Cyclic voltammetry, electrochemical impedance spectroscopic, and galvanostatic charge–discharge studies were conducted using an electrochemical analyzer (CHI608D electrochemical analyzer, CH Instruments, TX, USA). Thermogravimetric (TG) analysis is recorded in SDT Q600 V20.9 Build 20 at a heating rate of 10.00 °C min^−1^ to 610 °C and purged with nitrogen gas. Differential scanning calorimetry (DSC) was recorded on DSC Q20 V24.11 Build 124 with a cooling rate of 10.00 °C min^−1^ to 310 °C and purged with nitrogen gas.

*N*-Alkylation of 1,2,3-benzotriazole is done, as reported earlier.^42^ After the completion of the reaction, the remaining reaction mixture contains 1 and 2 isomers of alkylated benzotriazole. The isomers were separated with column chromatography, and volatiles was removed using a rotary evaporator. Representations of ^1^H and ^13^C NMR are given. All ^1^H and ^13^C NMR spectra data are available in ESI (S1).†

### 2.1 Synthesis of 1,3-dialkyl-1,2,3-benzotriazolium bromides

1-Alkyl-1,2,3-benzotriazole and alkyl bromide (1 : 1) were kept in an autoclave at 80 °C for 24 h. Reaction completion was ensured with thin-layer chromatography (TLC). The reaction mixture was then washed thoroughly and repeatedly with petroleum ether. The volatiles was removed using a rotary evaporator and dried in a vacuum.

### 2.2 Anion exchange

**Scheme 1:**
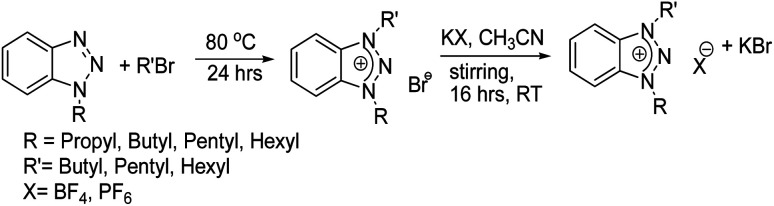
Synthesis of asymmetrically substitutes 1,3-dialkyl-1,2,3-benzotriazolium salts.

Potassium hexafluorophosphate and potassium tetrafluoroborate were used for anion exchange. 1,3-Dialkyl-1,2,3-benzotriazolium bromide was dissolved in 10 mL acetonitrile, followed by the addition of anion exchange reactant (in 1 : 1.2 ratio), followed by stirring for about 16 h (Scheme 1). After completion of the reaction, the product was removed from KBr salt through gravity filtration. The volatiles were removed using a rotary evaporator and dried in a vacuum.

### 2.3 Preparation of gel polymer electrolyte

1-Butyl-3-pentylbenzotriazolium tetrafluoroborate (1but3pentBTBF_4_) was used for fabrication. 1but3pentBTBF_4_ has a relatively better potential window of 4.0 V and offers higher ionic conductance (1.02 × 10^−2^ S) at room temperature and appreciable thermal and electrochemical stabilities. The “solution-cast” method was adopted for the preparation of ionic liquid incorporated gel polymer electrolyte (ILGPE). The polymer host PVDF-HFP was dissolved separately in acetonitrile. 1but3pentBTBF_4_ dissolved in acetonitrile was mixed with PVDF-HFP/acetonitrile solution and later then magnetically stirred for ∼12 h. A weight ratio of 4 : 1 for the ionic liquid to polymer was taken.^43^ Once a viscous solution is formed, it is then cast over on glass Petri dishes, and acetonitrile is allowed to evaporate slowly. Ultimately, a free-standing gel electrolyte of thickness ∼200 to 300 μm was obtained.

### 2.4 Preparation of electrodes

For fabricating and testing the ILGPE with 2 electrode system, graphite-polytetrafluoroethylene electrodes were fabricated as follows. A mixture of graphite powder and aqueous polytetrafluoroethylene (PTFE) solution (60 wt%) was homogenized in a mortar and pestle by adding a few drops of ethanol. The resulting dough-like mass containing ten wt% PTFE was rolled by a twin roller and was hot-pressured under a 10-ton cm^−2^. The obtained sheet was dried at 80 °C for 12 h in a hot air oven and cut into small circular plates of area 1.1304 cm^2^ to fabricate the supercapacitor using Swagelok cell of 14 mm diameter as the current collector and 1but3pentBTBF_4_ incorporated gel polymer electrolyte was used as the separator.

### 2.5 Fabrication and testing of symmetrical EDLC using ILGPE

The material was evaluated for its capacitive behavior by two electrode configurations using a Swagelok cell of 14 mm diameter supercapacitor applications. A two-electrode system was fabricated with a 11 mm diameter ILGPE sandwiched in the middle of two graphite-PTFE sheets of 11 mm diameter and tightly sealed inside the Swagelok cell. The performance tests of symmetrical EDLC cell was evaluated using electrochemical cyclic voltammetry, electrochemical impedance spectroscopy, and galvanostatic charge–discharge studies.

## 3. Results and discussions

**Fig. 1:**
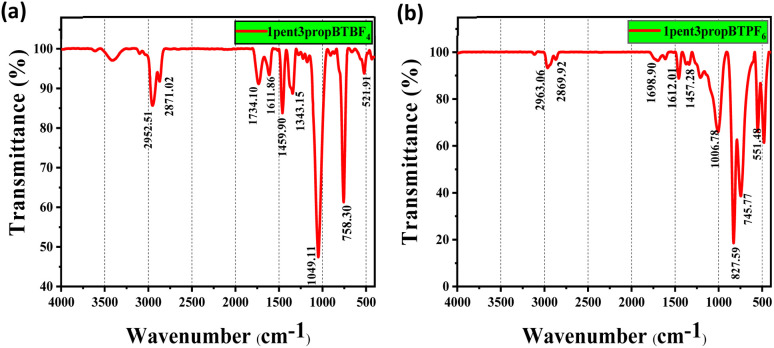
FTIR spectra of asymmetrically substituted 1,3-dialkyl-1,2,3-benzotriazolium salts, (a) 1pent3propBTBF_4_ and (b) 1pent3propBTPF_6_.

Characterization of the synthesized ILs was carried out using ^1^H-NMR, ^13^C-NMR, and FTIR spectroscopy, and the results obtained are given in ESI Sections S2 and S3.†^1^H and ^13^C NMR confirm the structure of two isomers of *N*-alkyl-1,2,3-benzotriazole. Chemical shifts are tabulated in Table S1.† For 1-isomer, four aromatic protons resonate at four different *δ* values. In the case of 2-isomer, there are only two aromatic proton signals, H^a^ = H^b^ and H^c^ = H^d^. The ^1^H NMR values suggest that the 2-isomer is symmetrical and the 1-isomer is not. Since the 2-isomer is not reactive, the 1-isomer is taken for ionic liquid synthesis. The structure of asymmetrically substituted 1,3-dialkyl-1,2,3-benzotriazolium salts containing Br^−^, BF_4_^−^, PF_6_^−^ is confirmed by ^1^H and ^13^C NMR. There are two signals for aromatic protons and three for C in an aromatic ring. The observation of chemical shift of aromatic protons of BF_4_^−^ and PF_6_^−^ containing ILs from Br^−^ in the case of 1but3propBTBr, the H^a^ proton resonates at 8.69 ppm further in case of BF_4_^−^ and PF_6_^−^ resonates at 8.41 and 8.06 ppm respectively, suggests that the anion exchange was successful (Table S1† entry 9 to 11). The presence of BF_4_^−^ and PF_6_^−^ in the ionic liquid is confirmed by FT-IR spectra (Fig. 1a and b).

The absorption peaks due to B–F and P–F bonds appear at 1049.11 and 827.99 cm^−1^ respectively. Absorption peak around 3500 cm^−1^ observed for BF_4_^−^ suggest that they are more hydrophilic than the PF_6_^−^, which is in accordance with the reported studies on hydrophilicity of anions in ionic liquids.^44–46^ Rest of the IR graphs are given in ESI Section S4.†

### 3.1 Thermal stability

**Fig. 2:**
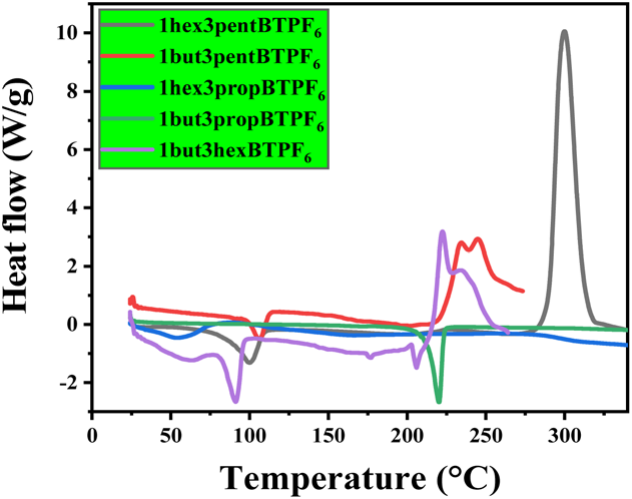
DSC graph of asymmetrically substituted 1,3-dialkyl-1,2,3-benzotriazolium salts.

**Fig. 3:**
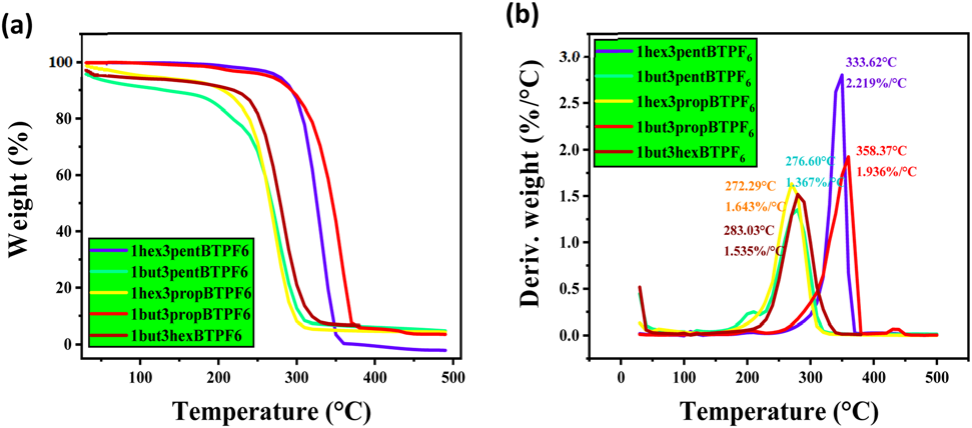
TGA graphs of asymmetrically substituted 1,3-dialkyl-1,2,3-benzotriazolium salts, (a) weight *vs.* temperature and (b) derivative weight *vs.* temperature.

Thermal stabilities of the solid samples were studied using thermogravimetric (TG) analysis, and melting points of the salts were evaluated using DSC. The salts are showing a good thermal stability up to 200 °C (Fig. 2 and 3). This shows their maximum temperature range which they can withstand. And their fraction of volatiles also seems to be less. The salts seems to have no glass transition temperature (*T*_g_). Their melting points are listed in Table 1.

**Table 1:** Thermal stability and melting points of asymmetrically substituted 1,3-dialkyl-1,2,3-benzotriazolium salts^a^

Compound	Melting point (°C)	Thermal stability (°C)
1but3pentBTPF_6_	74	200
1hex3propBTPF_6_	65	250
1but3propBTPF_6_	85	300
1but3hexBTPF_6_	70	250
1hex3pentBTPF_6_	75	300

a 1but3propBTPF_6_ and 1hex3pentBTPF_6_ salts have thermal stability up to 300 °C. These salts shows excellent thermal stability.

### 3.2 Electrochemical analysis

**Fig. 4:**
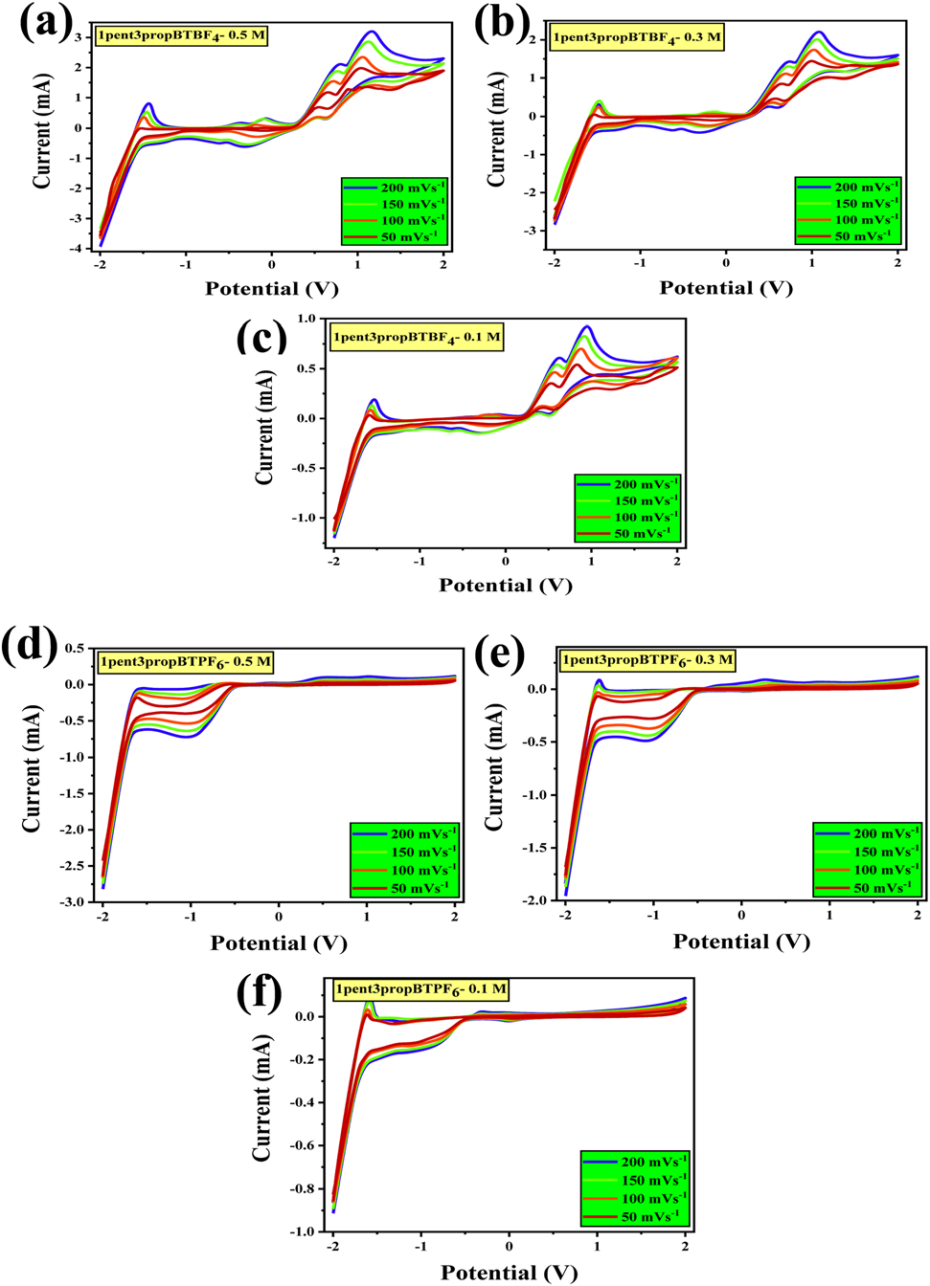
Cyclic voltammograms of asymmetrically substituted 1,3-dialkyl-1,2,3-benzotriazolium salts with 0.5, 0.3, and 0.1 M concentrations in acetonitrile, recorded at different scan rates, on Pt disc electrodes as working electrode and Pt wire as counter and reference electrodes (a) 1pent3propBTBF_4_-0.5 M, (b) 1pent3propBTBF_4_-0.3 M, (c) 1pent3propBTBF_4_-0.1 M, (d) 1pent3propBTPF_6_-0.5 M, (e) 1pent3propBTPF_6_-0.3 M, (f) 1pent3propBTPF_6_-0.1 M.

In order to evaluate the electrochemical properties, cyclic voltammograms were recorded with a three-electrode system consists of platinum (Pt, 2 mm dia) disc is a working electrode; Pt wire is both counter and reference electrodes. CV studies were conducted using 3 various concentrations 0.5, 0.3, and 0.1 M at 5 different scan rates 200, 150, 100, 50, and 20 mV s^−1^ (Fig. 4). As the concentration varies from 0.5 to 0.1, there is a decrease in the maximum anodic and cathodic points, or it can be said otherwise that the concentration does play an important role in the electrochemical responses. The stability and interaction of cation and anion influence the electrochemical window. Their interaction could be hydrophilic, ion–ion interaction, or hydrogen bonding.^47^ The alkyl side chains of the cation affect the electrochemical potential window, indicating the change in potential windows listed in Table 2.

**Table 2:** The potential window for asymmetrically substituted 1,3-dialkyl-1,2,3-benzotriazolium salts from CV studies^a^

Sl no.	Compound	Potential window (V)
1	1but3propBTBF_4_	2.8
2	1but3propBTPF_6_	2.8
3	1hex3propBTBF_4_	4
4	1hex3propBTPF_6_	4
5	1but3hexBTBF_4_	2.8
6	1but3hexBTPF_6_	4
7	1hex3pentBTBF_4_	2.8
8	1hex3pentBTPF_6_	2.8
9	1but3pentBTBF_4_	4
10	1but3pentBTPF_6_	4
11	1pent3propBTBF_4_	4
12	1pent3propBTPF_6_	4

a Out of the 12 different combinations of substituted salts 1hex3propBTBF_4_, 1hex3propBTPF_6_, 1but3hexBTPF_6_, 1but3pentBTBF_4_, 1but3pentBTPF_6_, 1pent3propBTBF_4_, and 1pent3propBTPF_6_ are having a wide potential window up to 4.0 V even with such lower concentrations.

However, as the chains are asymmetrical, we cannot predict the trend. The hydrophobicity of cation can also affect the window, which is influenced by the alkyl substitutions. These ionic liquids show redox behavior.

In systems with BF_4_^−^ anions, the oxidation is due to the oxidation of BF_4_^−^ to boron trifluoride (BF_3_), which later gets reduced to BF_4_. ILs with PF_6_^−^ get oxidized to PF_5_ and later get reduced back. Furthermore, the reduction of benzotriazolium cation seems to form a radical like imidazolium, which reacts through radical–radical coupling and disproportionation. It is then stabilized with the lone pair of nitrogen orbitals in the aromatic ring.^48^ The 1,3-dialkyl-1,2,3-benzotriazoliumtetrafluoroborates showed a better capacitance towards positive potentials, whereas the 1,3-dialkyl-1,2,3-benzotriazolium hexafluorophosphates were showing better capacitance towards negative potentials. All CVs are given in the ESI Section S5.†

**Fig. 5:**
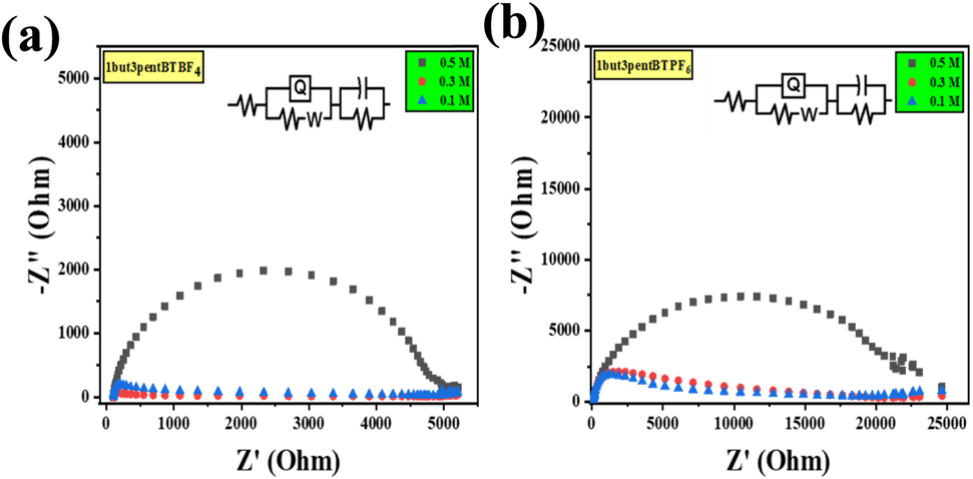
EIS graphs with different concentration 0.5, 0.3, and 0.1 M, (a) 1but3pentBTBF_4_ and (b) 1but3pentBTPF_6_.

To know the resistance to alter current electrochemical impedance spectroscopy (EIS) was taken. Through EIS, we measured and compared the charge transfer resistance of the material with three different concentrations, such as 0.5, 0.3, and 0.1 M concentrations. After fitting a suitable circuit system [R(Q(RW))(CR)], we found the resistance offered by the system. The Nyquist plots generated from EIS studies (Fig. 5) show a semicircular region in the low-frequency region. This is indicative of bulk electrolyte resistance due to ionic liquid and charge transfer or polarization resistance^49–51^ (equivalent circuit values for different liquids are given in Table S2†). We calculated the conductance of every ionic liquid from resistance, which is given in ESI (Table S2†). As concentration decreases, the system offers more resistance and hence shows a decrease in conductance. Systems like 1but3propBTBF_4_, 1but3propBTPF_6_, 1but3pentBTBF_4_, and 1but3pentBTPF_6_ shows better conductance compared to others and 1but3pentBTBF_4_, and 1but3pentBTPF_6_ is having a wider potential window of 4.0 V.

Combining all the studies, we choose 1but3pentBTBF_4_, which has a wider potential window of 4.0 V and better ionic conductance of 1.028 × 10^−2^ S and because of its smaller size of anion compared to PF_6_^−^ as smaller size of anion gives better capacitance. To analyze and illustrate the real-time working potential of these asymmetrically substituted 1,3-dialkyl-1,2,3-benzotriazolium salts, 1but3pentBTBF_4_ was chosen to conduct further studies. The cyclic stability of the salt was tested using CV, and we could not see any difference in the cyclic recoverability. Solvent evaporation after a long time made a hindrance to go further with the cycles (Fig. S104†).

**Fig. 6:**
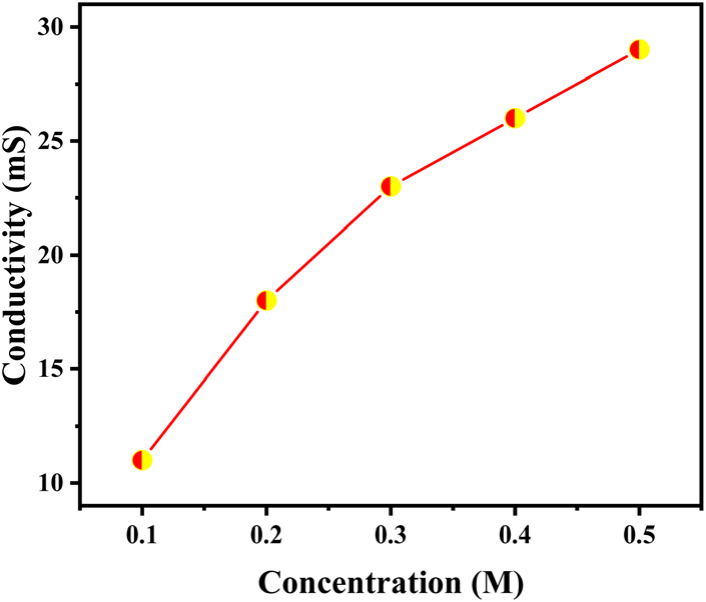
Conductivities of 1but3pentBTBF_4_ recorded with different concentrations ranging from 0.1–0.5 M in acetonitrile.

Conductivity of the ionic liquid at varying concentrations ranging from 0.1 to 0.5 M were figured out using conductivity systronics conductivity meter 304 at 298 K (Fig. 6). Conductivity shows increasing with increasing concentration due to increase in effective ion concentration.

### 3.3 Fabrication

#### 3.3.1 Characteristics of gel polymer electrolyte

**Fig. 7:**
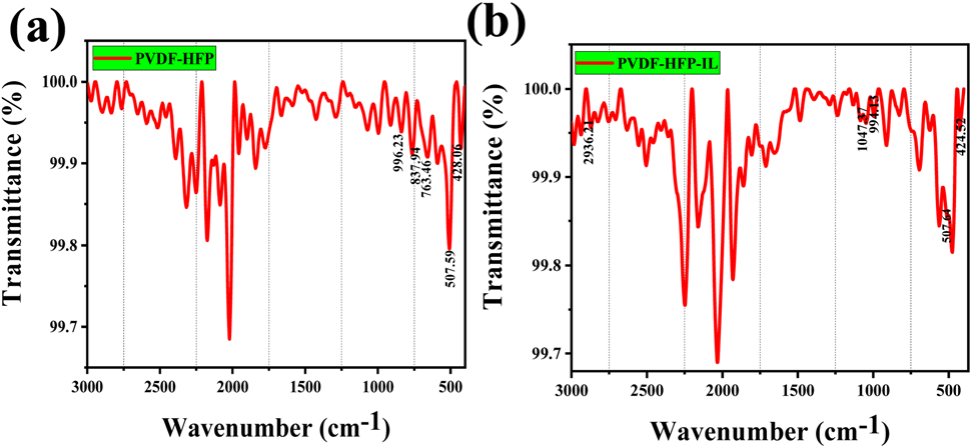
FTIR spectra of (a) PVDF-HFP film and (b) PVDF-HFP with ionic liquid film (PVDF-HFP-IL).

The FTIR spectra of poly(vinylidene fluoride-*co*-hexa-fluoro-propylene) PVDF-HFP films shows prominent peaks at 428.06, 507.59, 763.46, 837.94, 996.23 cm^−1^ (Fig. 7a). The peaks at 507.59 cm^−1^ and 996.23 cm^−1^ are assigned to nonpolar *trans*–*gauche*–*trans*–*gauche* conformation of the semicrystalline PVDF-HFP.

**Fig. 8:**
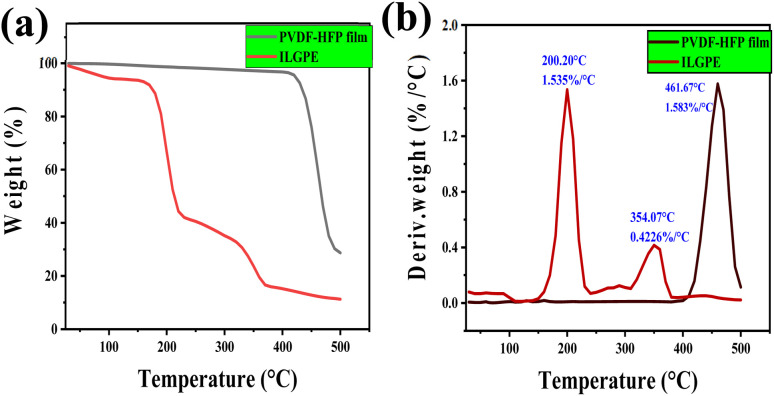
TGA spectra of (a) PVDF-HFP film and (b) PVDF-HFP with ionic liquid film (PVDF-HFP-IL).

Fig. 8 depicts the TGA results of PVDF-HFP film has a stability of up to 400 °C, and when IL is incorporated into the film, the stability is reduced to ∼200 °C. This may be due to the highly hydrophilic nature of the ILGPE. Maximum weight loss is happening around 200 °C.

#### 3.3.2 Electrochemical analysis of the fabricated device

**Fig. 9:**
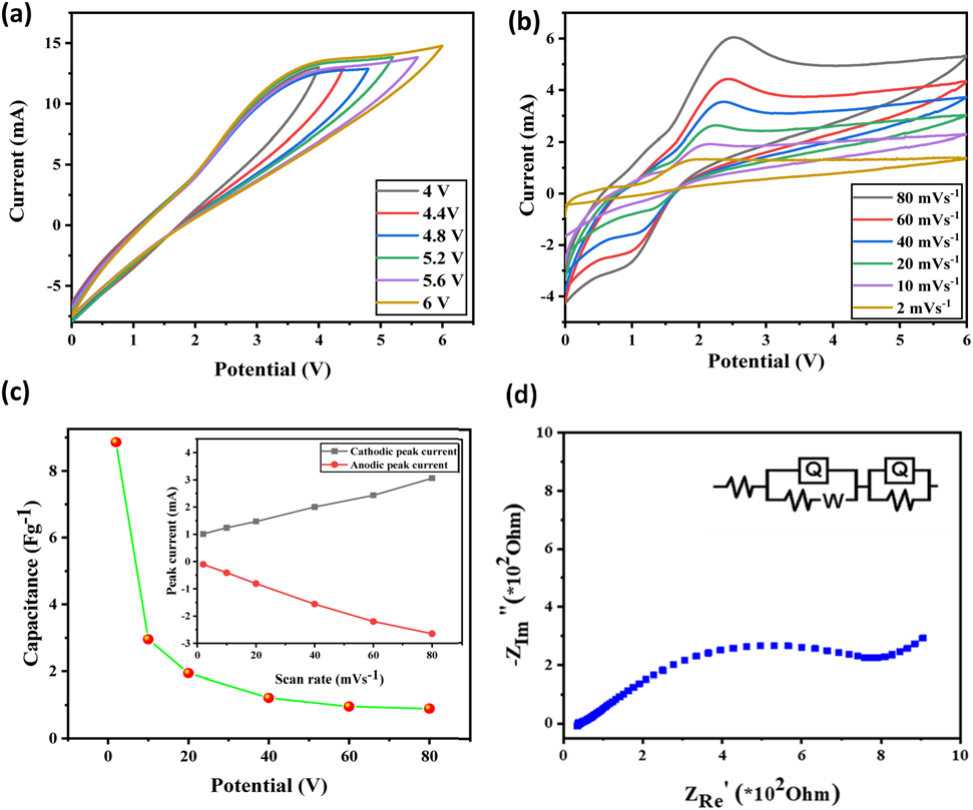
With the graphite-PTFE 2-electrode system and ILGPE (a) cyclic voltammograms at scan rate 100 mV s^−1^, various potentials range from 0–4 to 0–6 V, (b) cyclic voltammetric graphs at different scan rates, (c) graph plotted with areal capacitance against scan rate and inset depicting cathodic and anodic peak current against scan rate, and (d) EIS plots with a most fitting equivalent circuit.

Fig. 9a depicts the cyclic voltammograms recorded at a constant scan rate of 100 mV s^−1^ by varying potential windows for the fabricated capacitor using ILGPE electrodes (1.1304 cm^2^ area) PVDF-HFP polymer containing 1but3pentBTBF_4_ ionic liquid electrolyte. An effective wider working window of 0–6.0 V could be fixed for further analysis. Fig. 9b depicts the cyclic voltammograms obtained at varying scan rates and displays an excellent areal capacitance of 8.85 F g^−1^ at a lower scan rate of 2 mV s^−1^. Fig. 9c shows a gradual capacitance decrease with a rise in scan rate. Faradaic peaks were characterized at varying scan rates. The positions of peaks were not changed much, indicating favorable electron transfer kinetics. In Fig. 9c (inset), cathodic and anodic peak currents with the scan rate increase linearly. The cathodic peak current shows a linearity of 0.999, and the anodic peak current offers a 0.995 linearity. Hence, a absorption-controlled mechanism occurred between the electrodes and ILGPE. The capacitive property of the system is indicated by knee frequency. At this point, the linear curve joins with the region of high frequency, where electrolyte diffusion might have started.^52^

To study the electrode-electrolytic interface most versatile characterization technique used is EIS. Fig. 9d shows the Nyquist impedance plots of the EDLC cells measured in the frequency range 1 Hz–1 MHz at ambient temperature. The behavior indicating double-layer capacitance can be figured through semicircular behavior shown in the high frequency range and the low-frequency range an inclined projection. This is commonly observed in supercapacitors which are carbon electrode-based.^53^ The intercepts of the semicircle on the real axis indicate (*R*_b_ and *R*_ct_), which are the two resistive components. At high-frequency bulk resistance (*R*_b_), an intercept is associated with the ILGPE membrane, which is constant as the ILGPE membrane is common to both EDLCs. The intercept at the low-frequency region is a combination of charge transfer and bulk resistances (*R*_ct_ + *R*_b_). The cumulative electronic and ionic resistance at the interface of electrode–electrolyte gives charge-transfer resistance charge transfer resistance. The resistance arises due to the active electrode layer and current collector contact as well as from electrode particles comprising the electronic resistance, which is minimal as the electrode material has an electronic conductor and current collector graphite is highly electron conducting. Ionic resistance arises due to the resistance encountered by the charge-carrying ions. And the surface morphology, such as pore size and structure of electrodes, significantly influences ionic mobility and resistance due to the charge carrier ions. So ionic resistance is contributing majorly to the charge transfer resistance. The performance of the EDLC can be affected by even a small charge transfer resistance. The interface properties can be better analyzed and understood by the equivalent circuit. Fig. 9d shows the Nyquist plot with an equivalent Randle's circuit.

**Fig. 10:**
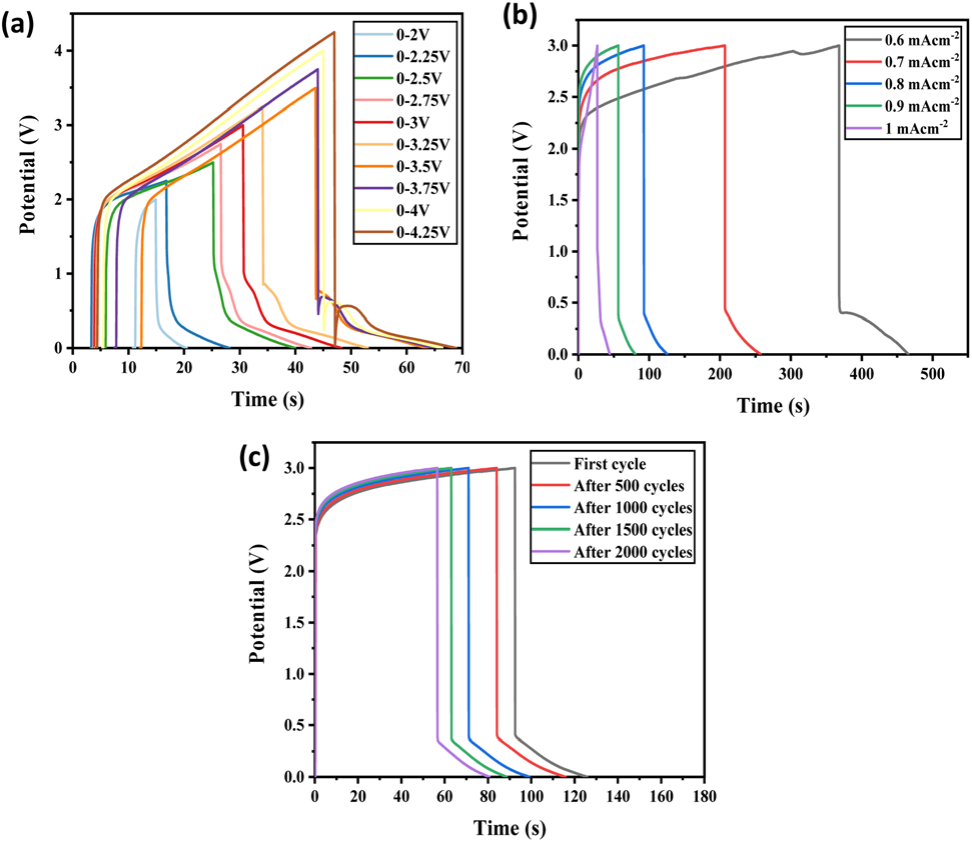
(a) GCD curves with a current density of 1 mA cm^−2^ at different potentials with symmetric two-electrode configuration with graphite-PTFE electrodes and ILGPE (b) charge–discharge curves at different current densities, (c) galvanostatic charge–discharge with a current density 0.7 mA cm^−2^ at various cycle intervals up to 2000 cycles.

The fabricated prototype was tested with galvanostatic charge–discharge (GCD) cycles at a 0–3.0 V constant potential window under various current densities (Fig. 10a). The charge–discharge peaks at various current densities gave an asymmetrical plot showing redox properties. They are smooth graphs without IR drop which indicate their easy charge transfer reaction. This confirms the material's excellent charge storage capacity. Further GCD tested with 0.7 mA cm^−2^ current density were observed at various intervals of cycles. Even after 2000 cycles, the capacitance retention was 71.5% (Fig. S105†). The gradual fading of capacitive retention is due to the depletion happening to a layer of charge forming, and the number of mobile charge carriers is reduced. At the initial stage of charging–discharging, a few of the mobile charge carriers are trapped in the electrode pore, and a depletion layer on the electrode surface or electrode–electrolyte interface is formed. Hence the effective transporting charge carriers are decreased and cause increase in internal resistance with increasing cycles. This must be responsible for the fading of specific capacitance initially. Factors such as electrode properties, electrolyte properties, and device fabrication processes influence the cyclic stability of the device.^54,55^ Here [1but3pentBT]^+^ and [BF_4_]^−^ are the charge-carrying transport ions; few of these ions may get trapped inside the electrode porous structures during the charging and discharging process as they have a bigger radius. For the next charging–discharging cycle, these trapped ions can induce a repulsive force on the same ions. As a result, the device's cyclic stability may reduce gradually as the number of cycles increases. Selecting more suitable and specifically designed electrode materials may be larger pore size can improve the performance of the ILGPE-based EDCL device.^56^

**Fig. 11:**
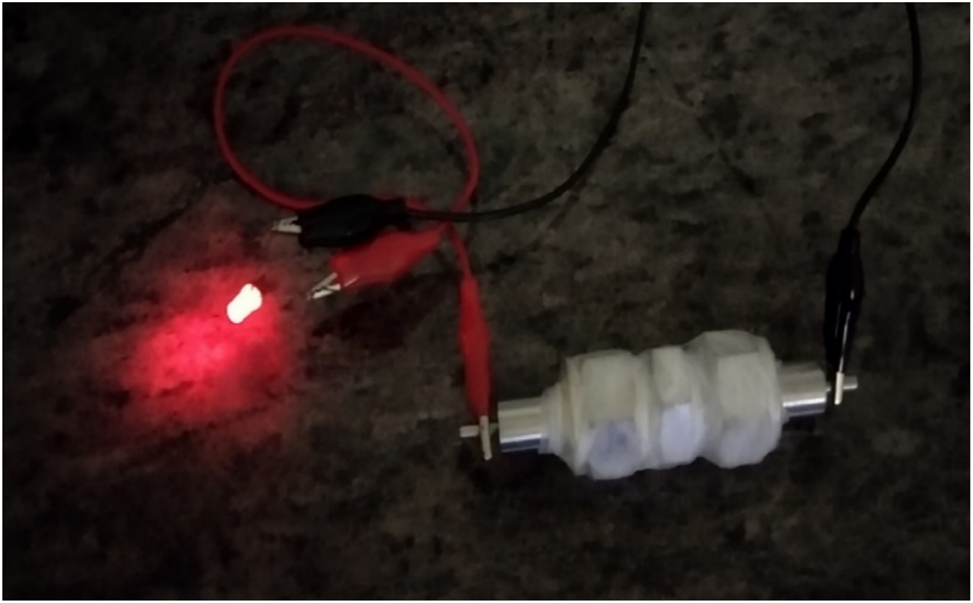
Swagelok cell illustrating the working of red LED-after charging for 10 minutes, after 8 minutes, intensity started to diminish.

A red LED bulb of specifications 20 mA and 2.0 V was made use of to demonstrate the practical potentiality of the prototype device (Fig. 11). One Swagelok cells was sufficient with 10 minutes of charging for glowing red led bulb for 8 min afterward that the intensity of LED light starts to diminish.

## 4. Conclusions

This work reports synthesis, purification, and characterization of asymmetrically substituted 1,3-dialkyl-benzotriazolium-based salts. Further their promising efficiency as electrolytes for fabricating supercapacitors through detailed study of their electrochemical activity. Unprecedented compounds of asymmetrically substituted 1,3-dialkyl-1,2,3-benzotriazolium salts using mild conditions for easy purification is done. This was followed by a detailed evaluation of these ionic liquid's thermal and electrochemical properties. The degradation onset temperatures confirm their stability at elevated temperatures. To derive the electrochemical properties of the ionic liquids synthesized we used a platinum working electrode (2 mm diameter disc) in combination with a platinum wire counter and another platinum pseudo reference electrode. They have a wide electrochemical potential window up to 4.0 V with excellent cyclic recoverability. Few ionic liquid systems are showing better conductance. A novel solid-state ILGPE prepared by entrapping asymmetrically substituted 1-butyl-3-pentyl-1,2,3-benzotriazolium tetrafluoroborate in PVDF-HFP copolymer and studied for its applicability as a solid-state membrane electrolyte in EDLC. The ILGPE was tested with graphite-polytetrafluoroethylene sheets as electrodes and ionic liquid incorporated gel-polymer electrolyte sandwiched in between to form a two-electrode capacitor system. ILGPE has an electrochemical window wide enough up to 0–6 V, and at room temperature a high ionic conductance of 5.71 × 10^−3^ S cm^−1^ and up to ∼200 °C thermal stability. And it shows a high specific capacitance of 8.85 F g^−1^ at a lower scan rate of 2 mV s^−1^. It exhibited an excellent energy density of 2.99 μW h and power density of 11.2 mW g^−1^. Its practical application was demonstrated using a Swagelok cell to power a red LED light for promising real-time supercapacitor fabrication.

This study is a valuable step towards developing a 1,3-dialkyl-1,2,3-benzotriazolium salts-based ionic liquid incorporated ILGPE, which has outstanding potential to be used as a separator/electrolyte in supercapacitors/EDLCs. Future scope of the work will be a potential fabrication of a supercapacitor with more specially designed electrodes suitable for hybrid ionic liquid-based electrolytes from these asymmetrically substituted 1,3-dialkyl-1,2,3-benzotriazolium salts.

## Abbreviations

1propBT: 1-propyl-1,2,3-benzotriazole
1butBT: butyl-1,2,3-benzotriazole
1pentBT: 1-pentyl-1,2,3-benzotriazole
1hexBT: 1-hexyl-1,2,3-benzotriazole
2propBT: 2-propyl-1,2,3-benzotriazole
2butBT: 2-butyl-1,2,3-benzotriazole
2pentBT: 2-pentyl-1,2,3-benzotriazole
2hexBT: 2-hexyl-1,2,3-benzotriazole
1but3propBTBr: 1-butyl-3-propyl-1,2,3-benzotriazolium bromide
1but3propBTBF_4_: 1-butyl-3-propyl-1,2,3-benzotriazolium tetrafluoroborate
1but3propBTPF_6_: 1-butyl-3-propyl-1,2,3-benzotriazolium hexafluorophosphate
1but3pentBTBr: 1-butyl-3-pentyl-1,2,3-benzotriazolium bromide
1but3pentBTBF_4_: 1-butyl-3-pentyl-1,2,3-benzotriazolium tetrafluoroborate
1but3pentBTPF_6_: 1-butyl-3-pentyl-1,2,3-benzotriazolium hexafluorophosphate
1but3hexBTBr: 1-butyl-3-hexyl-1,2,3-benzotriazolium bromide
1but3hexBTBF_4_: 1-butyl-3-hexyl-1,2,3-benzotriazolium tetrafluoroborate
1but3hexBTPF: 1-butyl-3-hexyl-1,2,3-benzotriazolium hexafluorophosphate
1pent3propBTBr: 1-pentyl-3-propyl-1,2,3-benzotriazolium bromide
1pent3propBTBF_4_: 1-pentyl-3-propyl-1,2,3-benzotriazolium tetrafluoroborate
1pent3propBTPF_6_: 1-pentyl-3-propyl-1,2,3-benzotriazolium hexafluorophosphate
hex3propBTBr: 1-hexyl-3-propyl-1,2,3-benzotriazolium bromide
1hex3propBTBF_4_: 1-hexyl-3-propyl-1,2,3-benzotriazolium tetrafluoroborate
hex3propBTPF_6_: 1-hexyl-3-propyl-1,2,3-benzotriazolium hexafluorophosphate
1hex3pentBTBr: 1-hexyl-3-pentyl-1,2,3-benzotriazolium bromide
1hex3pentBTBF_4_: 1-hexyl-3-pentyl-1,2,3-benzotriazolium tetrafluoroborate
1hex3pentBTPF_6_: 1-hexyl-3-pentyl-1,2,3-benzotriazolium hexafluorophosphate
IL: ionic liquid
CV: cyclic voltammetry
TGA: thermogravimetric analysis
DSC: differential scanning calorimetry
EIS: electrochemical impedance spectroscopy
GCD: galvanostatic charge–discharge
PVDF-HFP: poly(vinylidene fluoride-*co*-hexa-fluoropropylene)
ILGPE: ionic liquid incorporated gel electrolyte
EDLC: electric double layer capacitor
PTFE: polytetrafluoroethylene
LED: light-emitting diode

## Author contributions

Anjitha Satheesh: design, synthesis, methodology, investigation, data curation, writing – original draft. Punnakkal Navaneeth: electrochemical studies discussion, synthesis of electrode material. Punathil Vasu Suneesh: electrochemical studies discussion and evaluation, manuscript correction. Sarath chandran C: initial discussion on cyclic voltammetry of ionic liquids. Elango Kandasamy: conceptualization, supervision, NMR and electrochemical studies discussion, and manuscript correction.

## Conflicts of interest

The authors declare no conflict of interest, financial or otherwise.

## Supplementary Material

RA-013-D3RA01958F-s001
