# Supplementary material for: Synthesis, characterization and study of electrochemical applicability of novel asymmetrically substituted 1,3-dialkyl-1,2,3-benzotriazolium salts for supercapacitor fabrication
Source: RSC Adv. 2023 May 15;13(22):14737–46. doi: 10.1039/d3ra01958f (PMC10184001; doi:10.1039/d3ra01958f)
Supplement: RA-013-D3RA01958F-s001 [file RA-013-D3RA01958F-s001.pdf]

Electronic Supplementary Material (ESI) for RSC Advances.
This journal is © The Royal Society of Chemistry 2023

Supplementary material

Synthesis, characterization and study of electrochemical applicability of novel asymmetrically substituted 1,3-dialkyl-1,2,3-benzotriazolium salts for supercapacitor fabrication

Anjitha Satheesh[a], Punnakkal Navaneeth[a,b], Punathil Vasu Suneesh[a,b], Sarathchandran C[c] Elango Kandasamy[a,]*

[a]Department of Sciences, Amrita School of Physical Sciences Coimbatore, Amrita Vishwa Vidyapeetham, 641112, India

[b]Amrita Biosensor Research Lab, Amrita School of Physical Sciences Coimbatore, Amrita Vishwa Vidyapeetham, 641112, India.

[c]Department of Sciences, Amrita School of Engineering, Chennai, Amrita Vishwa Vidyapeetham, India.

S1. List of Abbreviations

1) 1propBT - 1-propyl-1,2,3-benzotriazole

2) 1butBT - 1-butylyl-1,2,3-benzotriazole

3) 1pentBT - 1-pentyl-1,2,3-benzotriazole

4) 1hexBT - 1-hexyl-1,2,3-benzotriazole

5) 2propBT - 2-propyl-1,2,3-benzotriazole

6) 2butBT - 2-butylyl-1,2,3-benzotriazole

7) 2pentBT - 2-pentyl-1,2,3-benzotriazole

8) 2hexBT - 2-hexyl-1,2,3-benzotriazole

9) 1but3propBTBr - 1-butyl-3-propyl-1,2,3-benzotriazolium bromide

10) 1but3propBTBF$_4$ - 1-butyl-3-propyl-1,2,3-benzotriazolium tetrafluoroborate

11) 1but3propBTPF$_6$ - 1-butyl-3-propyl-1,2,3-benzotriazolium hexafluorophosphate

12) 1but3pentBTBr - 1-butyl-3-pentyl-1,2,3-benzotriazolium bromide

13) 1but3pentBTBF$_4$ - 1-butyl-3-pentyl-1,2,3-benzotriazolium tetrafluoroborate

14) 1but3pentBTPF$_6$ - 1-butyl-3-pentyl-1,2,3-benzotriazolium hexafluorophosphate

15) 1but3hexBTBr - 1-butyl-3-hexyl-1,2,3-benzotriazolium bromide

16) 1but3hexBTBF$_4$ - 1-butyl-3-hexyl-1,2,3-benzotriazolium tetrafluoroborate

17) 1but3hexBTPF$_6$ - 1-butyl-3-hexyl-1,2,3-benzotriazolium hexafluorophosphate

18) 1pent3propBTBr - 1-pentyl-3-propyl-1,2,3-benzotriazolium bromide

19) 1pent3propBTBF$_4$ - 1-pentyl-3-propyl-1,2,3-benzotriazolium tetrafluoroborate

20) 1pent3propBTPF$_6$ - 1-pentyl-3-propyl-1,2,3-benzotriazolium hexafluorophosphate

21) 1hex3propBTBr - 1-hexyl-3-propyl-1,2,3-benzotriazolium bromide

22) 1hex3propBTBF$_4$ - 1-hexyl-3-propyl-1,2,3-benzotriazolium tetrafluoroborate

23) 1hex3propBTPF$_6$ - 1-hexyl-3-propyl-1,2,3-benzotriazolium hexafluorophosphate

24) 1hex3pentBTBr - 1-hexyl-3-pentyl-1,2,3-benzotriazolium bromide

25) 1hex3pentBTBF$_4$ - 1-hexyl-3-pentyl-1,2,3-benzotriazolium tetrafluoroborate

26) 1hex3pentBTPF$_6$ - 1-hexyl-3-pentyl-1,2,3-benzotriazolium hexafluorophosphate

27) IL - Ionic liquid

28) CV - Cyclic voltammetry

29) TGA - Thermogravimmetric analysis

30) DSC - Differential scanning calorimetry

31) EIS - Electrochemical impedance spectroscopy

32) GCD - Galvanostatic charge-discharge

33) PVDF-HFP - Poly(vinylidene fluoride-co-hexa-fluoropropylene)

34) ILGPE - Ionic liquid incorporated gel electrolyte

35) EDLC - Electrochemical double layer supercapacitors

36) PTFE - Polytetrafluoroethylene

37) LED - Light-emitting diode

S2. NMR details of 1,3-dialkyl-1,2,3-benzotriazolium salt

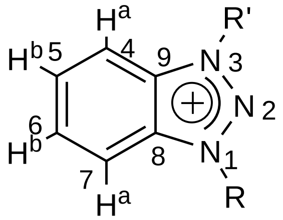

Figure S1. Numbering of 1,3-dialkyl-1,2,3-benzotriazolium salt

Table S1. Important ^{1}H NMR and ^{13}C NMR ppm values of 1,3-dialkyl-1,2,3-benzotriazolium salt

SL. No	Compounds	H^a (ppm)	H^b (ppm)	H^c (ppm)	H^d (ppm)	C-8 (ppm)	C-9 (ppm)	C-7 (ppm)	C-4 (ppm)	C-6 (ppm)	C-5(ppm)
1	1propBT	8.04	7.53	7.45	7.34	145.87	132.94	127.07	123.7	117.6	109.4
2	2propBT	7.86	7.86	7.33	7.33	144.23	144.23	126	126	117.87	117.87
3	1butBT	8.04	7.53	7.45	7.34	145.89	132.88	127.03	123.67	119.77	109.37
4	2butBT	7.86	7.86	7.34	7.34	144.24	144.24	126.05	126.05	117.43	117.43

5	1pentBT	8.11	7.93	7.59	7.54	150.44	138	132.16	128.89	124.23	115.59
6	2pentBT	7.98	7.98	7.45	7.45	148.95	148.95	131.12	131.12	122.89	122.89
7	1hexBT	8.12	7.92	7.58	7.44	150.49	137.98	132.05	128.78	124.26	115.39
8	2hexBT	7.96	7.26	7.44	7.44	148.92	148.92	131.18	131.18	122.89	122.89
9	1but3propBT Br	8.69	8.69	7.99	7.99	134.68	134.68	131.48	131.48	114.22	114.22
10	1but3propBT BF_4	8.41	8.41	7.93	7.93	132.9	132.9	129.84	129.84	112.18	112.18
11	1but3propBT PF_6	8.06	8.06	7.97	7.97	134.77	134.77	131.66	131.66	113.42	113.42
12	1but3pentBT Br	8.62	8.62	7.96	7.96	134.57	134.57	131.61	131.61	114.29	114.29
13	1but3pentBT BF_4	8.41	8.41	7.93	7.93	132.87	132.87	129.86	129.86	112.16	112.16
14	1but3pentBTP F_6	8.11	8.11	7.87	7.87	134.74	134.74	131.69	139.69	113.5	113.5
15	1but3hexBTB r	8.67	8.67	7.96	7.96	134.65	134.65	131.52	131.52	114.44	114.44
16	1but3hexBTB F_4	8.13	8.13	7.65	7.65	134.53	134.53	131.5	131.5	113.81	113.81
17	1but3hexBTP F_6	8.07	8.07	7.86	7.86	134.73	134.73	131.67	131.67	113.4	113.4
18	1pent3propBT Br	8.62	8.62	7.97	7.97	134.55	`134.55	131.56	131.56	114.2	114.2
19	1pent3propBT BF_4	7.92	7.92	7.41	7.41	134.57	134.57	131.53	131.53	113.9	113.9
20	1pent3propBT PF_6	8.09	8.09	7.87	7.87	134.76	134.76	131.66	131.66	113.45	113.45
21	1hex3propBT Br	8.02	8.02	7.3	7.3	134.4	134.4	131.41	131.41	114.17	114.17
22	1hex3propBT BF_4	8.39	8.39	7.92	7.92	134.7	134.7	131.64	131.64	113.93	113.93
23	1hex3propBT PF_6	8.09	8.09	7.86	7.86	133.25	133.25	131.66	131.66	113.45	113.45

24	1hex3pentBT Br	8.68	8.68	7.99	7.99	134.94	134.94	131.44	131.44	114.15	114.15
25	1hex3pentBT BF_4	8.1	8.1	7.61	7.61	138	138	129.83	129.83	112.17	112.17
26	1hex3pentBT PF_6	8.06	8.06	7.86	7.86	134.74	134.74	131.6	131.6	113.32	113.32

1-propyl-1,2,3-benzotriazole(1propBT)

1H-NMR ($CDCl_3$, 400 MHz): 8.04 (dt, 1H, Ar), 7.53 (dt, 1H, Ar), 7.45 (m, 1H, Ar) , 7.34(m, 1H, Ar) ,4.58 (t, 2H, *CH_2*-CH_2-CH_3), 2.02 (m, 2H, , CH_2-*CH_2*-CH_3), 0.94 (t, 3H, -CH_2-CH_2-*CH_3*). 13C NMR ($CDCl_3$, 100 MHz): 145.87(C-8), 132.94(C-9), 127.07(C-7), 123.70(C-4), 119.60(C-6), 109.40(C-5), 49.68(*C*H_2-CH_2-CH_3), 23.05(CH_2-*C*H_2-CH_3), 11.22(CH_2-CH_2-*C*H_3).

2-propyl-1,2,3-benzotriazole(2propBT)

1H-NMR ($CDCl_3$, 400 MHz): 7.86 (dd, 2H, Ar), 7.33 (dd, 2H, Ar), 4.67 (t, 2H, *CH_2*-CH_2-CH_3), 2.12 (m, 2H, CH_2-*CH_2*-CH_3), 0.94 (t, 3H, -CH_2-CH_2-*CH_3*). 13C NMR ($CDCl_3$, 100 MHz): 144.23(C-8, C-9), 126(C-7, C-4), 117.87(C-5, C-6), 58.01 (*C*H_2-CH_2-CH_3), 23.41(CH_2-*C*H_2-CH_3), 10.84(CH_2-CH_2-*C*H_3).

1-butyl -1,2,3-benzotriazole(1butBT)

^{1}H-NMR ($CDCl_3$, 400 MHz): 8.04 (dt, 1H, Ar), 7.53 (dt, 1H, Ar), 7.45 (m, 1H, Ar) ,7.34 (m, 1H, Ar) 4.62 (t, 2H, *CH_2*-CH_2-CH_2-CH_3), 1.97 (m, 2H, CH_2-*CH_2*-CH_2-CH_3), 1.31 (m, 3H, CH_2-CH_2-*CH_2*-CH_3), 0.93 (t, 3H, CH_2-CH_2-CH_2-*CH_3*). ^{13}C NMR ($CDCl_3$, 100 MHz): 145.89 (C-8), 132.88 (C-9), 127.03 (C-7), 123.67 (C-4), 119.77 (C-6), 109.37 (C-5), 47.83 (*C*H_2-CH_2-CH_2-CH_3), 31.58 (CH_2-*C*H_2-CH_2-CH_3), 19.58 (CH_2-CH_2-*C*H_2-CH_3), 13.22 (CH_2-CH_2-CH_2-*C*H_3).

2-butyl-1,2,3-benzotriazole(2butBT)

^{1}H-NMR ($CDCl_3$, 400 MHz): 7.86 (dd, 2H, Ar), 7.34 (dd, 2H, Ar), 4.71 (t, 2H, *CH_2*-CH_2-CH_2-CH_3), 2.09 (m, 2H, CH_2-*CH_2*-CH_2-CH_3), 1.36 (m, 2H, CH_2-CH_2-*CH_2*-CH_3), 0.95 (t, 3H, CH_2-CH_2-CH_2-*CH_3*).

13C NMR ($CDCl_3$, 100 MHz): ^{13}C NMR ($CDCl_3$, 100 MHz): 144.24 (C-8,C-9), 126.05 (C-7, C-4), 117.43 (C-5, C-6), 56.24 ($\mathit{C}H_2$-CH_2-CH_2-CH_3), 31.97 (CH_2-$\mathit{C}H_2$-CH_2-CH_3), 19.73 (CH_2-CH_2-$\mathit{C}H_2$-CH_3), 13.43 (CH_2-CH_2-CH_2-$\mathit{C}H_3$).

1-pentyl-1,2,3-benzotriazole(1pentBT)

^{1}H-NMR ($CDCl_3$, 400 MHz): 8.11 (dt, 1H, Ar), 7.93 (dt, 1H, Ar), 7.59 (m, 1H, Ar) ,7.54 (m, 1H, Ar), 4.76 (t, 2H, $\mathit{CH_2}$-CH_2-CH_2-CH_2-CH_3), 1.95 (m, 2H, CH_2-$\mathit{CH_2}$-CH_2-CH_2-CH_3), 1.26 (m, 3H, CH_2-CH_2-$\mathit{CH_2}$-$\mathit{CH_2}$-CH_3), 0.82 (t, 3H, CH_2-CH_2-CH_2-CH_2-$\mathit{CH_3}$). ^{13}C NMR ($CDCl_3$, 100 MHz): 150.44 (C-8), 138 (C-9), 132.16 (C-7), 128.89 (C-4), 124.28 (C-6), 115.59 (C-5), 52.58 ($\mathit{C}H_2$-CH_2-CH_2-CH_2CH_3), 34.12 (CH_2-$\mathit{C}H_2$-CH_2-CH_2-CH_3), 33.38 (CH_2-CH_2-$\mathit{C}H_2$-CH_2-CH_3), 26.73 (CH_2-CH_2-CH_2-$\mathit{C}H_2$-CH_3), 18.78 (CH_2-CH_2-CH_2-CH_2-$\mathit{C}H_3$).

2-pentyl-1,2,3-benzotriazole(2pentBT)

^{1}H-NMR ($CDCl_3$, 400 MHz): 7.98 (dd, 2H, Ar), 7.45 (dd, 2H, Ar), 4.78 (t, 2H, $\mathit{CH_2}$-CH_2-CH_2-CH_2-CH_3), 2.07 (m, 2H, CH_2-$\mathit{CH_2}$-$\mathit{CH_2}$-CH_2-CH_3), 1.28 (m, 4H, CH_2-CH_2-CH_2-$\mathit{CH_2}$-CH_3), 0.83 (t, 3H, CH_2-CH_2-CH_2-$\mathit{CH_3}$). ^{13}C NMR ($CDCl_3$, 100 MHz):148.95 (C-8, C-9), 131.12 (C-7, C-4), 122.89 (C-5, C-6), 61.08 ($\mathit{C}H_2$-CH_2-CH_2-CH_2-CH_3), 34.31 (CH_2-$\mathit{C}H_2$-CH_2-CH_2-CH_3), 33.29 (CH_2-CH_2-$\mathit{C}H_2$-CH_2-CH_3), 26.72 (CH_2-CH_2-CH_2-$\mathit{C}H_2$-CH_3), 18.69 (CH_2-CH_2-CH_2-CH_2-$\mathit{C}H_3$).

1-hexyl-1,2,3-benzotriazole(1hexBT)

^{1}H-NMR ($CDCl_3$, 400 MHz): 8.12 (dt, 1H, Ar), 7.92 (dt, 1H, Ar), 7.58 (m, 1H, Ar) ,7.44 (m, 1H, Ar), 4.77 (t, 2H, $\mathit{CH_2}$-CH_2-CH_2-CH_2-CH_2-CH_3), 1.95 (m, 2H, CH_2-$\mathit{CH_2}$-CH_2-CH_2-CH_2-CH_3), 1.23 (m, 4H, CH_2-CH_2-$\mathit{CH_2}$-$\mathit{CH_2}$-$\mathit{CH_2}$-CH_3), 0.80 (t, 3H, CH_2-CH_2-CH_2-CH_2-CH_2-$\mathit{CH_3}$). ^{13}C NMR ($CDCl_3$, 100 MHz):150.49 (C-8), 137.98 (C-9), 132.05 (C-7), 128.78 (C-4)124.26 (C-5), 115.39 (C-6), 52.60 ($\mathit{C}H_2$-CH_2-CH_2-CH_2-CH_2-CH_3), 35.79 (CH_2-$\mathit{C}H_2$-CH_2-CH_2-CH_2-CH_3), 34.39 (CH_2-CH_2-$\mathit{C}H_2$-CH_2-CH_2-CH_3),

30.89 (CH$_2$-CH$_2$-CH$_2$-*C*H$_2$-CH$_2$-CH$_3$), 27.06 (CH$_2$-CH$_2$-CH$_2$-CH$_2$-*C*H$_2$-CH$_3$), 18.74 (CH$_2$-CH$_2$-CH$_2$-CH$_2$-CH$_2$-*C*H$_3$).

2-hexyl-1,2,3-benzotriazole(2hexBT)

^{1}H-NMR (CDCl$_3$, 400 MHz): 7.96 (dd, 2H, Ar), 7.44 (dd, 2H, Ar), 4.77 (t, 2H, *CH*$_2$-CH$_2$-CH$_2$-CH$_2$-CH$_2$-CH$_3$), 2.05 (m, 2H, CH$_2$-*CH*$_2$-CH$_2$-CH$_2$-CH$_2$-CH$_3$), 1.25 (m, 4H, CH$_2$-CH$_2$-*CH*$_2$-*CH*$_2$-*CH*$_2$-CH$_3$), 0.82 (t, 3H, CH$_2$-CH$_2$-CH$_2$-*CH*$_3$). ^{13}C NMR (CDCl$_3$, 100 MHz):148.92 (C-8, C-9), 131.18 (C-7, C-4), 122.89 (C-5, C-6), 61.10 (*C*H$_2$-CH$_2$-CH$_2$-CH$_2$-CH$_2$-CH$_3$), 35.79 (CH$_2$-*C*H$_2$-CH$_2$-CH$_2$-CH$_2$-CH$_3$), 34.55 (CH$_2$-CH$_2$-*C*H$_2$-CH$_2$-CH$_2$-CH$_3$), 30.80 (CH$_2$-CH$_2$-CH$_2$-*C*H$_2$-CH$_2$-CH$_3$), 27.06 (CH$_2$-CH$_2$-CH$_2$-CH$_2$-*C*H$_2$-CH$_3$), 18.82 (CH$_2$-CH$_2$-CH$_2$-CH$_2$-CH$_2$-*C*H$_3$).

1-butyl-3-propyl-1,2,3-benzotriazolium bromide(1but3propBTBr)

1H-NMR (CDCl$_3$, 400 MHz):8.69 (m, 2H, Ar), 7.99 (m, 2H,Ar), 5.26 (m, 4H, *CH*$_2$-CH$_2$-CH$_3$, *CH*$_2$-CH$_2$-CH$_2$-CH$_3$), 2.20 (m, 2H,CH$_2$-*CH*$_2$-CH$_3$), 1.48 (m, 4H,CH$_2$-*CH*$_2$-*CH*$_2$-CH$_3$), 1.07 (t, 1H,CH$_2$-CH$_2$-*CH*$_3$), 0.99 (t, 1H,CH$_2$-CH$_2$-CH$_2$-*CH*$_3$). 13C NMR (CDCl$_3$, 100 MHz): 134.68(C-8, C-9), 131.48 (C-4, C-7), 114.22(C-5, C-6), 53.91 (*C*H$_2$-CH$_2$-CH$_3$), 52.29 (*C*H$_2$-CH$_2$-CH$_2$-CH$_3$), 30.77 (CH$_2$-*C*H$_2$-CH$_3$), 22.51 (CH$_2$-*C*H$_2$-CH$_2$-CH$_3$), 19.26 (CH$_2$-CH$_2$-*C*H$_2$-CH$_3$), 13.11(CH$_2$-CH$_2$-*CH*$_3$), 10.70(CH$_2$-CH$_2$-CH$_2$-*CH*$_3$).

1-butyl-3-propyl-1,2,3-benzotriazolium tetrafluoroborate(1but3propBTBF$_4$)

1H-NMR (CDCl$_3$, 400 MHz):8.41 (m, 2H, Ar), 7.93 (m, 2H, Ar), 5.08 (m, 4H, *CH*$_2$-CH$_2$-CH$_3$, *CH*$_2$-CH$_2$-CH$_2$-CH$_3$), 2.17 (m, 2H,CH$_2$-*CH*$_2$-CH$_3$), 1.45 (m, 4H,CH$_2$-*CH*$_2$-*CH*$_2$-CH$_3$), 1.05 (t, 1H,CH$_2$-CH$_2$-*CH*$_3$), 0.98 (t, 1H, CH$_2$-CH$_2$-CH$_2$-*CH*$_3$). 13C NMR (CDCl$_3$, 100 MHz): 132.90 (C-8, C-9), 129.84 (C-4, C-7),112.18 (C-5, C-6), 51.91(*C*H$_2$-CH$_2$-CH$_3$), 50.26 (*C*H$_2$-CH$_2$-CH$_2$-CH$_3$), 29.04 (CH$_2$-*C*H$_2$-CH$_3$), 20.78 (CH$_2$-*C*H$_2$-CH$_2$-CH$_3$), 17.67 (CH$_2$-CH$_2$-*C*H$_2$-CH$_3$), 11.48 (CH$_2$-CH$_2$-*CH*$_3$), 9.02 (CH$_2$-CH$_2$-CH$_2$-*CH*$_3$).

1-butyl-3-propyl-1,2,3-benzotriazolium hexafluorophosphate(1but3propBTPF$_6$)

1H-NMR ($CDCl_3$, 400 MHz):8.06 (m, 1H, Ar), 7.97 (m, 2H,Ar), 4.88 (s, 4H, *CH2*-CH2-CH3, *CH2*-CH2-CH2-CH3), 2.13 (m, 2H,CH2-*CH2*-CH3), 1.53(m, 4H,CH2-*CH2*-*CH2*-CH3), 1.01 (t, 1H,CH2-CH2-*CH3*), 0.98 (t, 1H, CH2-CH2-CH2-*CH3*). 13C NMR ($CDCl_3$, 100 MHz): 134.77 (C-8, C-9), 131.66 (C-4, C-7),113.42 (C-5, C-6), 53.53 (*C*H2-CH2-CH3), 51.85 (*C*H2-CH2-CH2-CH3), 30.74 (CH2-*C*H2-CH3), 22.46 (CH2-*C*H2-CH2-CH3), 19.65 (CH2-CH2-*C*H2-CH3), 13.29 (CH2-CH2-*C*H3), 10.90 (CH2-CH2-CH2-*CH3*).

1-hexyl-3-propyl-1,2,3-benzotriazolium bromide(1hex3propBTBr)

^{1}H-NMR (CDCl3, 400 MHz):8.01 (m,2H,Ar), 7.29 (m, 2H,Ar), 4.57 (t, 4H, *CH2*-CH2-CH3,*CH2*-CH2-CH2-CH2-CH2-CH3), 1.51 (m, 4H,CH2-*CH2*-CH3, CH2-*CH2*-CH2-CH2-CH2-CH3), 0.64 (m, 6H, CH2-CH2-*CH2*-*CH2*-*CH2*-CH3), 0.37 (CH2-CH2-*CH3*) 0.16 (CH2-CH2-CH2-CH2-CH2-*CH3*). ^{13}C NMR ($CDCl_3$, 100 MHz): 134.40 (C-8, C-9), 131.41 (C-4, C-7), 114.17 (C-5, C-6), 53.83 (*C*H2-CH2-CH2-CH2-CH2-CH3), 52.46 (*C*H2-CH2-CH3,), 30.52 (CH2-*C*H2-CH2-CH2-CH2-CH3), 28.72 (CH2-CH2-*C*H2-CH2-CH2-CH3), 25.50 (CH2-CH2-CH2-*C*H2-CH2-CH3), 22.46 (CH2-*C*H2-CH3), 22.14 (CH2-CH2-CH2-CH2-*C*H2-CH3), 13.50 (CH2-CH2-*C*H3), 10.61 (CH2-CH2-CH2-CH2-CH2-CH2-*C*H3).

1-hexyl-3-propyl-1,2,3-benzotriazolium tetrafluoroborate(1hex3propBTBF$_4$)

^{1}H-NMR ($CDCl_3$, 400 MHz): 8.39 (m, 2H,Ar), 7.92 (m, 2H, Ar), 5.07 (m, 4H,*CH2*-CH2-CH2-CH2-CH2-CH3,*CH2*-CH2-CH3), 2.17 (m,2H, CH2-*CH2*-CH2-CH2-CH2-CH3), 1.33 (m, 4H, CH2-CH2-*CH2*-*CH2*-CH2-CH3), 1.04 (t, 3H, , CH2-CH2-*CH3*), 0.86 (t, 3H, CH2-CH2-CH2-CH2-CH2-*CH3*). ^{13}C NMR ($CDCl_3$, 100 MHz): 134.7 (C-8, C-9), 131.64 (C-4, C-7), 113.93 (C-5, C-6), 53.72 (*C*H2-CH2-CH3), 52.32 (*C*H2-CH2-CH2-CH2-CH2-CH3), 30.82 (CH2-*C*H2-CH2-CH2-CH2-CH3), 28.87 (CH2-CH2-*C*H2-CH2-CH2-CH3), 25.82 (CH2-CH2-CH2-*C*H2-CH2-CH3), 22.57(CH2-*C*H2-CH3), 22.16 (CH2-CH2-CH2-CH2-*C*H2-CH3),13.77 (CH2-CH2-*CH3*), 10.80 (CH2-CH2-CH2-CH2-CH2-*CH3*).

1-hexyl-3-propyl-1,2,3-benzotriazolium hexafluorophosphate(1hex3propBTPF$_6$)

^{1}H-NMR ($CDCl_3$, 400 MHz): 8.09 (m, 2H, Ar), 7.86 (m, 2H, Ar), 4.90 (m, 4H, *CH_2*-CH_2-CH_2-CH_2-CH_2-CH_3, *CH_2*-CH_2-CH_3), 2.13 (m, 2H, CH_2-*CH_2*-CH_2-CH_2-CH_2-CH_3), 1.32 (m, 4H, CH_2-CH_2-*CH_2*-*CH_2*-CH_2-CH_3), 1.02 (t, 3H, , CH_2-CH_2-*CH_3*), 0.86 (t, 3H, CH_2-CH_2-CH_2-CH_2-CH_2-*CH_3*). ^{13}C NMR ($CDCl_3$, 100 MHz): 133.25 (C-8, C-9), 131.66 (C-4, C-7), 113.45 (C-5, C-6), 53.45 (*C*H_2-CH_2-CH_3, *C*H_2-CH_2-CH_2-CH_2-CH_2-CH_3), 52.03 (CH_2-*C*H_2-CH_2-CH_2-CH_2-CH_3), 30.91 (CH_2-CH_2-*C*H_2-CH_2-CH_2-CH_3), 28.79 (CH_2-CH_2-CH_2-*C*H_2-CH_2-CH_3), 25.94 (CH_2-*C*H_2-CH_3), 22.46 (CH_2-CH_2-CH_2-CH_2-*C*H_2-CH_3), 13.85 (CH_2-CH_2-*CH_3*), 10.82 (CH_2-CH_2-CH_2-CH_2-CH_2-*CH_3*).

1-butyl-3-hexyl-1,2,3-benzotriazolium bromide(1but3hexBTBr)

^{1}H-NMR ($CDCl_3$, 400 MHz):8.67 (m, 2H,Ar), 7.96 (m, 2H,Ar), 5.27 (m, 4H, *CH_2*-CH_2-CH_2-CH_3,*CH_2*-CH_2-CH_2-CH_2-CH_2-CH_3), 2.16 (m, 4H,CH_2-*CH_2*-*CH_2*-CH_3), 1.39 (m, 8H, CH_2-*CH_2*-*CH_2*-*CH_2*-*CH_2*-CH_3), 0.99 (t, 3H, CH_2-CH_2-CH_2-*CH_3*), 0.86 (t, 3H, CH_2-CH_2-CH_2-CH_2-CH_2-CH_2-*CH_3*). ^{13}C NMR (CDCl3, 100 MHz): 134.65 (C-8, C-9), 131.52 (C-4, C-7), 114.44 (C-5, C-6), 52.72 (*CH_2*-CH_2-CH_2-CH_3), 52.48 (*CH_2*-CH_2-CH_2-CH_2-CH_2-CH_3), 30.99 (CH_2-*CH_2*-CH_2-CH_3), 30.76 (CH_2-*CH_2*-CH_2-CH_2-CH_2-CH_3), 28.99 (CH_2-CH_2-*CH_2*-CH_2-CH_2-CH_3), 25.76 (CH_2-CH_2-CH_2-*CH_2*-CH_2-CH_3), 22.08 (CH_2-CH_2-*CH_2*-CH_3), 19.43 (CH_2-CH_2-CH_2-CH_2-*CH_2*-CH_3),13.69 (CH_2-CH_2-CH_2-*C*H_3), 13.23 (CH_2-CH_2-CH_2-CH_2-CH_2-*C*H_3).

1-butyl-3-hexyl-1,2,3-benzotriazolium tetrafluoroborate(1but3hexBTBF$_4$)

^{1}H-NMR ($CDCl_3$, 400 MHz):8.13 (m, 2H,Ar), 7.65 (m, 2H,Ar), 4.82 (m, 4H, *CH_2*-CH_2-CH_2-CH_3,*CH_2*-CH_2-CH_2-CH_2-CH_2-CH_3), 1.86 (m, 4H,CH_2-*CH_2*-*CH_2*-CH_3), 1.08 (m, 8H, CH_2-*CH_2*-*CH_2*-*CH_2*-*CH_2*-CH_3), 0.69 (t, 3H, CH_2-CH_2-CH_2-*CH_3*), 0.57 (t, 3H, CH_2-CH_2-CH_2-CH_2-CH_2-CH_2-*CH_3*). ^{13}C NMR (CDCl3, 100 MHz): 134.53 (C-8, C-9), 131.50 (C-4, C-7), 113.81 (C-5, C-6), 52.13 (*CH_2*-CH_2-CH_2-CH_3), 51.88 (*CH_2*-CH_2-CH_2-CH_2-CH_2-CH_3), 30.70 (CH_2-*CH_2*-CH_2-CH_3, CH_2-*CH_2*-CH_2-CH_2-CH_2-CH_3), 28.71 (CH_2-CH_2-*CH_2*-CH_2-CH_2-CH_3), 25.66 (CH_2-CH_2-CH_2-*CH_2*-CH_2-CH_3), 22.03 (CH_2-CH_2-

$\mathit{CH_2}$-CH_3), 19.31 (CH_2-CH_2-CH_2-CH_2-$\mathit{CH_2}$-CH_3),13.64 (CH_2-CH_2-CH_2-$\mathit{C}H_3$), 13.13 (CH_2-CH_2-CH_2-CH_2-CH_2-$\mathit{C}H_3$).

1-butyl-3-hexyl-1,2,3-benzotriazolium hexafluorophosphate(1but3hexBTPF$_6$)

^{1}H-NMR ($CDCl_3$, 400 MHz):8.07 (m, 2H,Ar), 7.86 (m, 2H,Ar), 4.89 (m, 4H, $\mathit{CH_2}$-CH_2-CH_2-CH_3,$\mathit{CH_2}$-CH_2-CH_2-CH_2-CH_2-CH_3), 2.10 (m, 2H, CH_2-$\mathit{CH_2}$-$\mathit{CH_2}$-CH_3), 1.36 (m, 8H,CH_2-$\mathit{CH_2}$-$\mathit{CH_2}$-$\mathit{CH_2}$-$\mathit{CH_2}$-CH_3), 0.97 (t, 1H, CH_2-CH_2-CH_2-$\mathit{CH_3}$), 0.86 (m, 1H, CH_2-CH_2-CH_2-CH_2-CH_2-$\mathit{CH_3}$). ^{13}C NMR (CDCl3, 100 MHz): 134.73(C-8, C-9), 131.67 (C-4, C-7), 113.40 (C-5, C-6), 52.00 ($\mathit{CH_2}$-CH_2-CH_2-CH_3), 51.75 ($\mathit{CH_2}$-CH_2-CH_2-CH_2-CH_2-CH_3), 30.81 (CH_2-$\mathit{CH_2}$-CH_2-CH_3, CH_2-$\mathit{CH_2}$-CH_2-CH_2-CH_2-CH_3), 28.77 (CH_2-CH_2-$\mathit{CH_2}$-CH_2-CH_2-CH_3), 25.93 (CH_2-CH_2-CH_2-$\mathit{CH_2}$-CH_2-CH_3), 22.27 (CH_2-CH_2-$\mathit{CH_2}$-CH_3), 19.56 (CH_2-CH_2-CH_2-CH_2-$\mathit{CH_2}$-CH_3), 13.85 (CH_2-CH_2-CH_2-$\mathit{C}H_3$), 13.27 (CH_2-CH_2-CH_2-CH_2-CH_2-$\mathit{C}H_3$).

1-hexyl-3-pentyl -1,2,3-benzotriazolium bromide(1hex3pentBTBr)

^{1}H-NMR ($CDCl_3$, 400 MHz): 8.00 (m, 2H,Ar), 7.29 (m, 2H, Ar), 4.59 (m, 4H, $\mathit{CH_2}$-CH_2-CH_2-CH_2-CH_2-CH_3, $\mathit{CH_2}$-CH_2-CH_2-CH_2-CH_3), 1.49 (m, 4H, CH_2-$\mathit{CH_2}$-$\mathit{CH_2}$-CH_2-CH_3,), 0.64 (m, 6H, CH_2-$\mathit{CH_2}$-$\mathit{CH_2}$-$\mathit{CH_2}$-CH_2-CH_3), 0.17 (m, 6H,CH_2-CH_2-CH_2-CH_2-$\mathit{CH_3}$, CH_2-CH_2-CH_2-CH_2-CH_2-$\mathit{CH_3}$). ^{13}C NMR ($CDCl_3$, 100 MHz): 134.94 (C-8, C-9),131.44 (C-4, C-7), 114.15 (C-5, C-6), 52.92 ($\mathit{C}H_2$-CH_2-CH_2-CH_2-CH_3), 30.69 ($\mathit{C}H_2$-CH_2-CH_2-CH_2-CH_2-CH_3,), 28.89 (CH_2-$\mathit{C}H_2$-CH_2-CH_2-CH_3,), 28.64 (CH_2-$\mathit{C}H_2$-CH_2-CH_2-CH_2-CH_3), 28.07 (CH_2-CH_2-$\mathit{C}H_2$-CH_2-CH_2-CH_3), 25.68 (CH_2-CH_2-CH_2-$\mathit{C}H_2$-CH_3), 22 (CH_2-CH_2-CH_2-CH_2-$\mathit{C}H_2$-CH_3), 21.70 (CH_2-CH_2-CH_2-CH_2-CH_2-$\mathit{C}H_3$), 13.56 (CH_2-CH_2-CH_2-CH_2-$\mathit{C}H_3$).

1-hexyl-3-pentyl-1,2,3-benzotriazolium tetrafluoroborate(1hex3pentBTBF$_4$)

^{1}H-NMR ($CDCl_3$, 400 MHz): 8.10 (m, 2H, Ar), 7.61 (m, 2H, Ar), 4.79 (m, 4H, $\mathit{CH_2}$-CH_2-CH_2-CH_2-CH_2-CH_3,$\mathit{CH_2}$-CH_2-CH_2-CH_2-CH_3), 1.84 (m, 4H, CH_2-$\mathit{CH_2}$-$\mathit{CH_2}$-CH_2-CH_3), 1.02 (m, 6H, CH_2-$\mathit{CH_2}$-$\mathit{CH_2}$-$\mathit{CH_2}$-CH_2-CH_3), 0.55 (t, 6H, CH_2-CH_2-CH_2-CH_2-$\mathit{CH_3}$, CH_2-CH_2-CH_2-CH_2-CH_2-$\mathit{CH_3}$,). ^{13}C NMR

(CDCl$_3$, 100 MHz): 138.00 (C-8, C-9), 129.83 (C-4, C-7), 112.17 (C-5, C-6), 50.53 (*C*H$_2$-CH$_2$-CH$_2$-CH$_2$-CH$_3$), 29.12 (*C*H$_2$-CH$_2$-CH$_2$-CH$_2$-CH$_2$-CH$_3$,), 27.12 (CH$_2$-*C*H$_2$-CH$_2$-CH$_2$-CH$_3$,), 26.87(CH$_2$-*C*H$_2$-CH$_2$-CH$_2$-CH$_2$-CH$_3$), 26.46 (CH$_2$-CH$_2$-*C*H$_2$-CH$_2$-CH$_2$-CH$_3$), 24.08 (CH$_2$-CH$_2$-CH$_2$-*C*H$_2$-CH$_3$), 20.44 (CH$_2$-CH$_2$-CH$_2$-CH$_2$-*C*H$_2$-CH$_3$), 20.12 (CH$_2$-CH$_2$-CH$_2$-CH$_2$-*C*H$_3$), 11.99 (CH$_2$-CH$_2$-CH$_2$-CH$_2$-CH$_2$-*C*H$_3$).

1-hexyl-3-pentyl-1,2,3-benzotriazolium hexafluorophosphate(1hex3pentBTPF$_6$)

^{1}H-NMR (CDCl$_3$, 400 MHz): 8.06 (m, 2H,Ar), 7.86 (m, 2H, Ar), 4.90 (m, 4H, *CH$_2$*-CH$_2$-CH$_2$-CH$_2$-CH$_2$-CH$_3$, *CH$_2$*-CH$_2$-CH$_2$-CH$_2$-CH$_3$), 2.11 (m, 4H, CH$_2$-*CH$_2$*-*CH$_2$*-CH$_2$-CH$_3$), 1.34 (m, 6H, CH$_2$-*CH$_2$*-*CH$_2$*-*CH$_2$*-CH$_2$-CH$_3$), 0.87 (t, 3H, CH$_2$-CH$_2$-CH$_2$-CH$_2$-*CH$_3$*, CH$_2$-CH$_2$-CH$_2$-CH$_2$-CH$_2$-*CH$_3$*). ^{13}C NMR (CDCl$_3$, 100 MHz): 134.74 (C-8, C-9), 131.60 (C-4, C-7), 113.32 (C-5, C-6), 51.98 (*C*H$_2$-CH$_2$-CH$_2$-CH$_2$-CH$_3$, *C*H$_2$-CH$_2$-CH$_2$-CH$_2$-CH$_2$-CH$_3$), 30.90 (CH$_2$-*C*H$_2$-CH$_2$-CH$_2$-CH$_3$), 28.75 (CH$_2$-*C*H$_2$-CH$_2$-CH$_2$-CH$_2$-CH$_3$), 28.36 (CH$_2$-CH$_2$-*C*H$_2$-CH$_2$-CH$_2$-CH$_3$), 25.93 (CH$_2$-CH$_2$-CH$_2$-*C*H$_2$-CH$_3$), 22.27 (CH$_2$-CH$_2$-CH$_2$-CH$_2$-*C*H$_2$-CH$_3$), 21.91 (CH$_2$-CH$_2$-CH$_2$-CH$_2$-*C*H$_3$), 13.77 (CH$_2$-CH$_2$-CH$_2$-CH$_2$-CH$_2$-*C*H$_3$).

1-butyl-3-pentyl-1,2,3-benzotriazolium bromide(1but3pentBTBr)

^{1}H-NMR (CDCl$_3$, 400 MHz): 8.62 (m, 2H,Ar), 7.96 (m, 2H, Ar), 5.23 (m, 4H, *CH$_2$*-CH$_2$-CH$_2$-CH$_2$-CH$_3$, *CH$_2$*-CH$_2$-CH$_2$-CH$_3$), 2.15 (m, 4H, CH$_2$-*CH$_2$*-*CH$_2$*-CH$_3$,), 1.44 (m, 6H, CH$_2$-*CH$_2$*-*CH$_2$*-*CH$_2$*-CH$_3$), 0.97 (t, 3H,CH$_2$-CH$_2$-CH$_2$-*CH$_3$*,), 0.88 (t, 3H,CH$_2$-CH$_2$-CH$_2$-CH$_2$-*CH$_3$*,). ^{13}C NMR (CDCl$_3$, 100 MHz): 134.57 (C-8, C-9), 131.61 (C-4, C-7), 114.29 (C-5, C-6), 52.66 (*C*H$_2$-CH$_2$-CH$_2$-CH$_3$), 52.43 (*C*H$_2$-CH$_2$-CH$_2$-CH$_2$-CH$_3$,), 30.89 (CH$_2$-*C*H$_2$-CH$_2$-CH$_3$,), 28.64 (CH$_2$-*C*H$_2$-CH$_2$-CH$_2$-CH$_3$), 28.09 (CH$_2$-CH$_2$-*C*H$_2$-CH$_2$-CH$_3$), 21.71 (CH$_2$-CH$_2$-*C*H$_2$-CH$_3$), 19.38 (CH$_2$-CH$_2$-CH$_2$-*C*H$_2$-CH$_3$), 13.53 (CH$_2$-CH$_2$-CH$_2$-CH$_2$-*C*H$_3$), 13.20 (CH$_2$-CH$_2$-CH$_2$-CH$_2$-*C*H$_3$).

1-butyl-3-pentyl-1,2,3-benzotriazolium tetrafluoroborate(1but3pentBTBF$_4$)

^{1}H-NMR ($CDCl_3$, 400 MHz): 8.41 (m, 2H,Ar), 7.93 (m, 2H, Ar), 5.10 (m, 4H, CH_2-CH_2-CH_2-CH_2-CH_3, CH_2-CH_2-CH_2-CH_3), 2.14 (m, 4H, CH_2-CH_2-CH_2-CH_3), 1.42 (m, 6H, CH_2-CH_2-CH_2-CH_2-CH_3), 0.98 (t, 3H,CH_2-CH_2-CH_2-CH_3,), 0.89 (t, 3H,CH_2-CH_2-CH_2-CH_2-CH_3,). ^{13}C NMR ($CDCl_3$, 100 MHz): 132.87 (C-8, C-9), 129.86 (C-4, C-7), 112.16 (C-5, C-6), 50.57 (CH_2-CH_2-CH_2-CH_3), 50.34 (CH_2-CH_2-CH_2-CH_2-CH_3,), 29.05 (CH_2-CH_2-CH_2-CH_3,), 26.82 (CH_2-CH_2-CH_2-CH_2-CH_3), 26.39 (CH_2-CH_2-CH_2-CH_2-CH_3), 20.01 (CH_2-CH_2 -CH_2-CH_3), 17.67 (CH_2-CH_2-CH_2-CH_2-CH_3), 11.81 (CH_2-CH_2-CH_2-CH_2-CH_3), 11.44 (CH_2-CH_2-CH_2-CH_2-CH_3).

1-butyl-3-pentyl-1,2,3-benzotriazolium hexafluorophosphate(1but3pentBTPF$_6$)

^{1}H-NMR ($CDCl_3$, 400 MHz): 8.11 (m, 2H,Ar), 7.87 (m, 2H, Ar), 4.92 (m, 4H, CH_2-CH_2-CH_2-CH_2-CH_3, CH_2-CH_2-CH_2-CH_3), 2.10 (m, 4H, CH_2-CH_2-CH_2-CH_3), 1.40 (m, 6H , CH_2-CH_2-CH_2-CH_2-CH_3), 0.97 (t, 3H, CH_2-CH_2-CH_2-CH_3), 0.89 (t, 3H, CH_2-CH_2-CH_2-CH_2-CH_3,). ^{13}C NMR ($CDCl_3$, 100 MHz): 134.74 (C-8, C-9), 131.69 (C-4, C-7), 113.50 (C-5, C-6), 52.07 (CH_2-CH_2-CH_2-CH_3), 51.83 (CH_2-CH_2-CH_2-CH_2-CH_3,), 30.76 (CH_2-CH_2-CH_2-CH_3,), 28.54 (CH_2-CH_2-CH_2-CH_2-CH_3), 28.31 (CH_2-CH_2-CH_2-CH_2-CH_3), 21.91 (CH_2-CH_2-CH_2-CH_3), 19.58 (CH_2-CH_2-CH_2-CH_2-CH_3), 13.69 (CH_2-CH_2-CH_2-CH_2-CH_3), 10.28 (CH_2-CH_2-CH_2-CH_2-CH_3).

1-pentyl-3-propyl-1,2,3-benzotriazolium bromide(1pent3propBTBr)

^{1}H-NMR ($CDCl_3$, 400 MHz): 8.62(m, 2H, Ar), 7.97 (m, 2H, Ar), 5.22 (t, 4H, CH_2-CH_2-CH_2-CH_2-CH_3, CH_2-CH_2-CH_3), 2.22 (m, 4H, CH_2-CH_2-CH_2-CH_2-CH_3,), 1.40 (CH_2-CH_2-CH_3, CH_2-CH_2-CH_2-CH_2-CH_3),1.06 (t, 3H, CH_2-CH_2-CH_3,), 0.88 (t, 3H, CH_2-CH_2-CH_2-CH_2-CH_3,). ^{13}C NMR ($CDCl_3$, 100 MHz): 134.55 (C-8, C-9), 131.56 (C-4, C-7), 114.20 (C-5, C-6), 53.97 (CH_2-CH_2-CH_3), 52.57 (CH_2-CH_2-CH_2-CH_2-CH_3,), 28.57 (CH_2-CH_2-CH_2-CH_2-CH_3,), 28.04 (CH_2-CH_2-CH_2-CH_2-CH_3), 22.55 (CH_2-CH_2-CH_2-CH_2-CH_3), 21.67 (CH_2-CH_2-CH_3), 13.49 (CH_2-CH_2-CH_3), 10.75 (CH_2-CH_2-CH_2-CH_2-CH_3).

1-pentyl-3-propyl-1,2,3-benzotriazolium tetrafluoroborate(1pent3propBTBF$_4$)

^{1}H-NMR ($CDCl_3$, 400 MHz): 7.92 (m, 2H,Ar), 7.41 (m, 2H, Ar), 4.57 (m, 4H, *CH2*-CH2-CH2-CH2-CH3,*CH2*-CH2-CH3), 1.64 (m, 4H, CH2-*CH2*-*CH2*-CH2-CH3), 0.86 (m, 4H, CH2-*CH2*-CH3, CH2-CH2-CH2-*CH2*-CH3), 0.50 (t, 3H, CH2-CH2-*CH3,)*, 0.34 (t, 3H, CH2-CH2-CH2-CH2-*CH3,)*. ^{13}C NMR ($CDCl_3$, 100 MHz): 134.57 (C-8, C-9), 131.53 (C-4, C-7), 113.90 (C-5, C-6), 53.65 (*C*H2-CH2-CH3), 52.24 (*C*H2-CH2-CH2-CH2-CH3,), 28.48 (CH2-*C*H2-CH2-CH2-CH3,), 28.04 (CH2-CH2-*C*H2-CH2-CH3), 22.45 (CH2-CH2-CH2-*C*H2-CH3), 21.68 (CH2-*C*H2-CH3), 13.51 (CH2-CH2-*C*H3), 10.70 (CH2-CH2-CH2-CH2-*C*H3).

1-pentyl-3-propyl-1,2,3-benzotriazolium hexafluorophosphate(1pent3propBTPF$_6$)

^{1}H-NMR ($CDCl_3$, 400 MHz): 8.09 (m, 2H, Ar), 7.87 (m, 2H, Ar), 4.89 (m, 4H, *CH2*-CH2-CH2-CH2-CH3, *CH2*-CH2-CH3), 2.14 (m, 4H, CH2-*CH2*-*CH2*-CH2-CH3), 1.37 (m, 4H, CH2-*CH2*-CH3, CH2-CH2-CH2-*CH2*-CH3),1.02 (t, 3H, CH2-CH2-*CH3*), 0.88 (t, 3H, CH2-CH2-CH2-CH2-*CH3,)*. ^{13}C NMR ($CDCl_3$, 100 MHz): 134.76 (C-8, C-9), 131.66 (C-4, C-7), 113.45 (C-5, C-6), 53.44 (*C*H2-CH2-CH3), 52.01 (*C*H2-CH2-CH2-CH2-CH3,), 28.52 (CH2-*C*H2-CH2-CH2-CH3,), 28.30 (CH2-CH2-*C*H2-CH2-CH3), 22.46 (CH2-CH2-CH2-*C*H2-CH3), 21.91 (CH2-*C*H2-CH3), 13.68 (CH2-CH2-*C*H3), 10.82 (CH2-CH2-CH2-CH2-*C*H3).

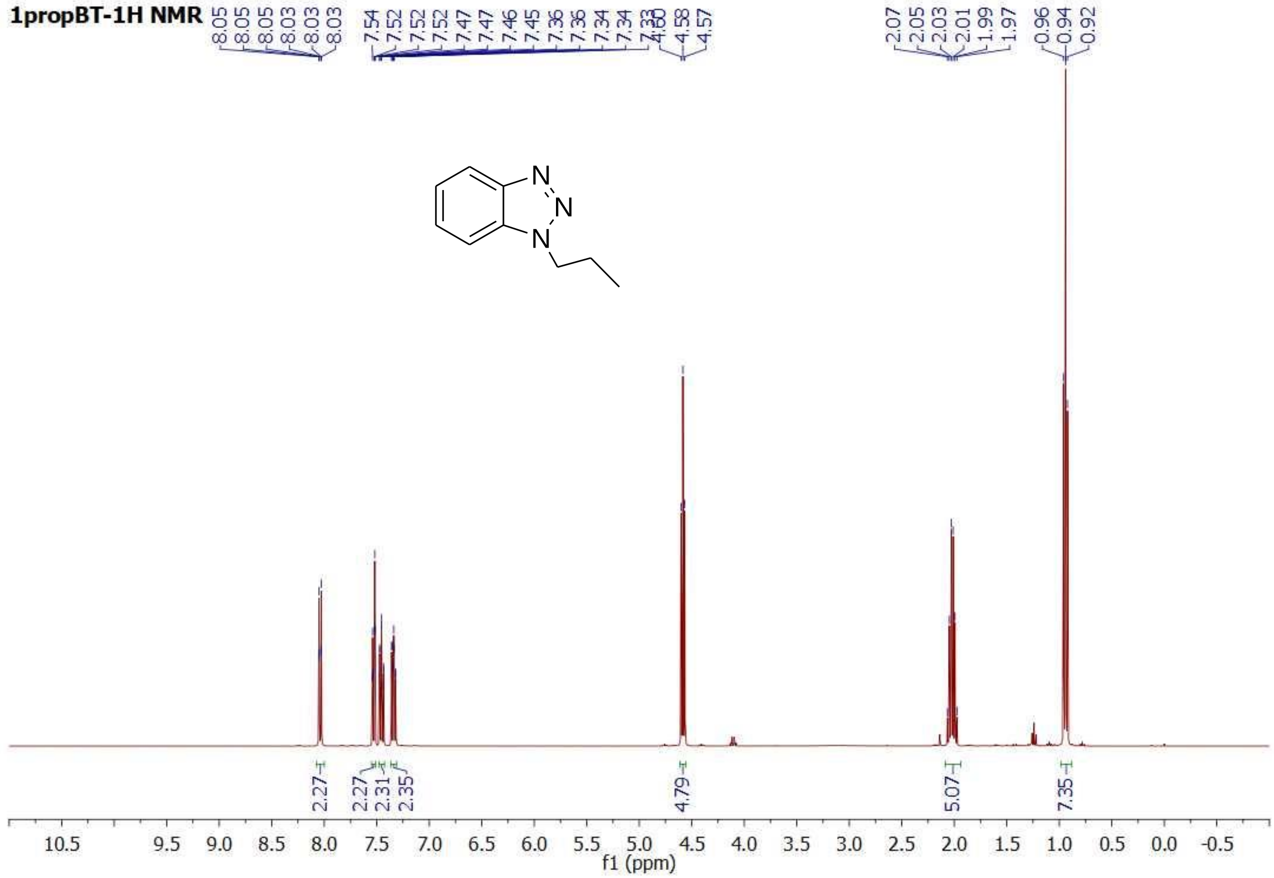

Figure S2. 1propBT-^{1}H NMR

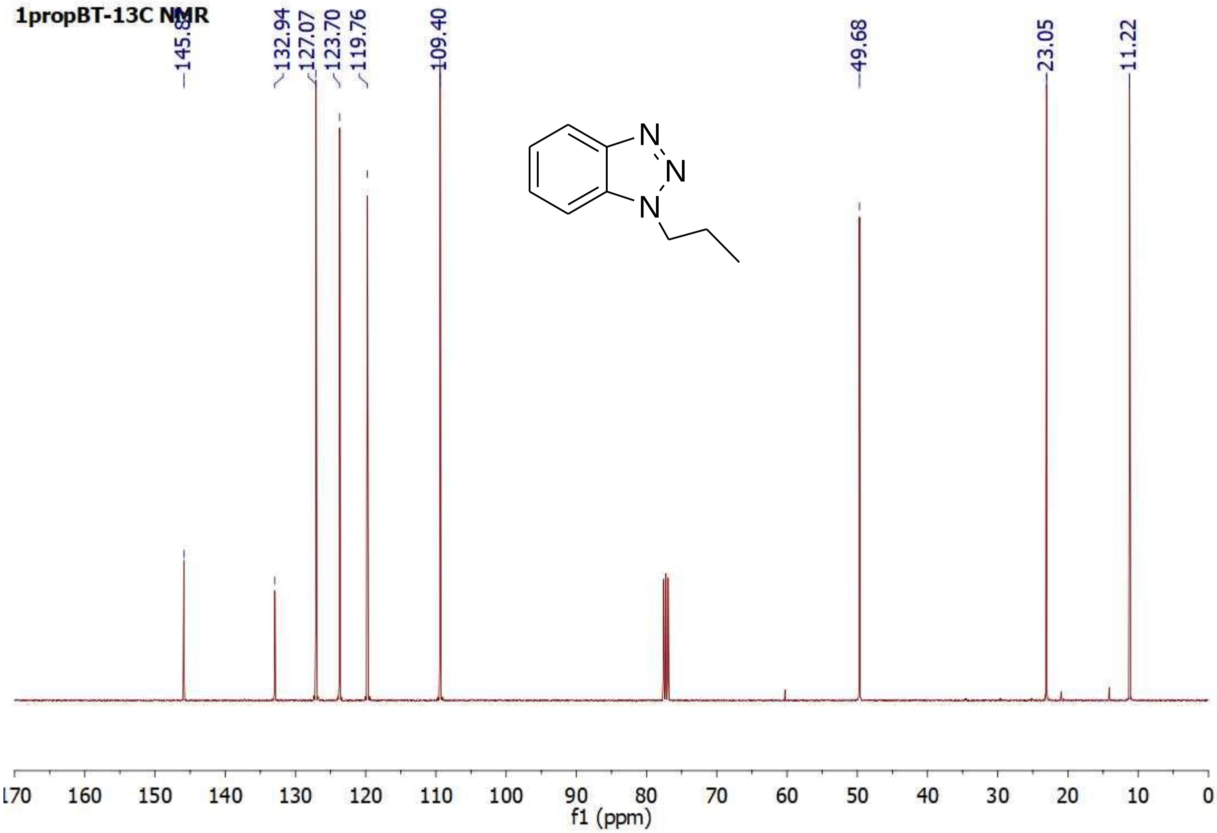

Figure S3. 1propBT-^{13}C NMR

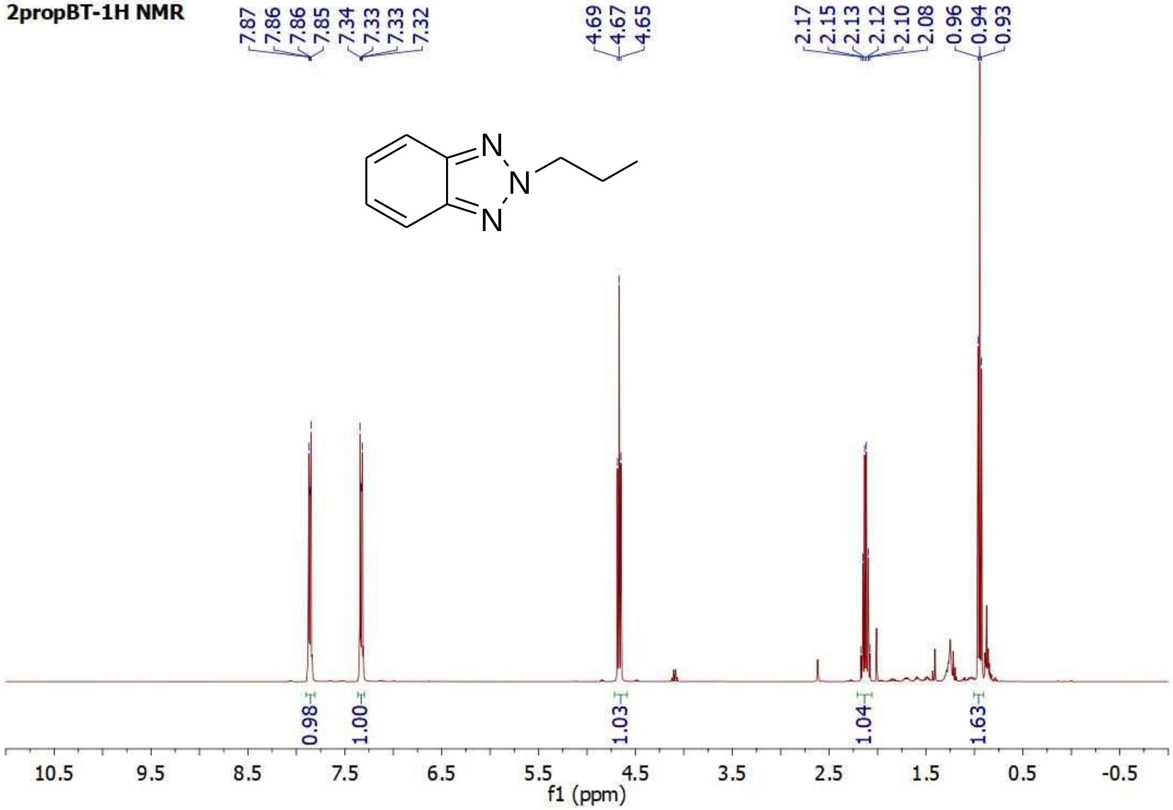

Figure S4. 2propBT-^{1}H NMR

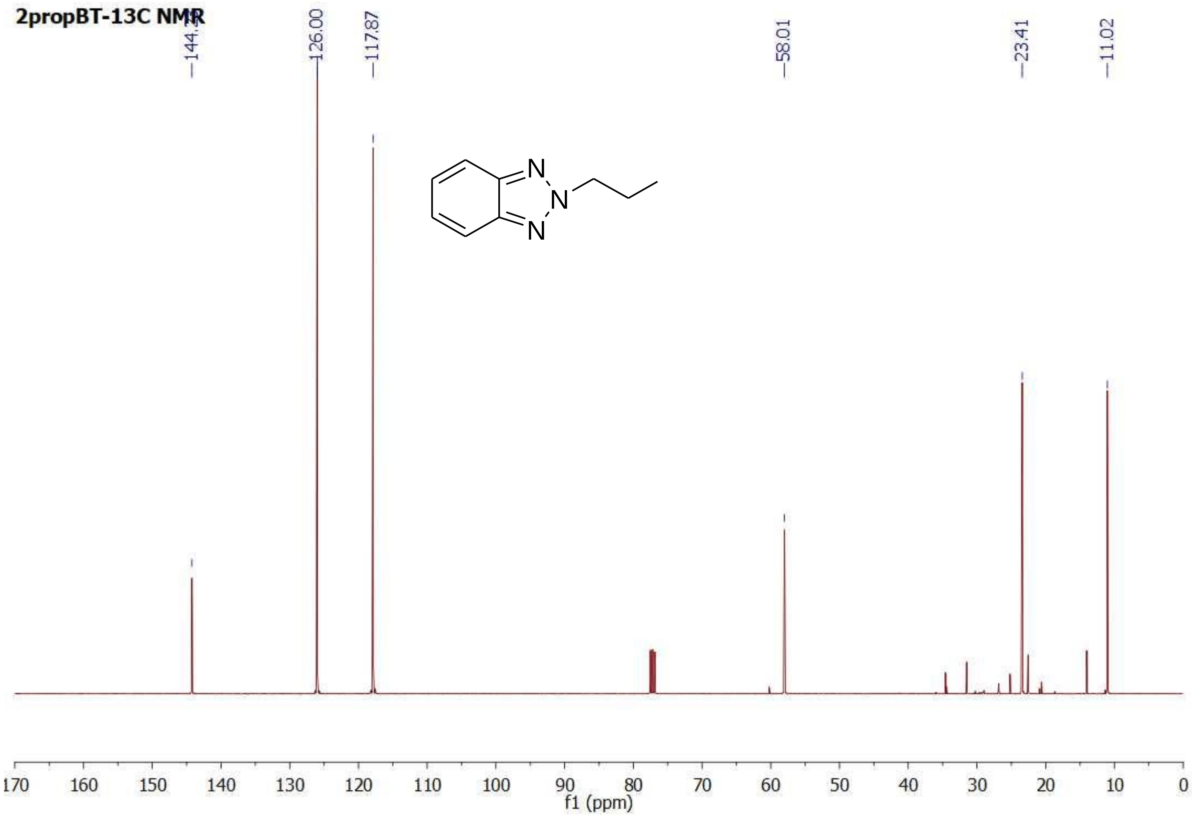

Figure S5. 2propBT-^{13}C NMR

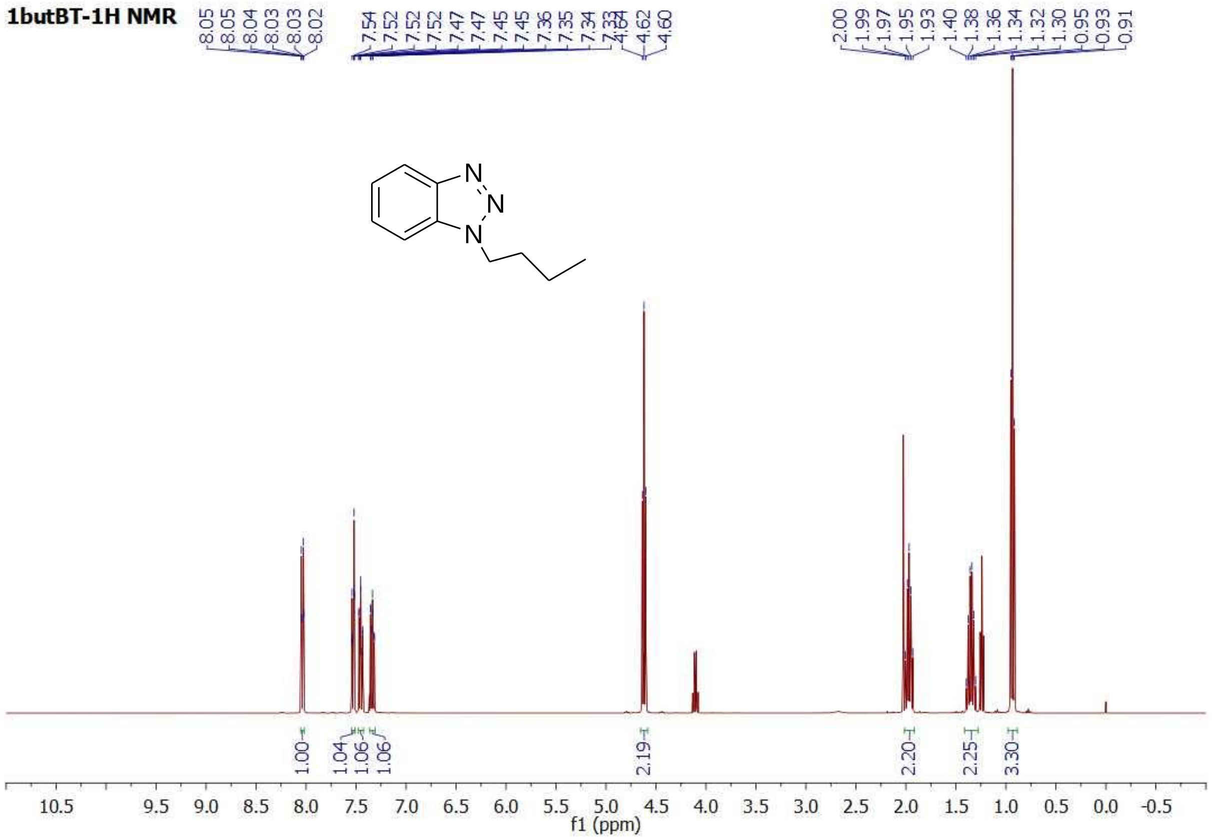

Figure S6. 1butBT-^{1}H NMR

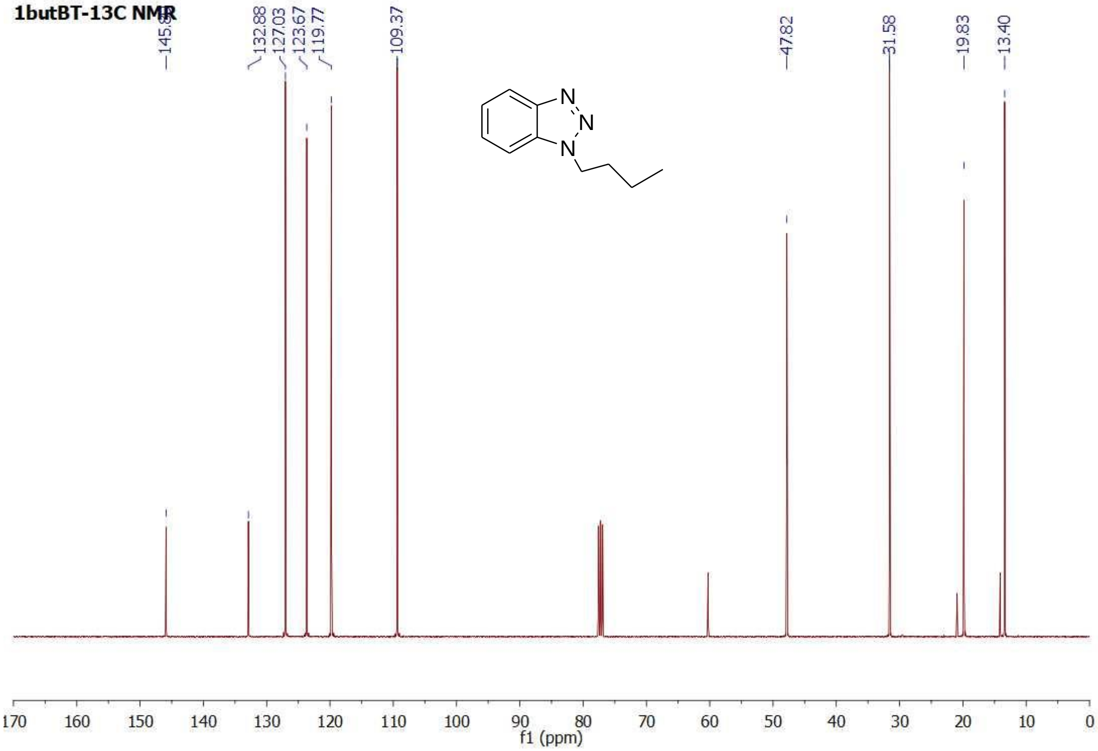

Figure S7. 1butBT-^{13}C NMR

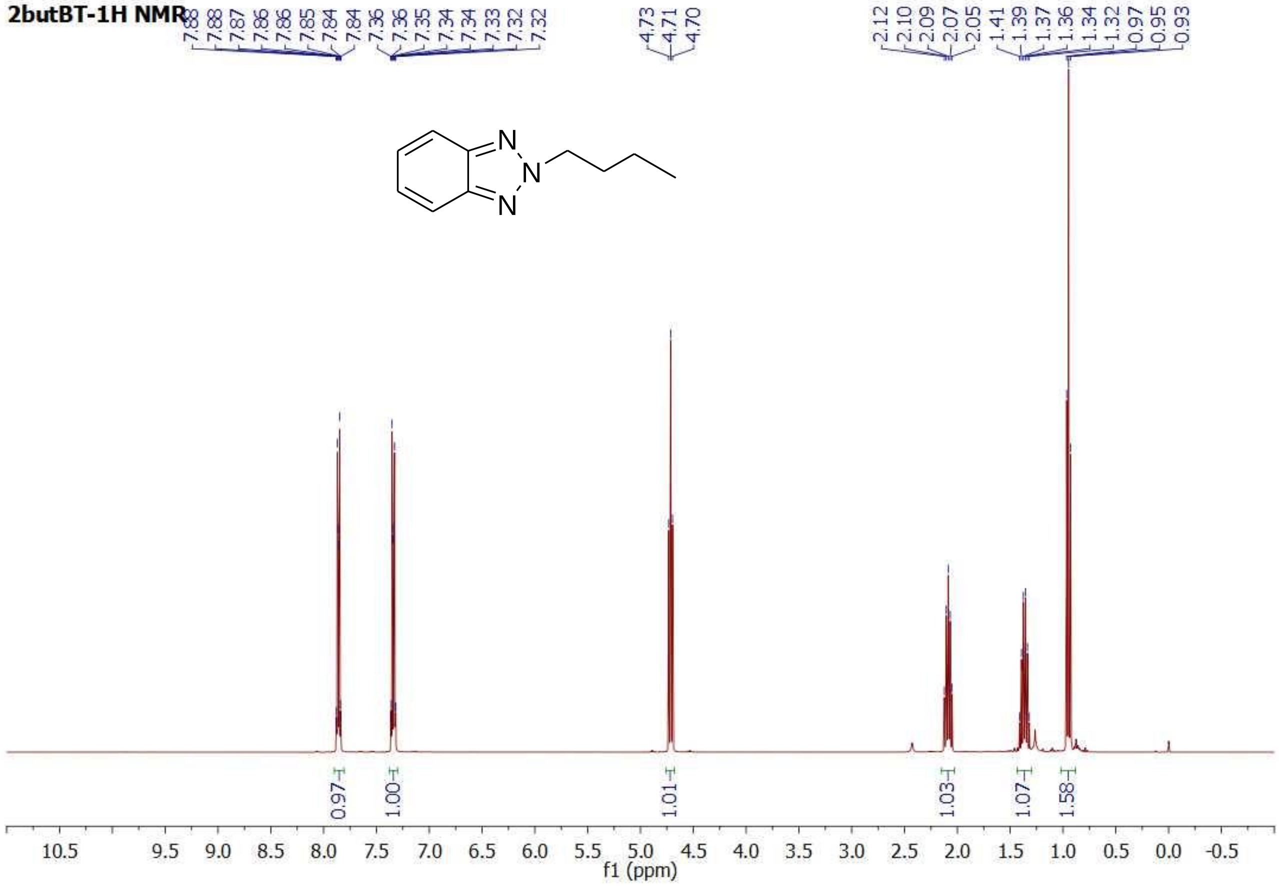

Figure S8.2butBT-^{1}H NMR

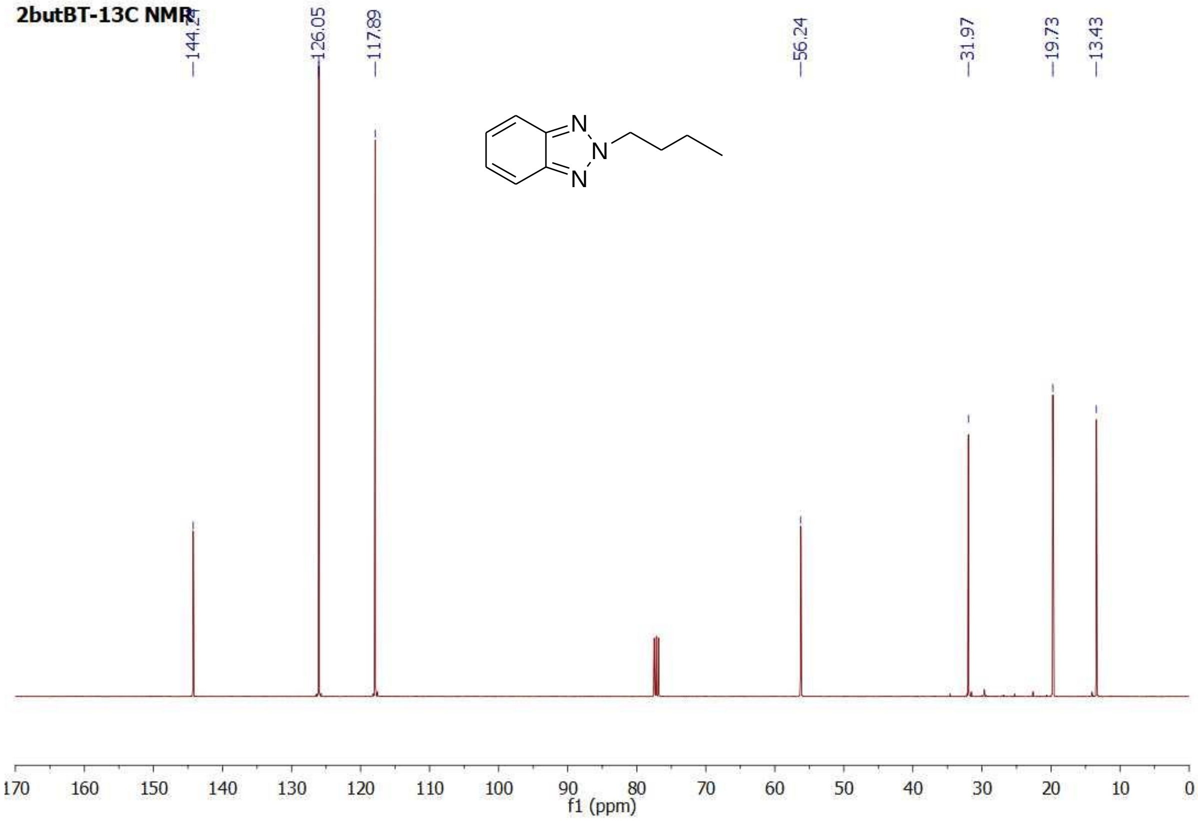

Figure S9.2butBT-^{13}C NMR

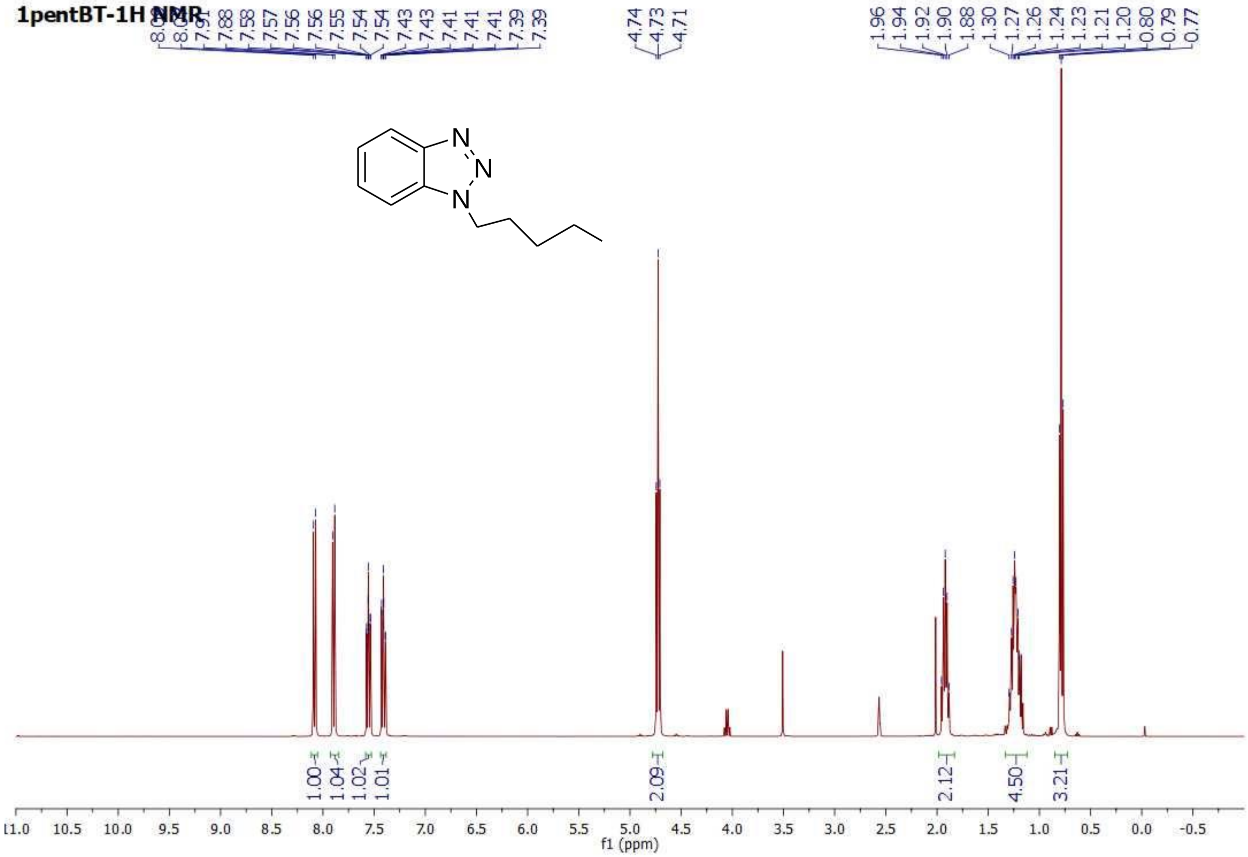

Figure S10.1pentBT-^{1}H NMR

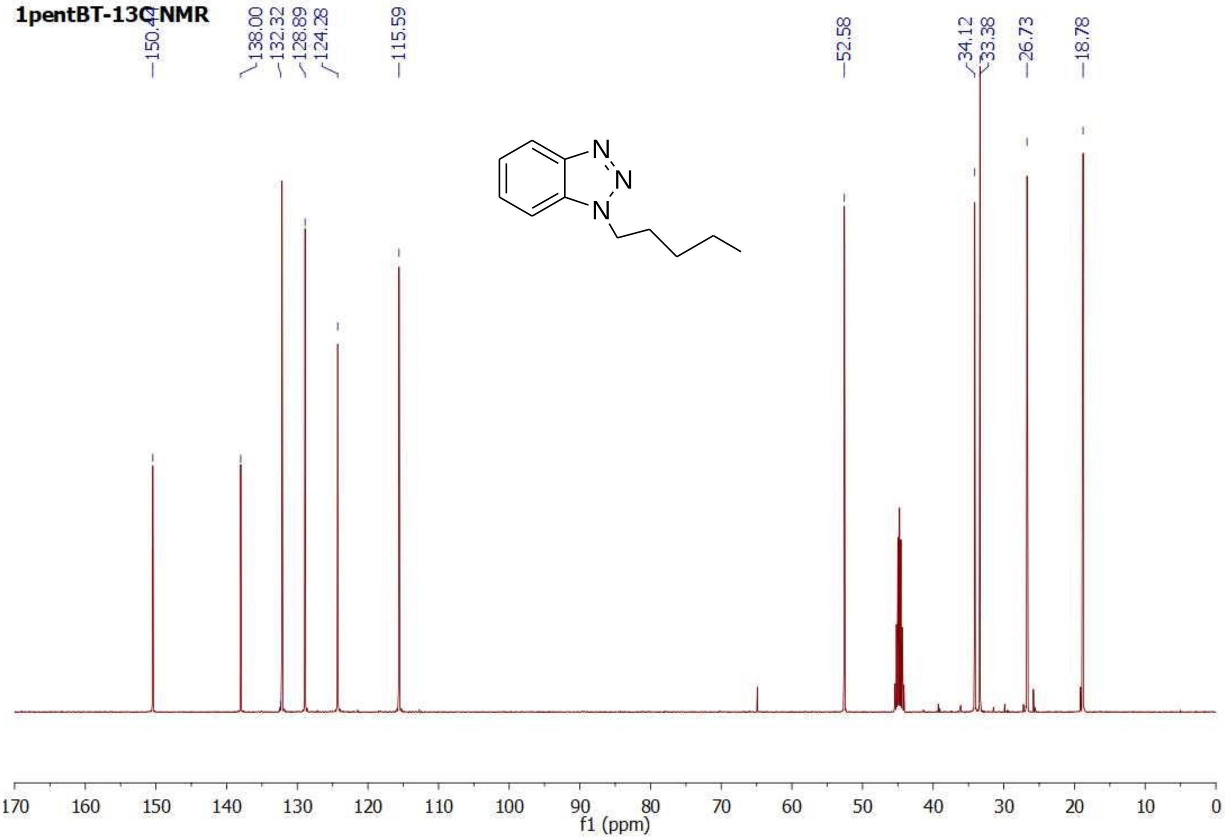

Figure S11.1pentBT-^{13}C NMR

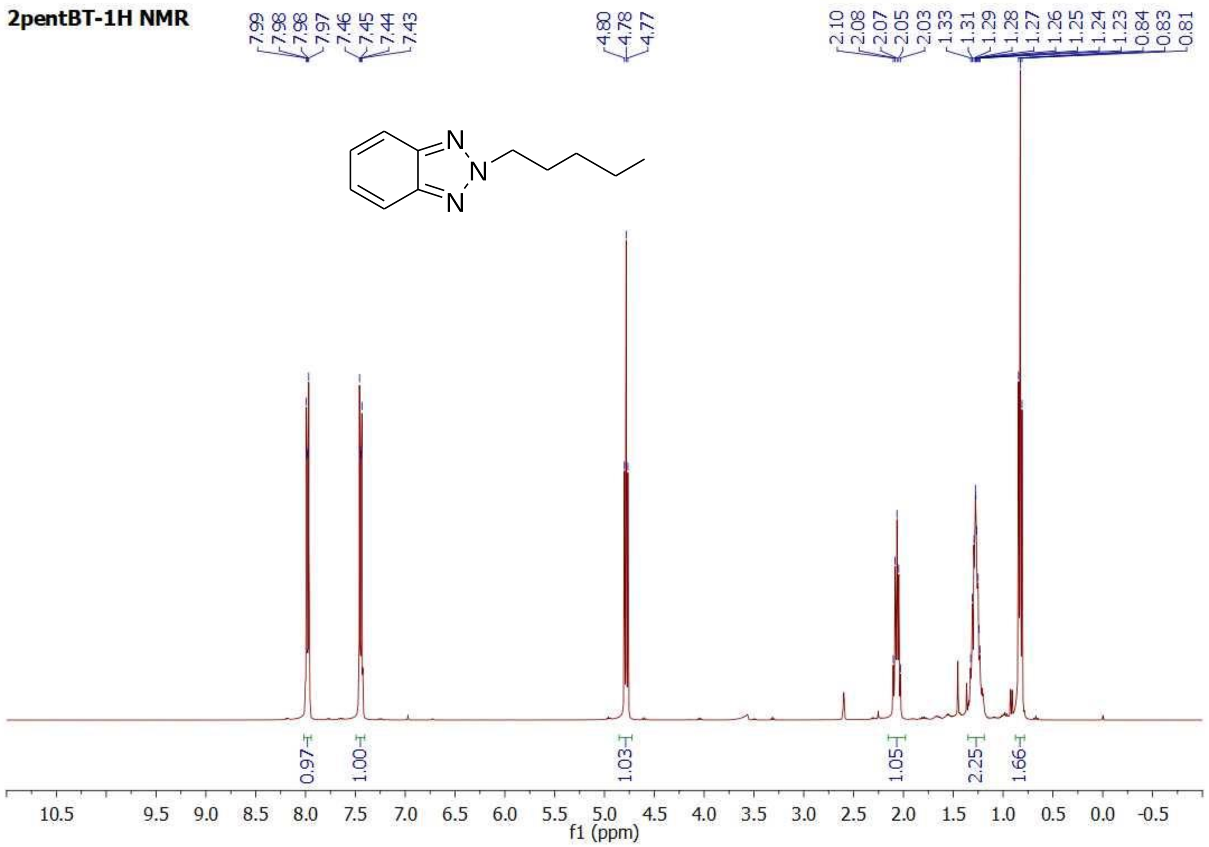

Figure S12. 2pentBT-^{1}H NMR

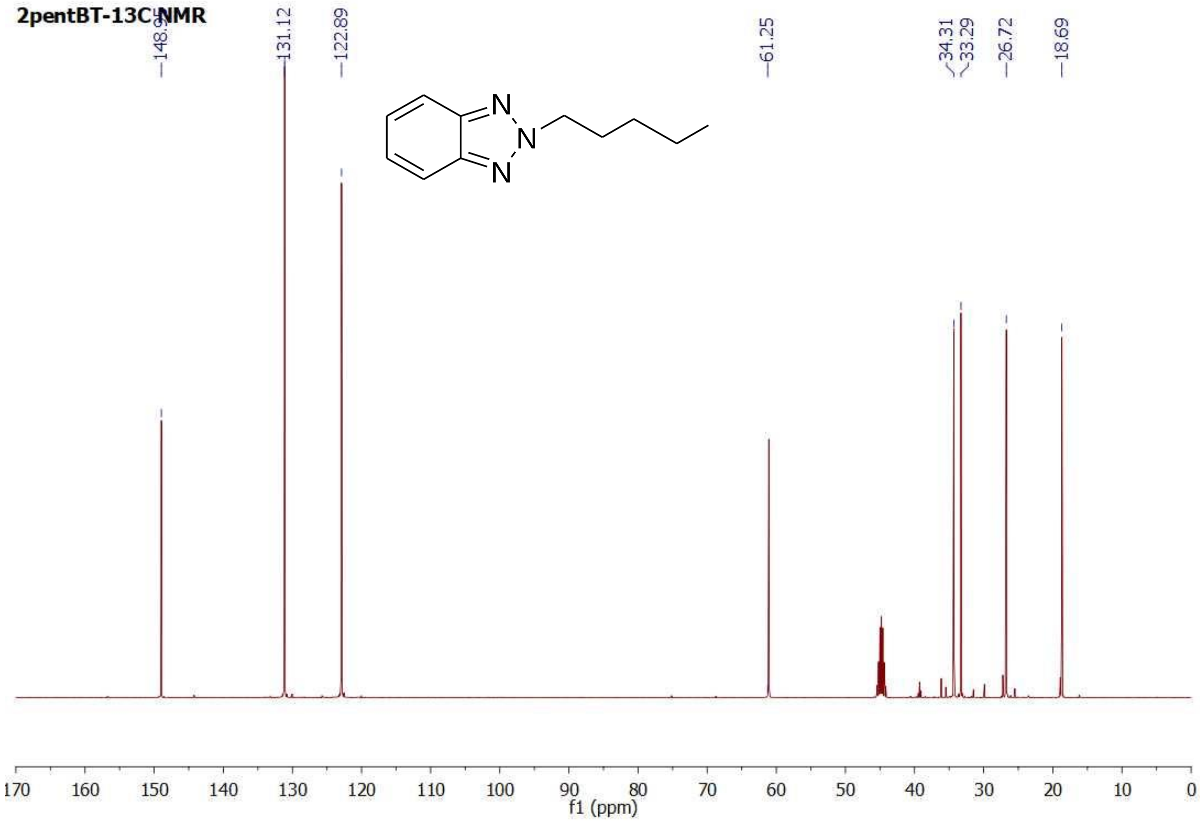

Figure S13. 2pentBT-^{13}C NMR

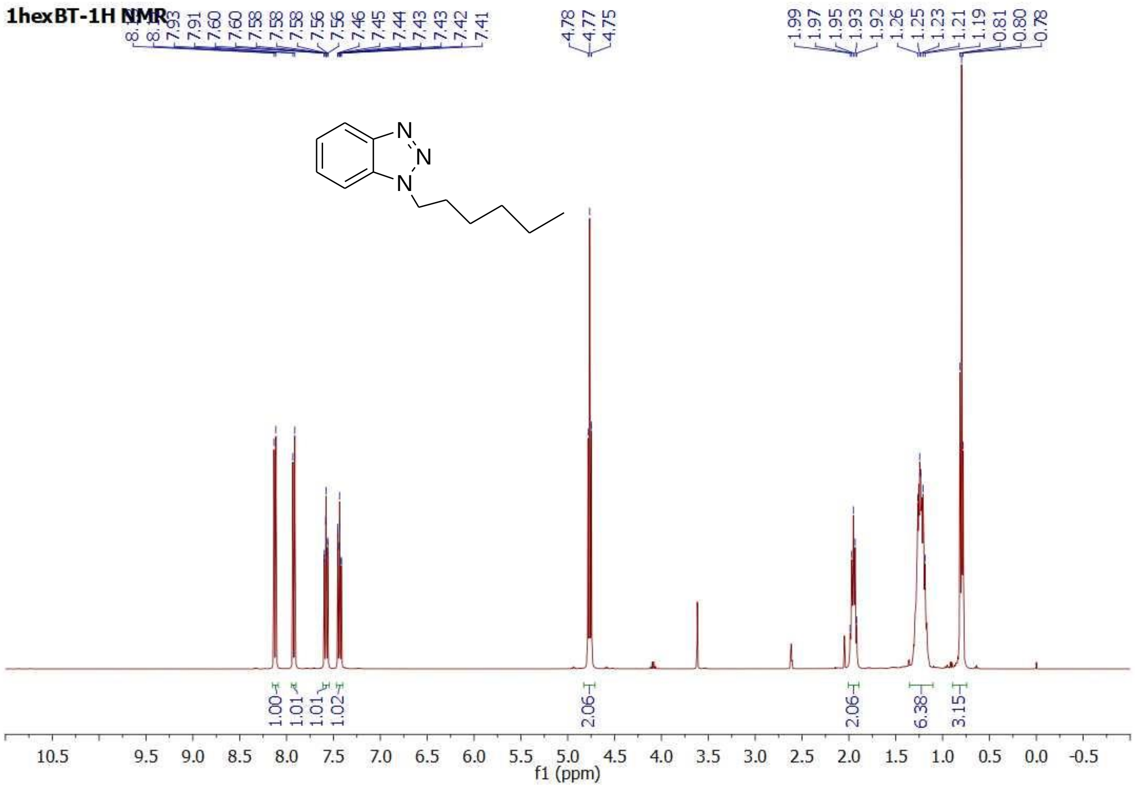

Figure S14.1hexBT-^{1}H NMR

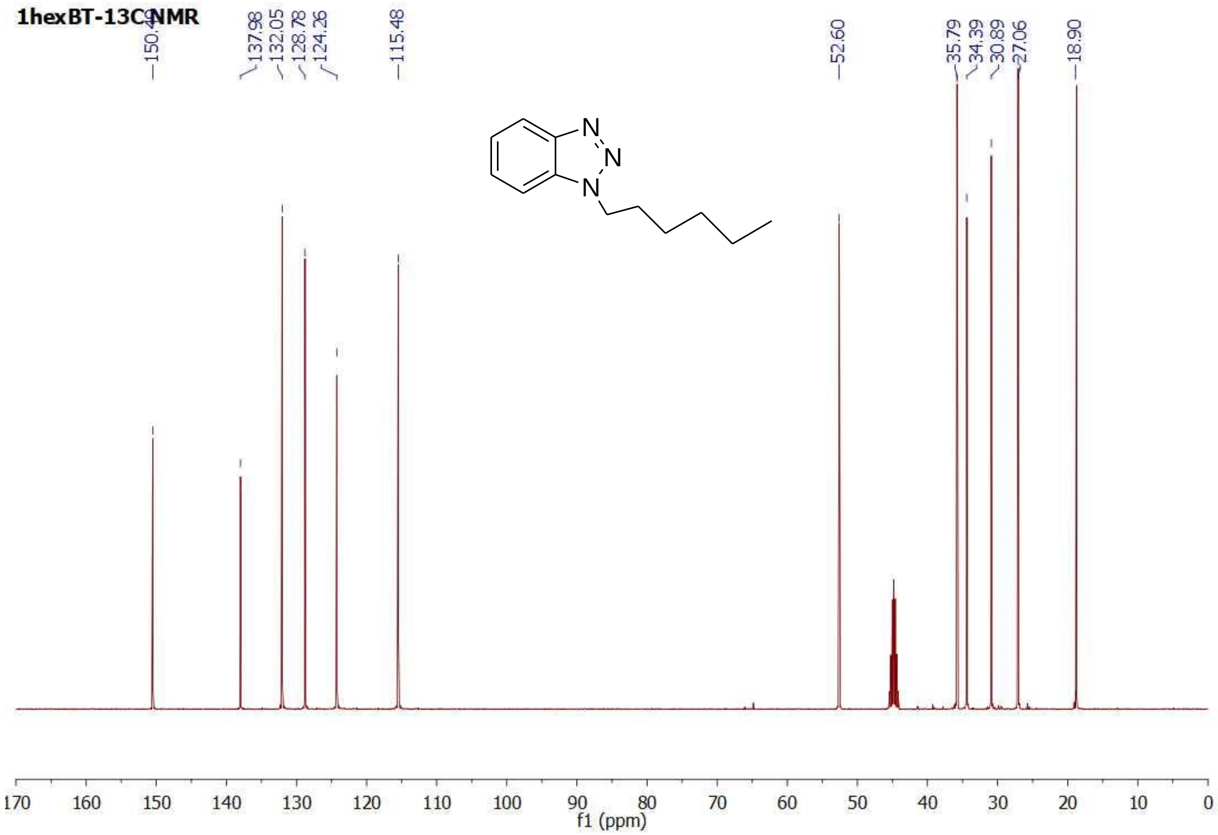

Figure S15.1hexBT-^{13}C NMR

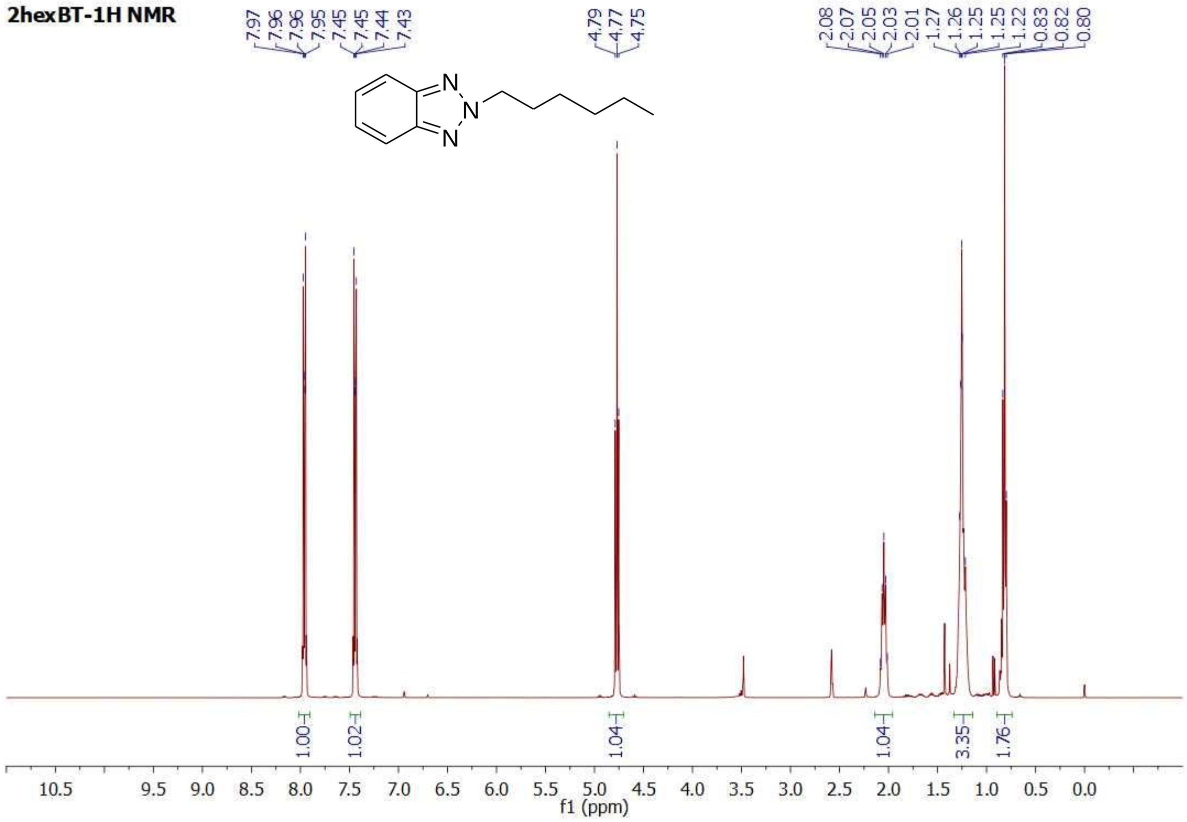

Figure S16. 2hexBT-^{1}H NMR

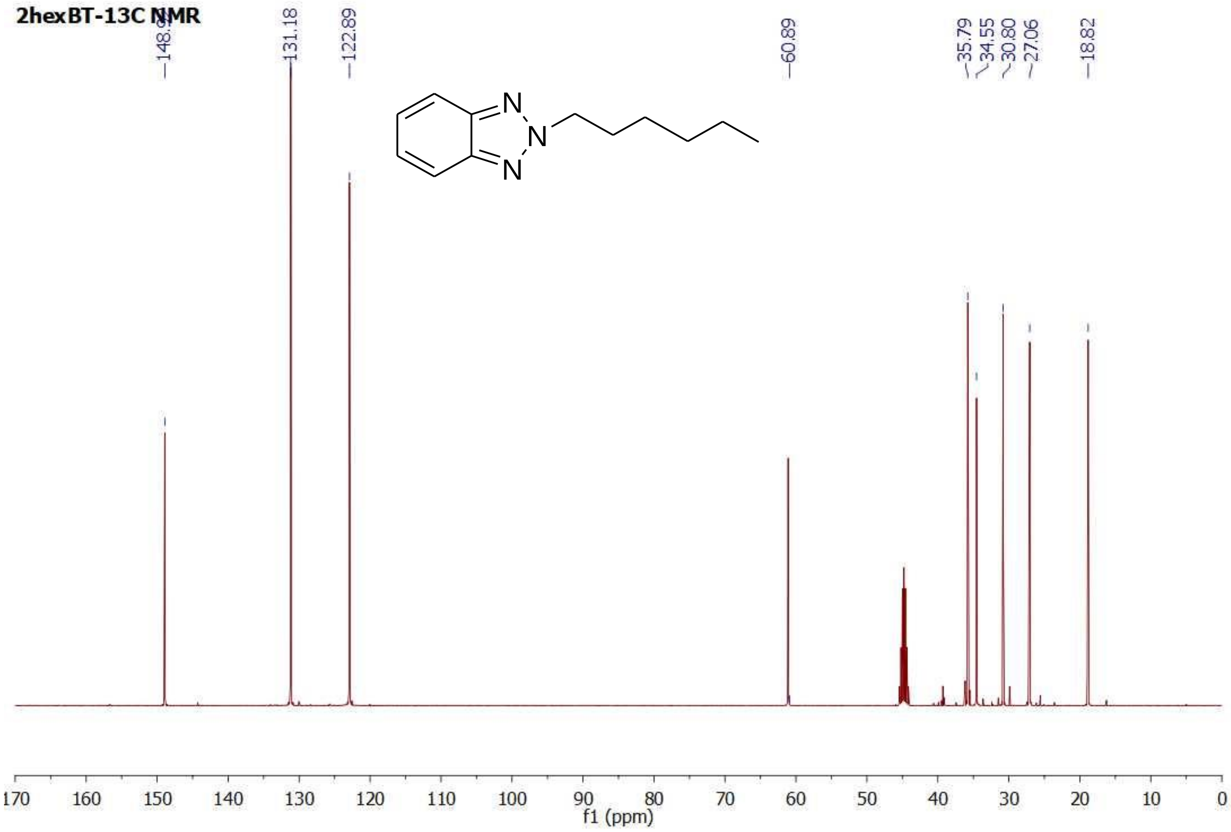

Figure S17. 2hexBT-^{13}C NMR

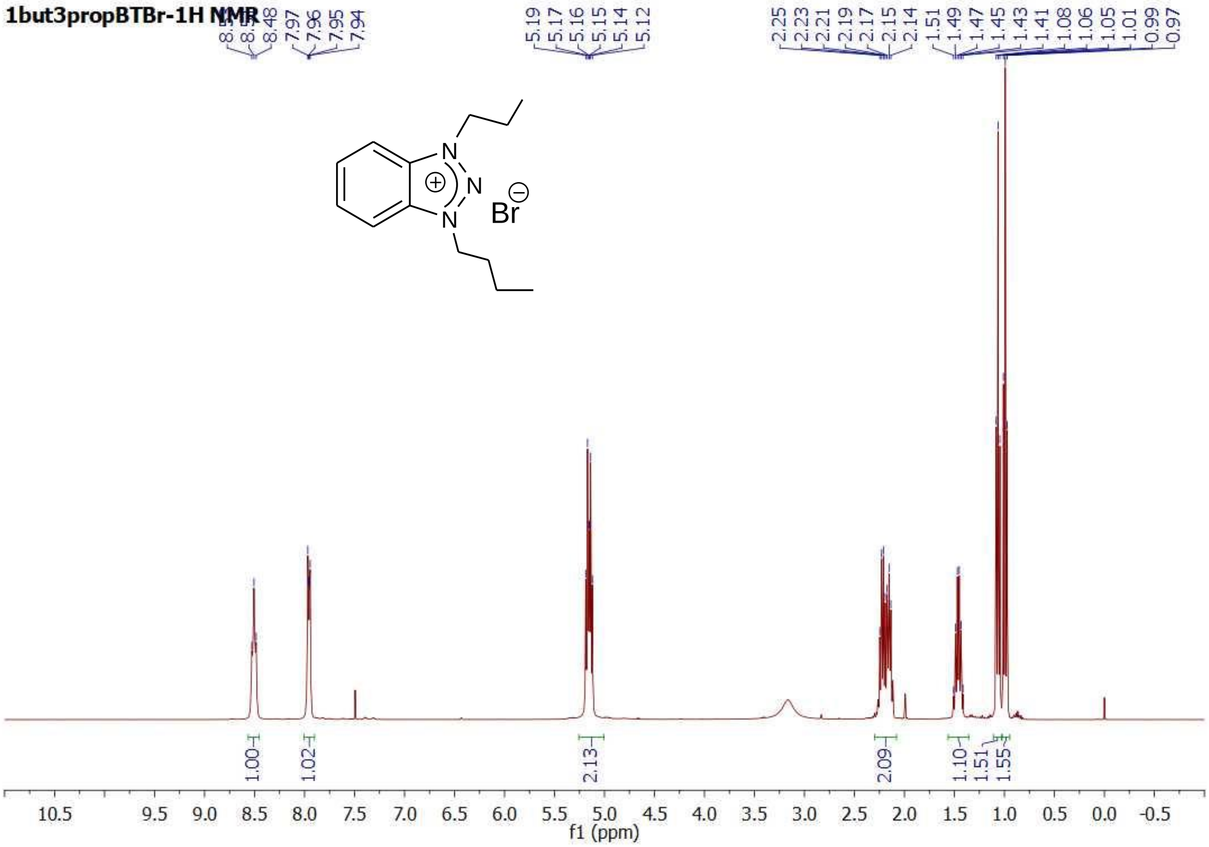

Figure S18. 1but3propBTBr-^{1}H NMR

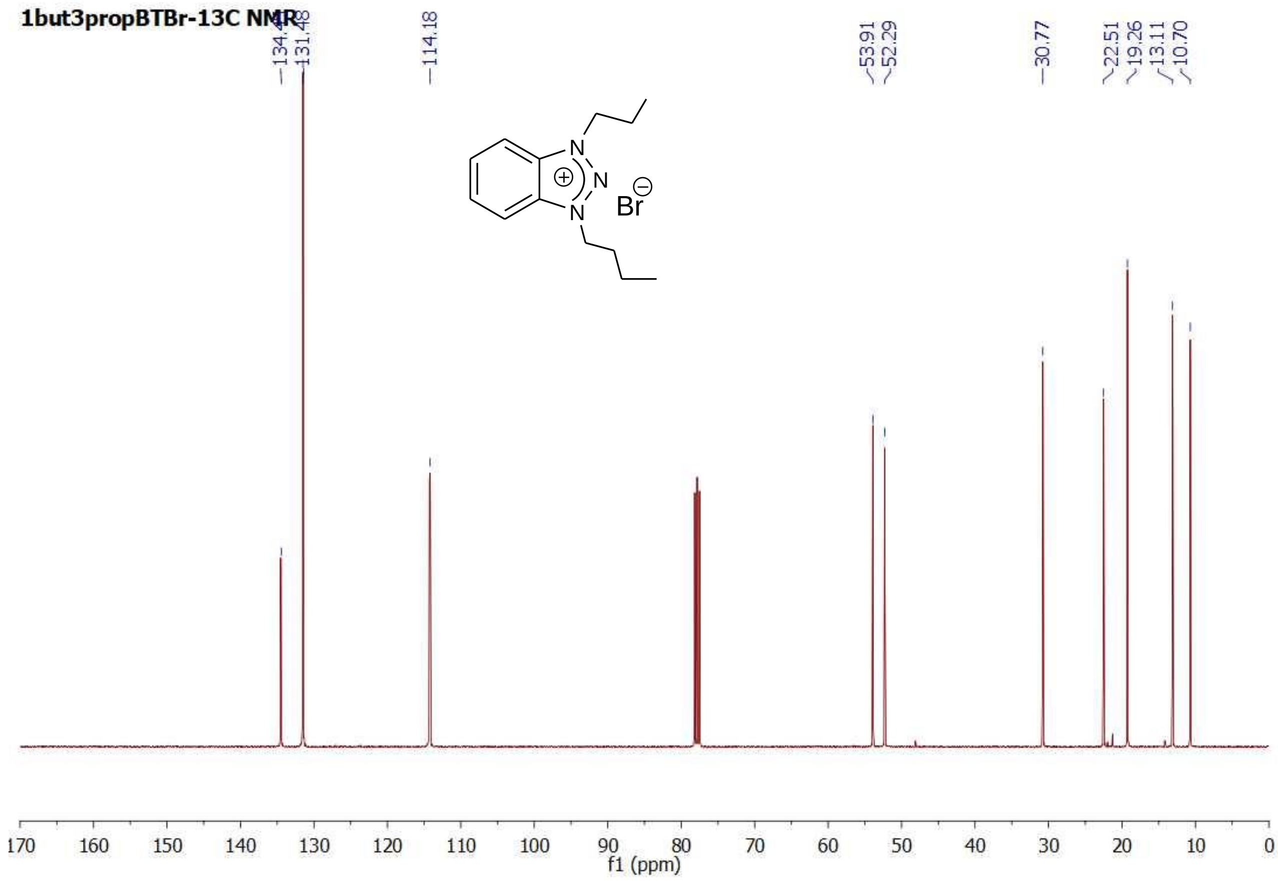

Figure S19. 1but3propBTBr-^{13}C NMR

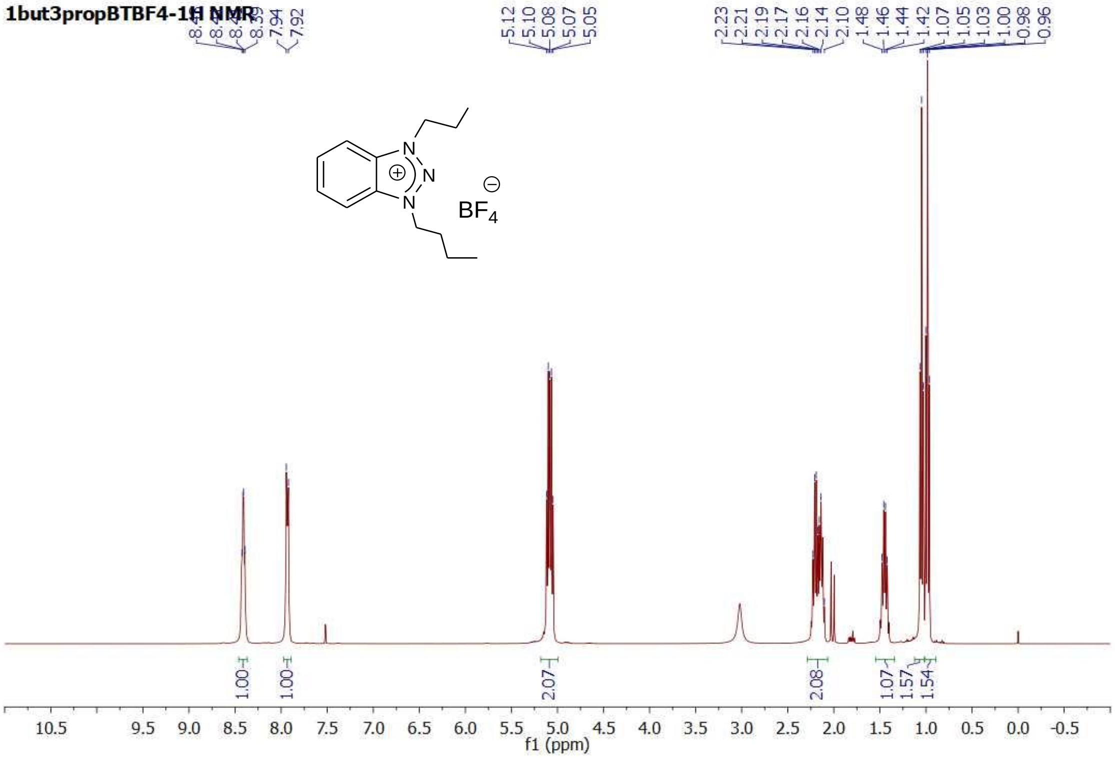

Figure S20. 1but3propBTBF$_4$-^{1}H NMR

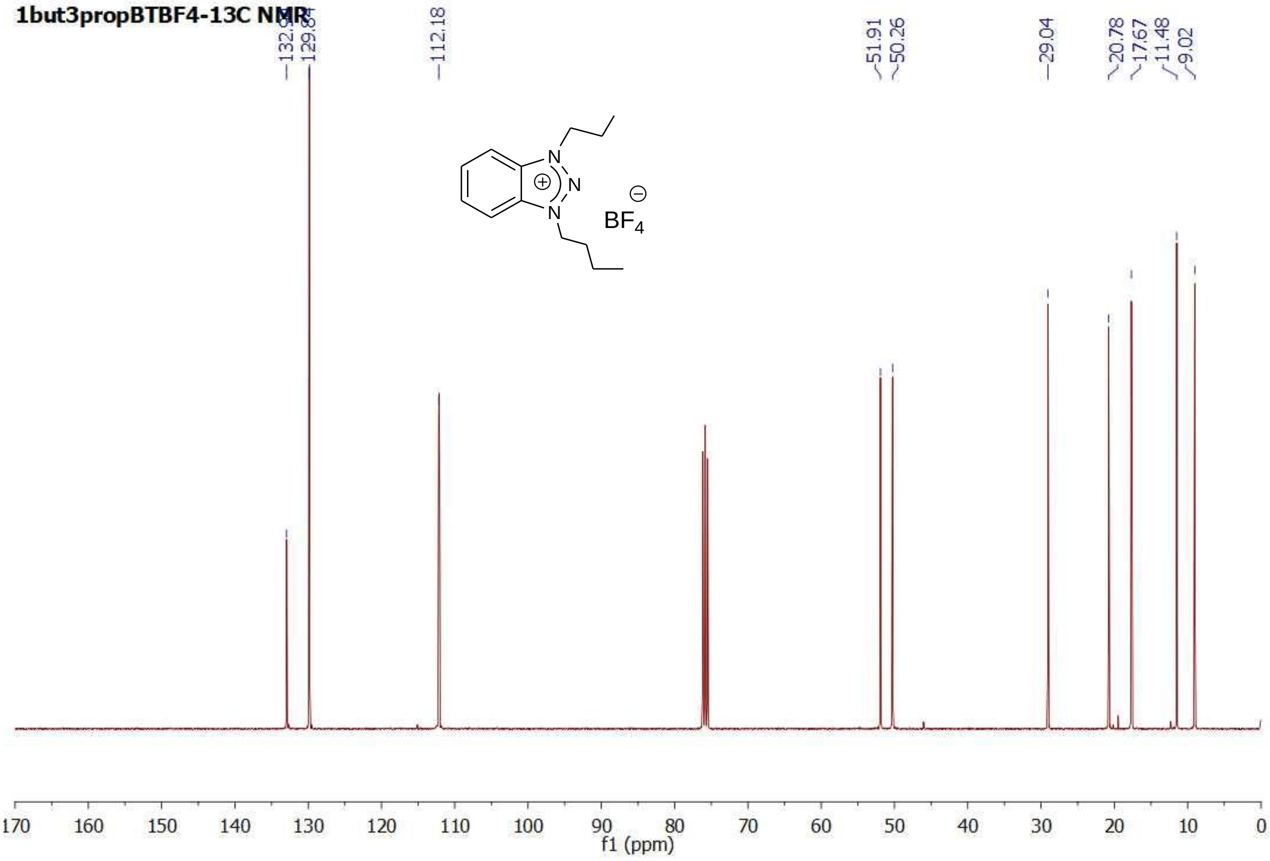

Figure S21. 1but3propBTBF_4-^{13}C NMR

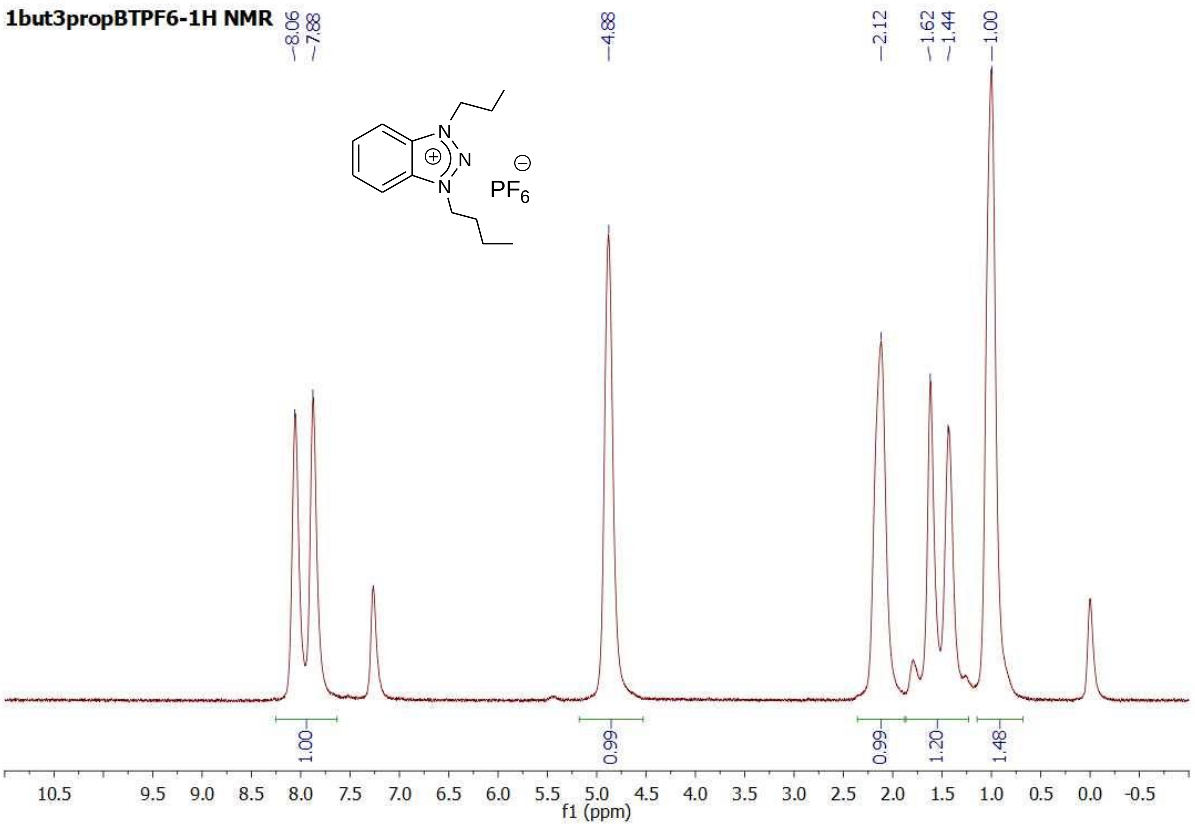

Figure S22. 1but3propBTPF$_6$-^{1}H NMR

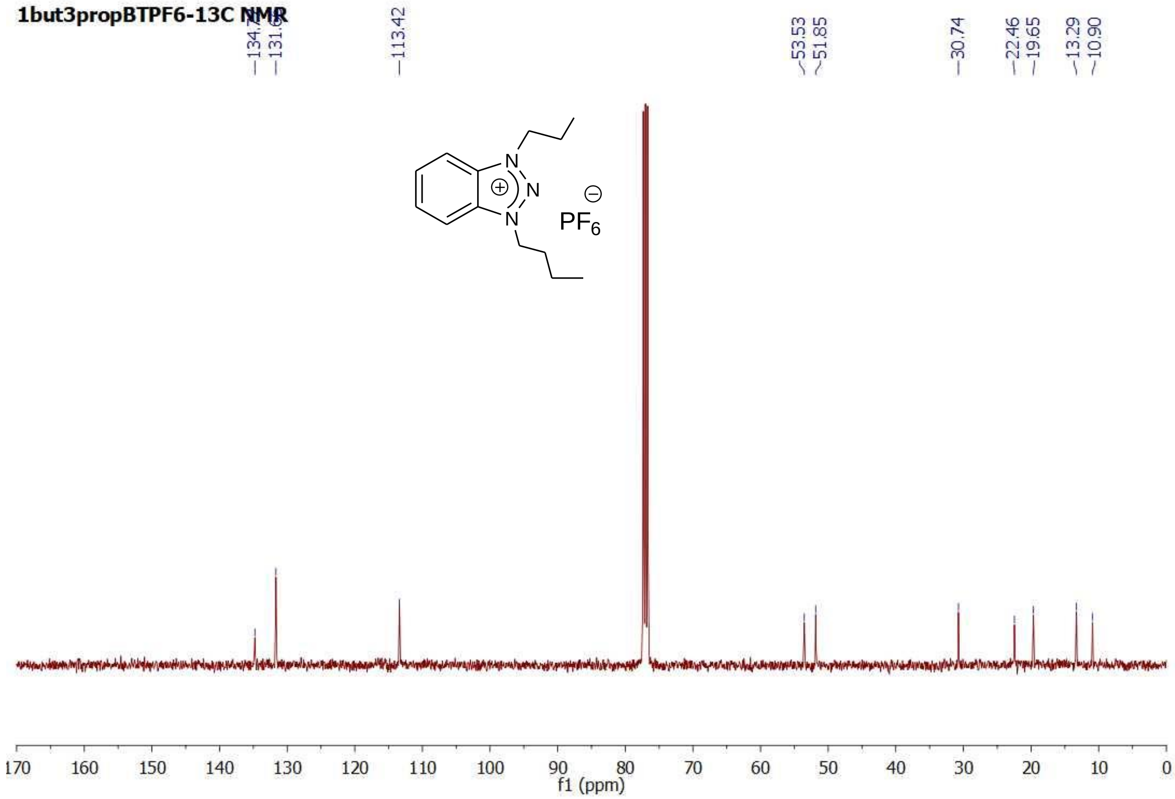

Figure S23. 1but3propBTPF$_6$-^{13}C NMR

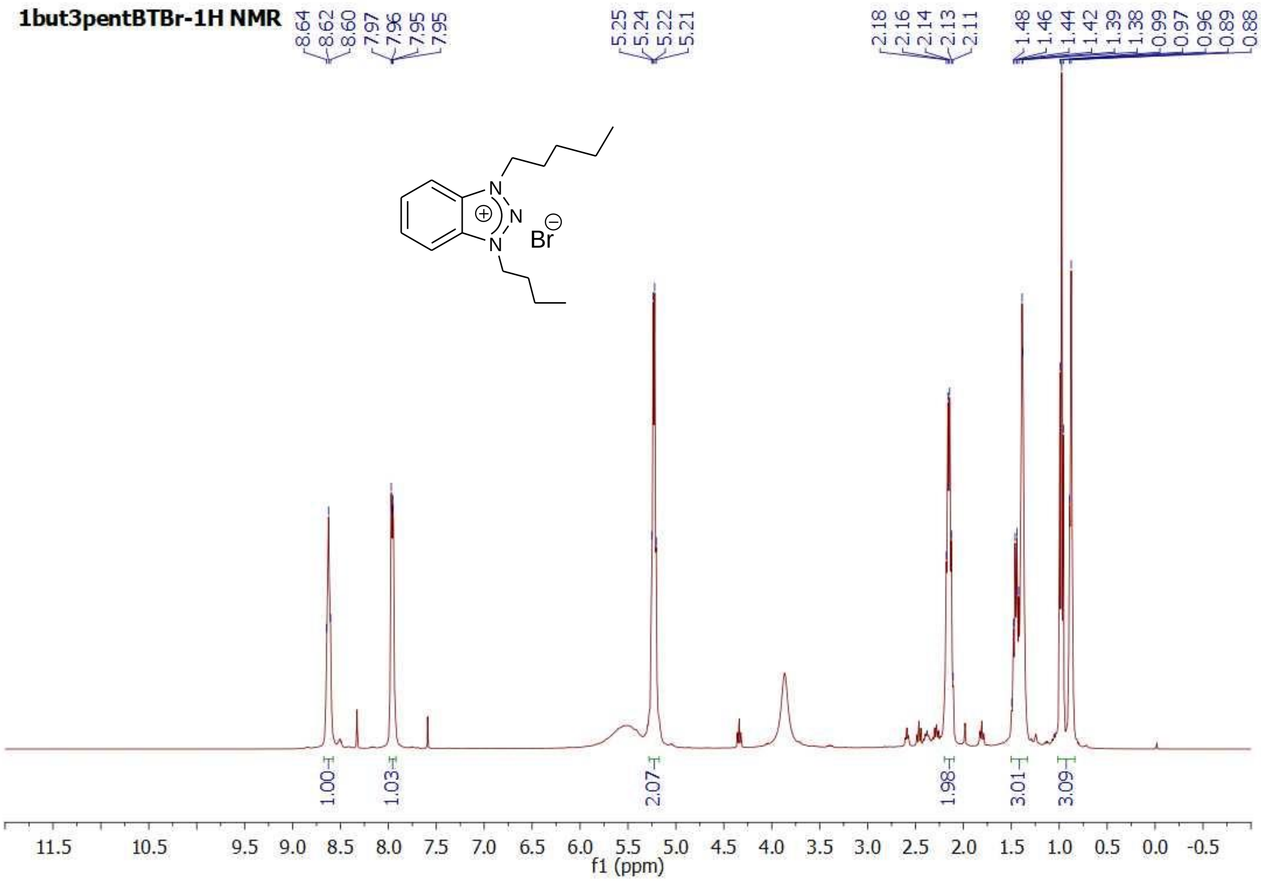

Figure S24.1but3pentBTBr-^{1}H NMR

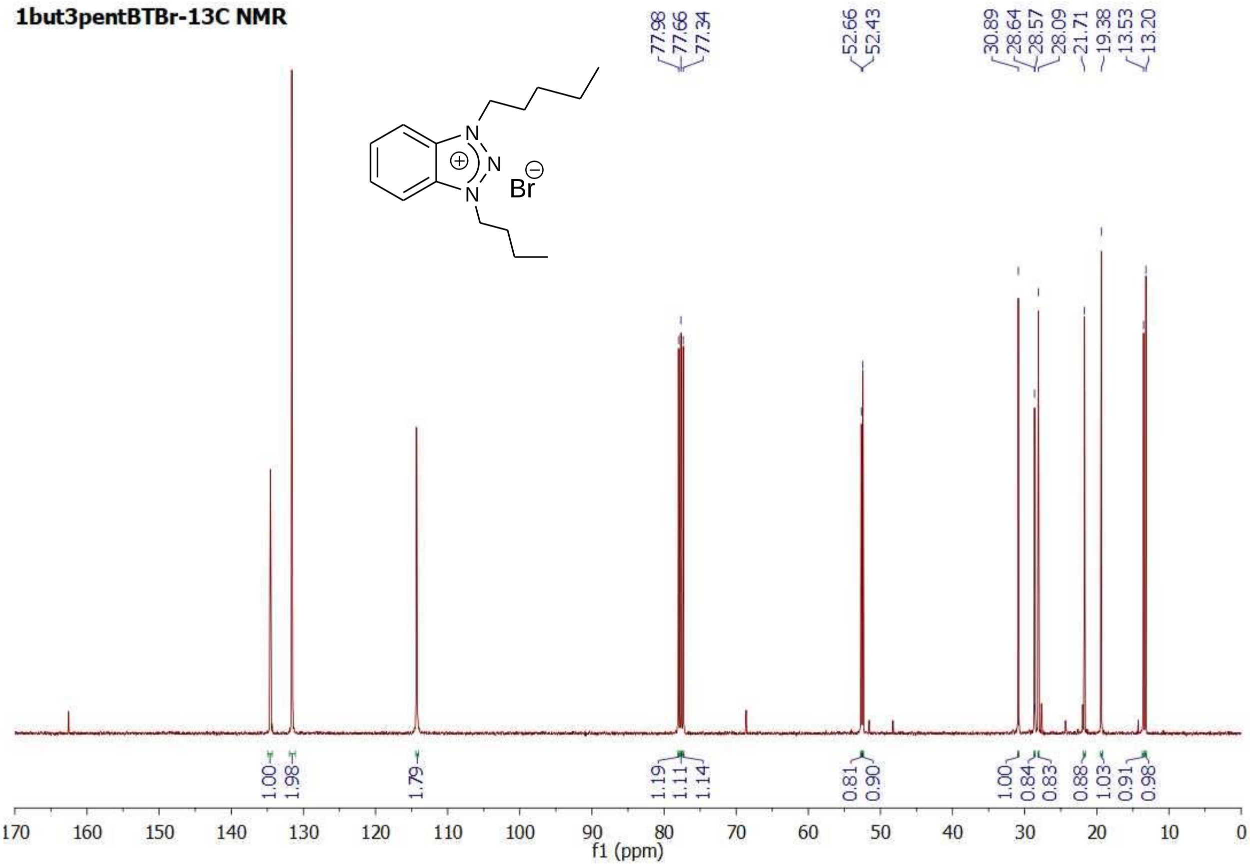

Figure S25. 1but3pentBTBr-^{13}C NMR

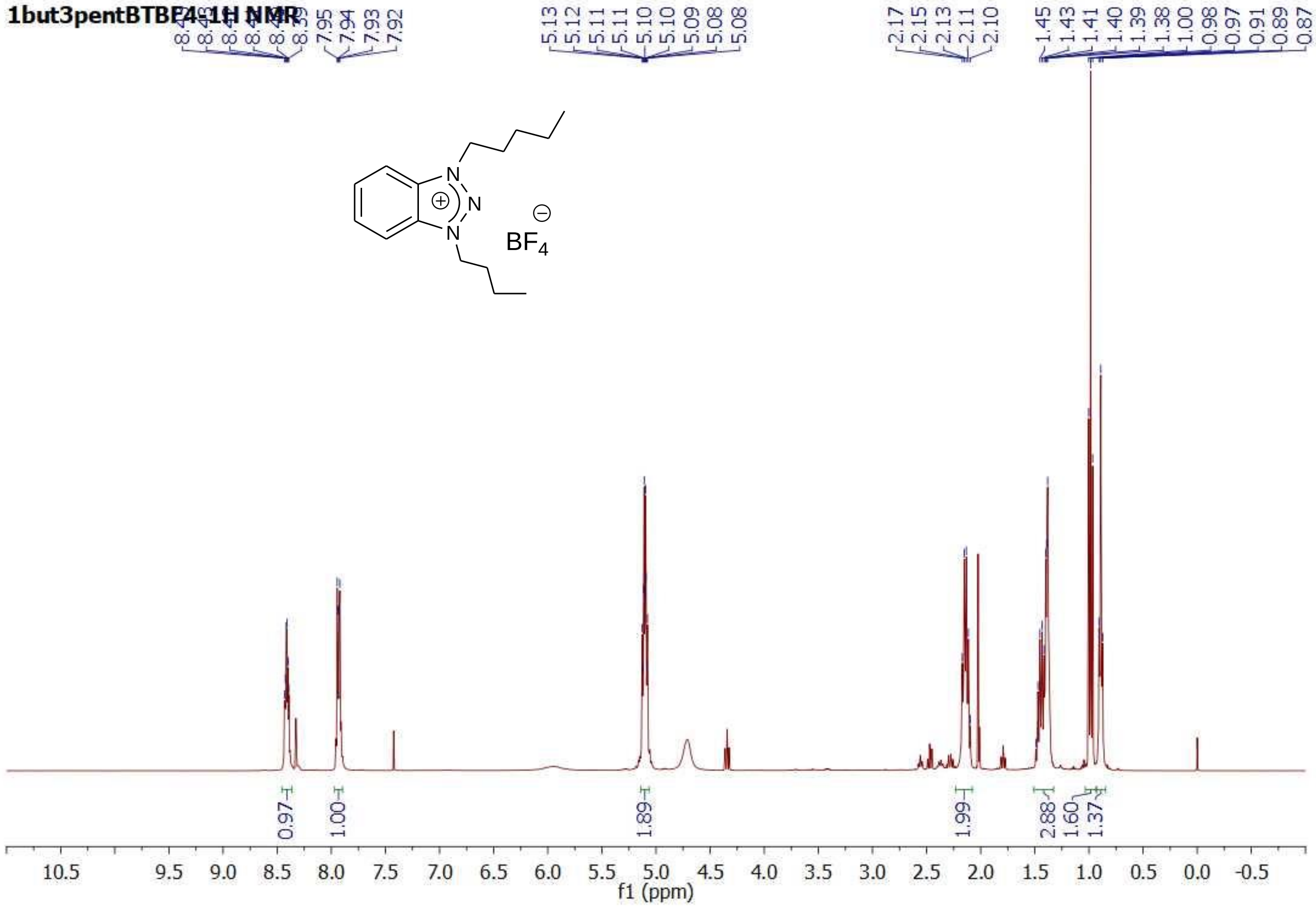

Figure S26. 1but3pentBTBF$_4$-^{1}H NMR

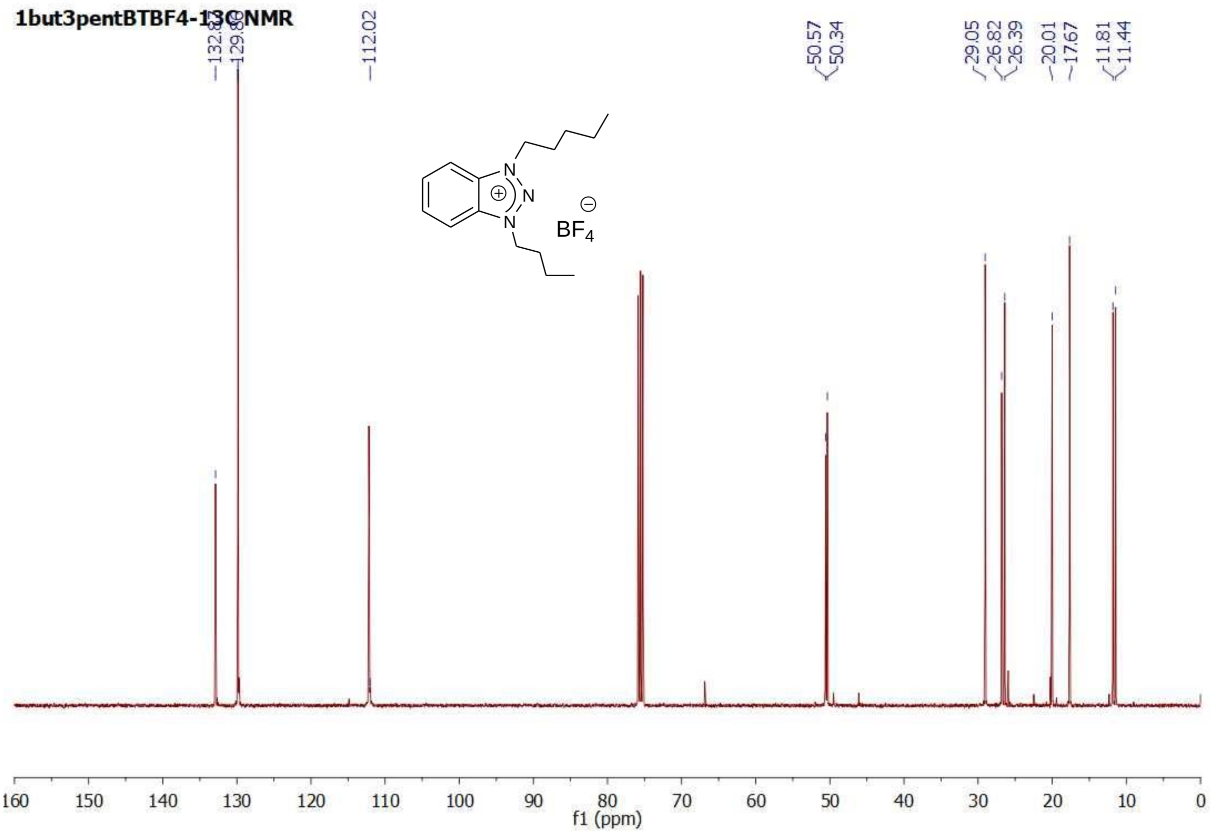

Figure S27. 1but3pentBTBF$_4$-^{13}C NMR

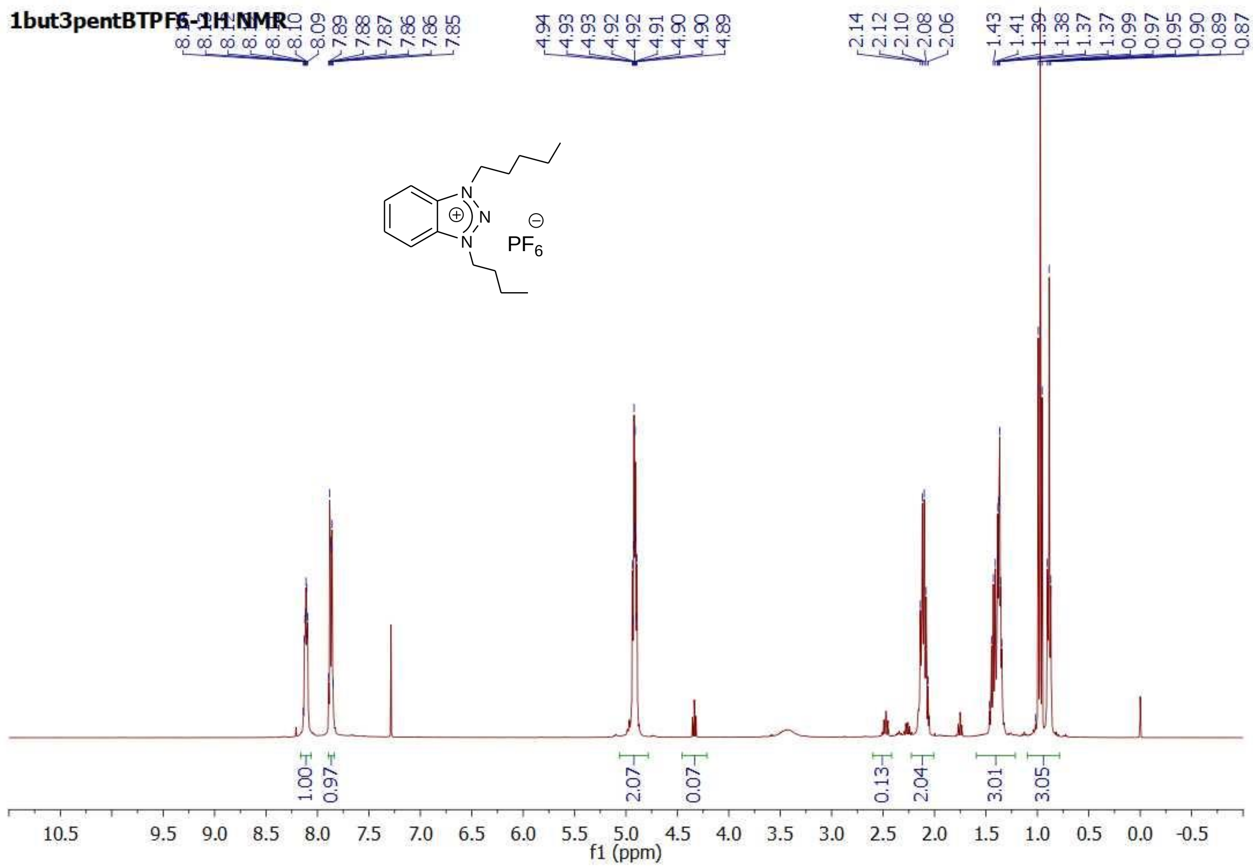

Figure S28. 1but3pentBTPF$_6$-^{1}H NMR

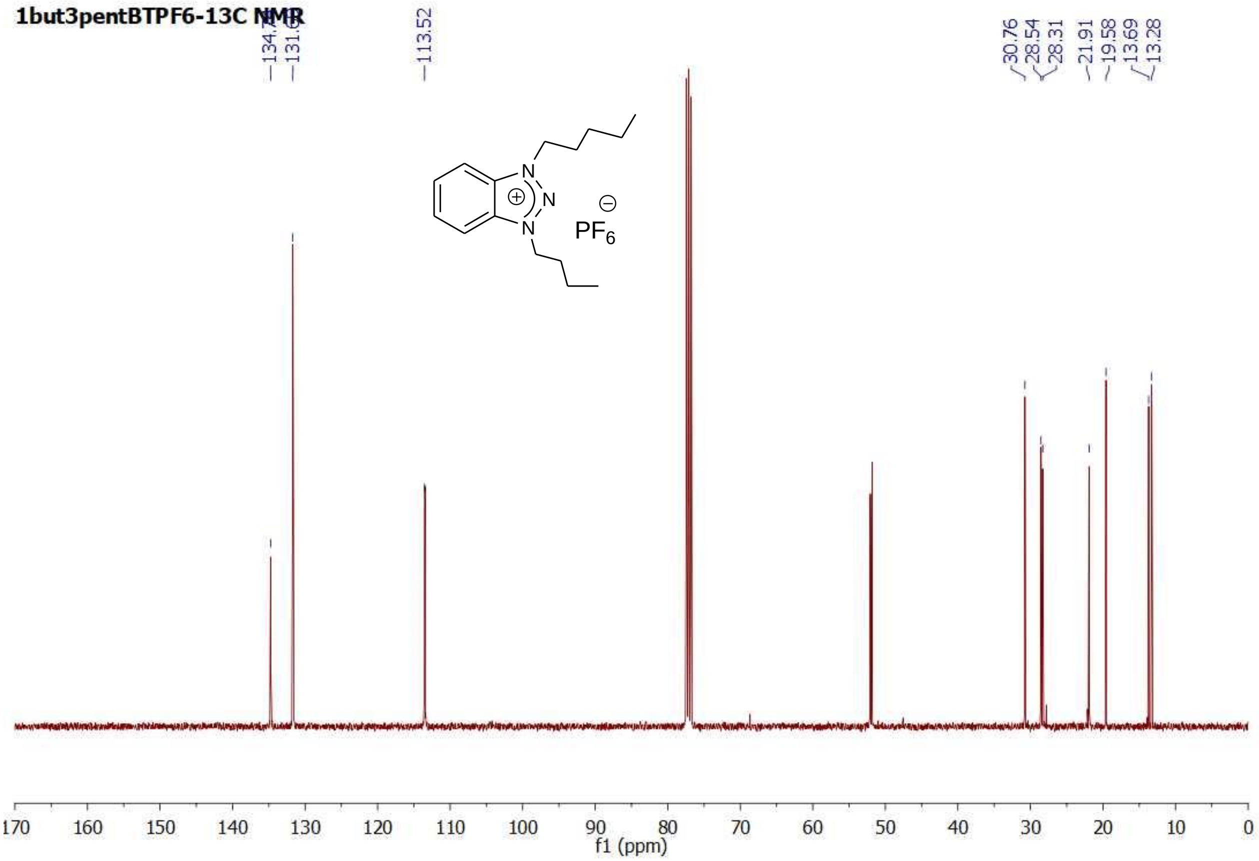

Figure S29.1but3pentBTPF$_6$-^{13}C NMR

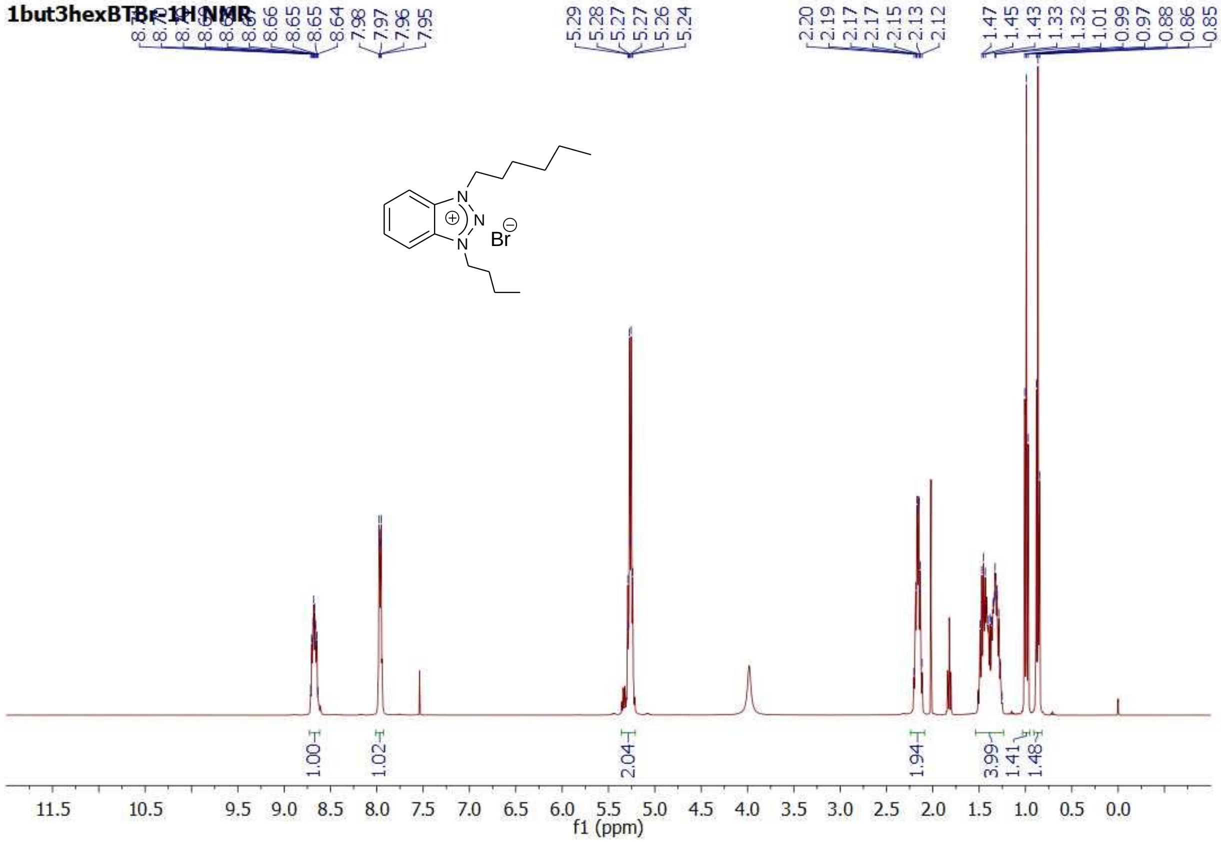

Figure S30.1but3hexBTBr-^{1}H NMR

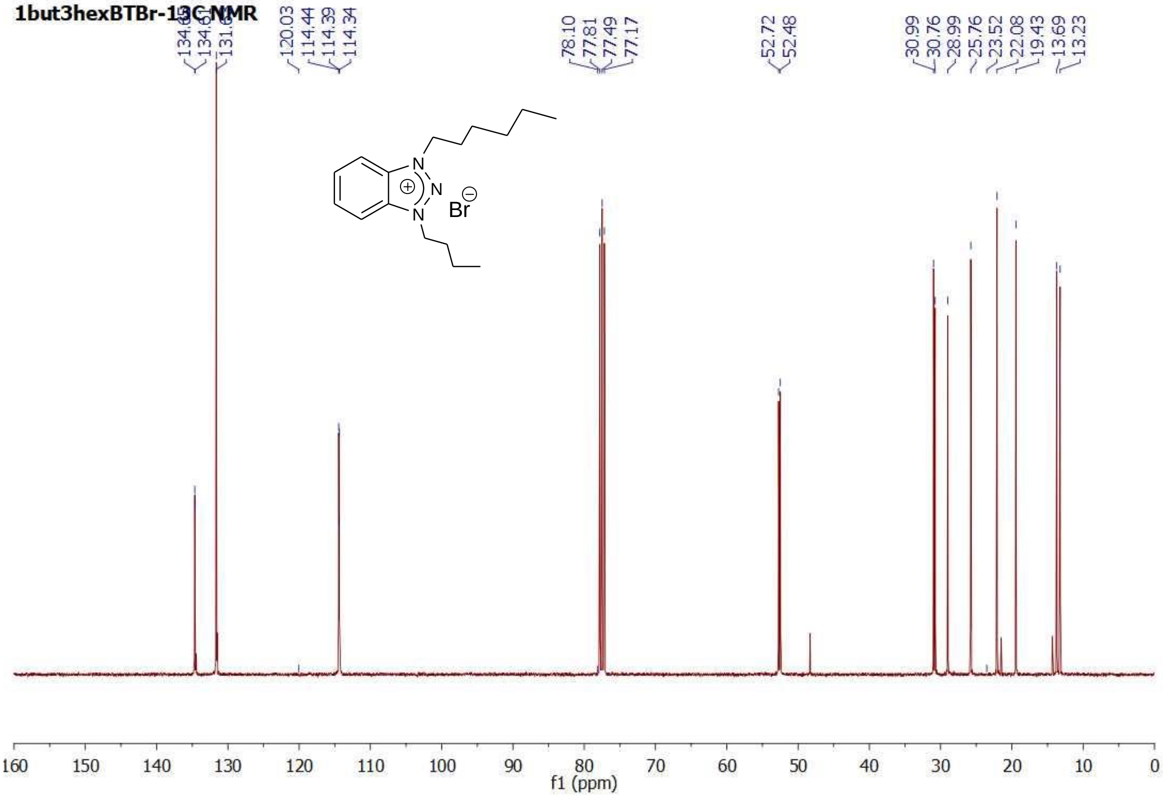

Figure S31. 1but3hexBTBr-^{13}C NMR

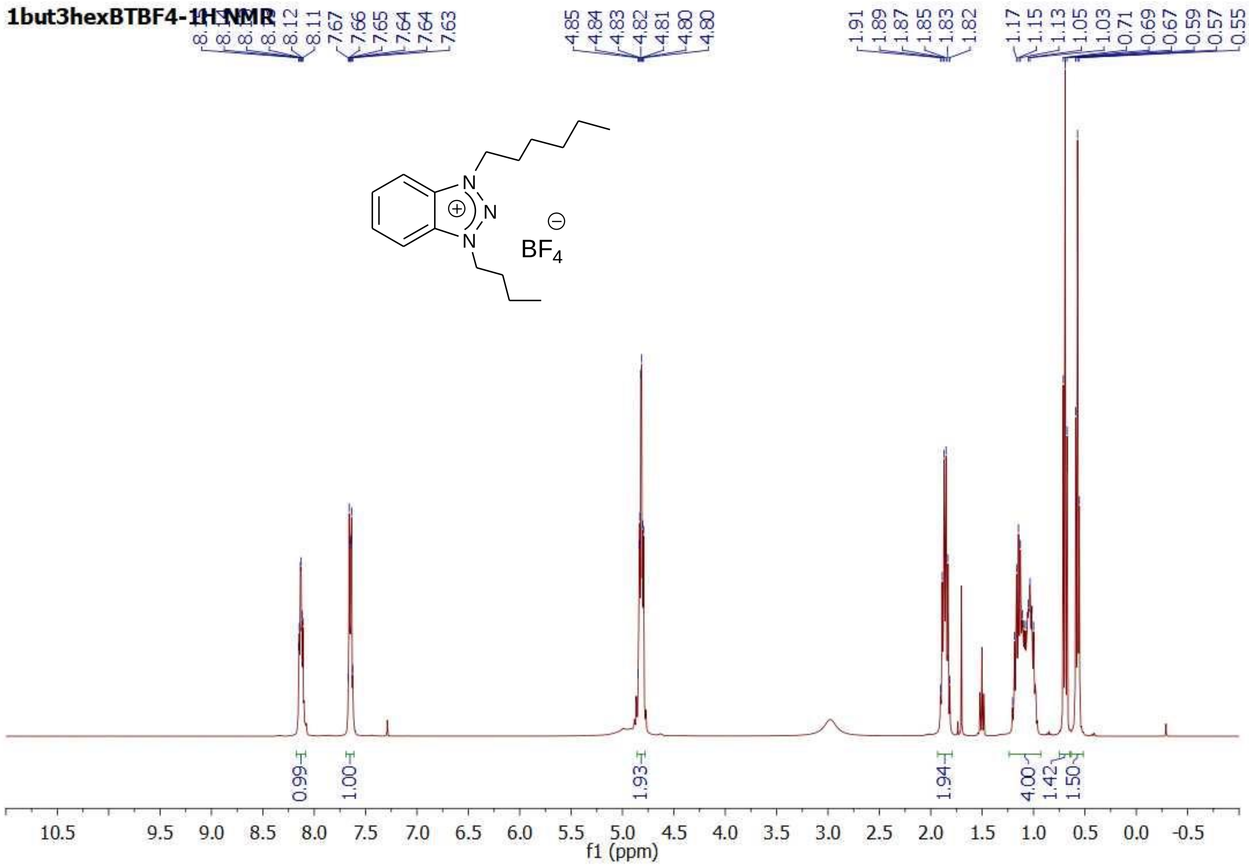

Figure S32. 1but3hexBTBF_4-^{1}H NMR

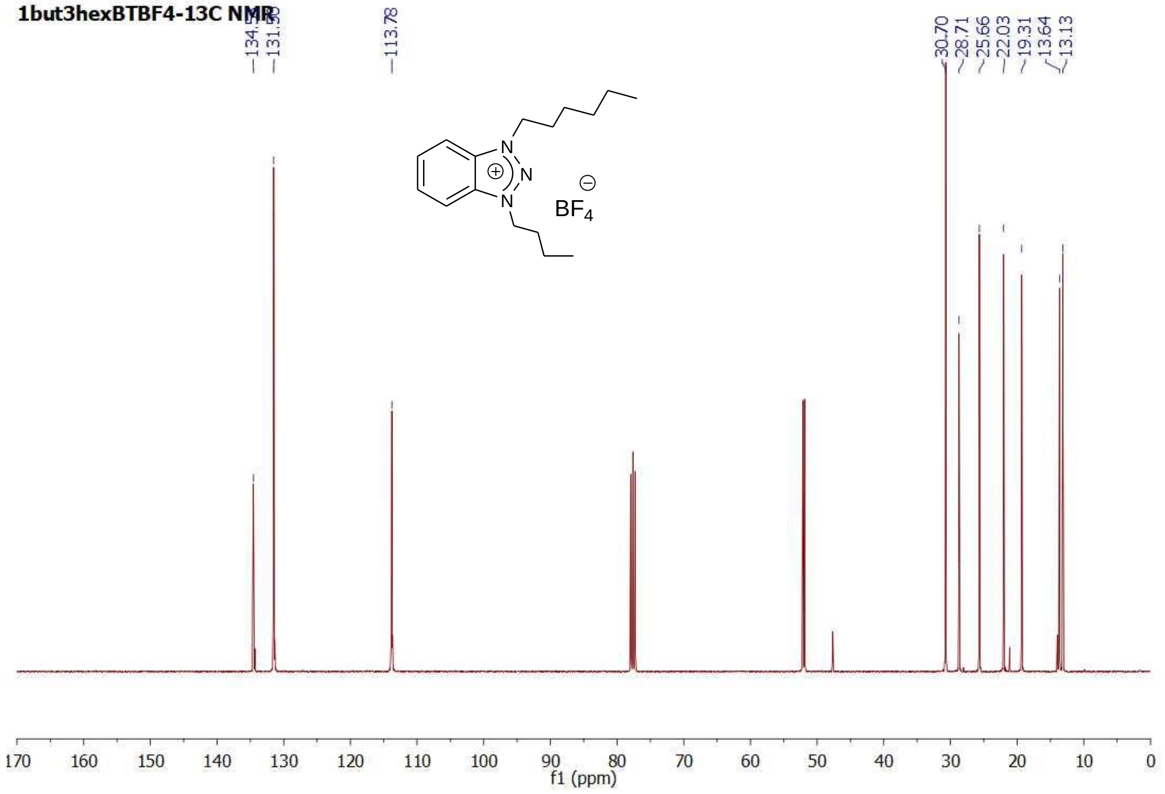

Figure S33. 1but3hexBTBF$_4$-^{13}C NMR

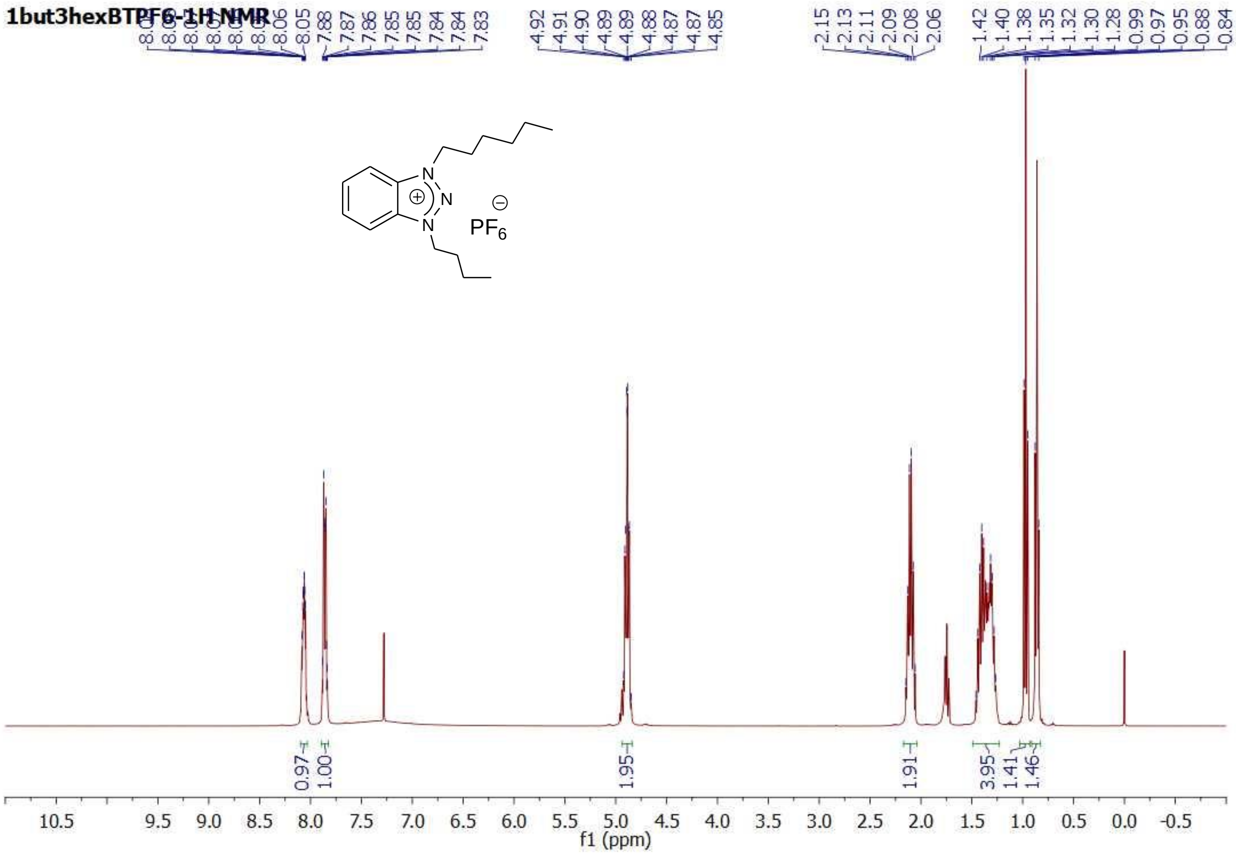

Figure S34. 1but3hexBTPF$_6$-^{1}H NMR

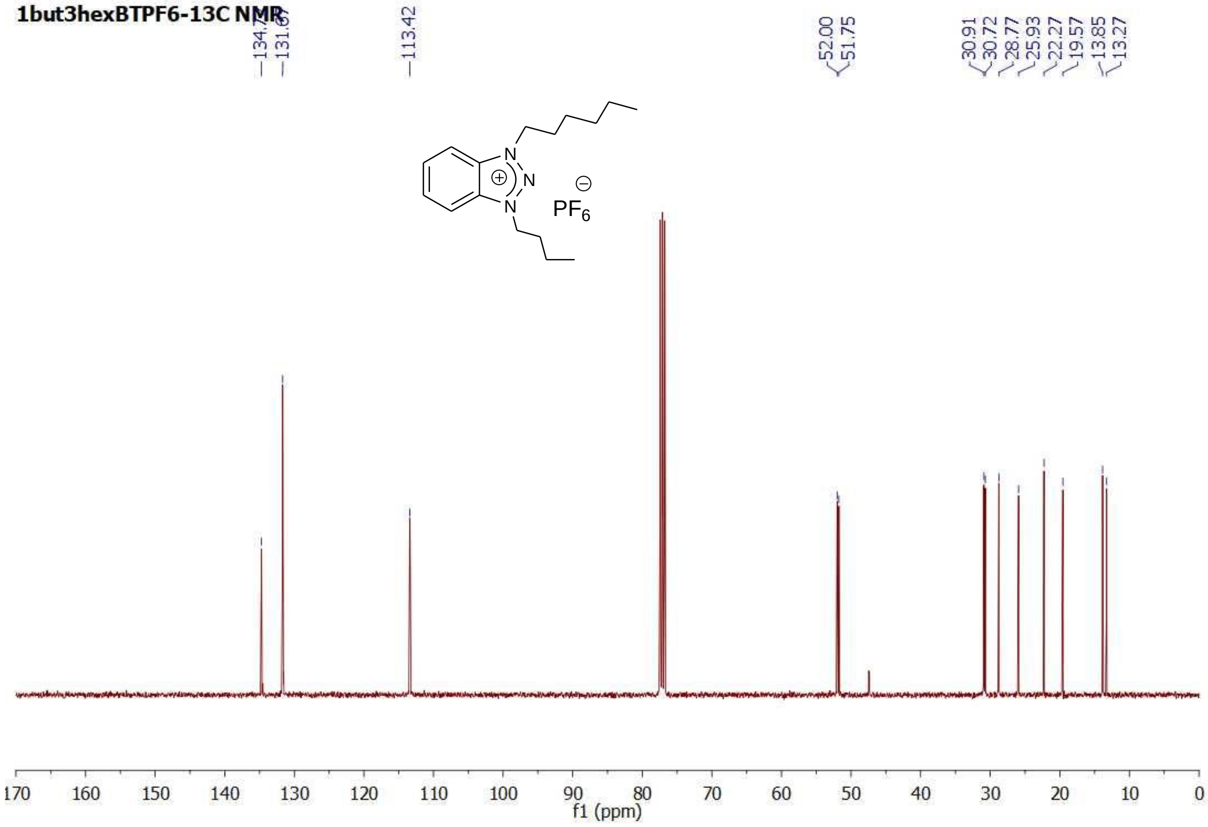

Figure S35. 1but3hexBTPF$_6$-^{13}C NMR

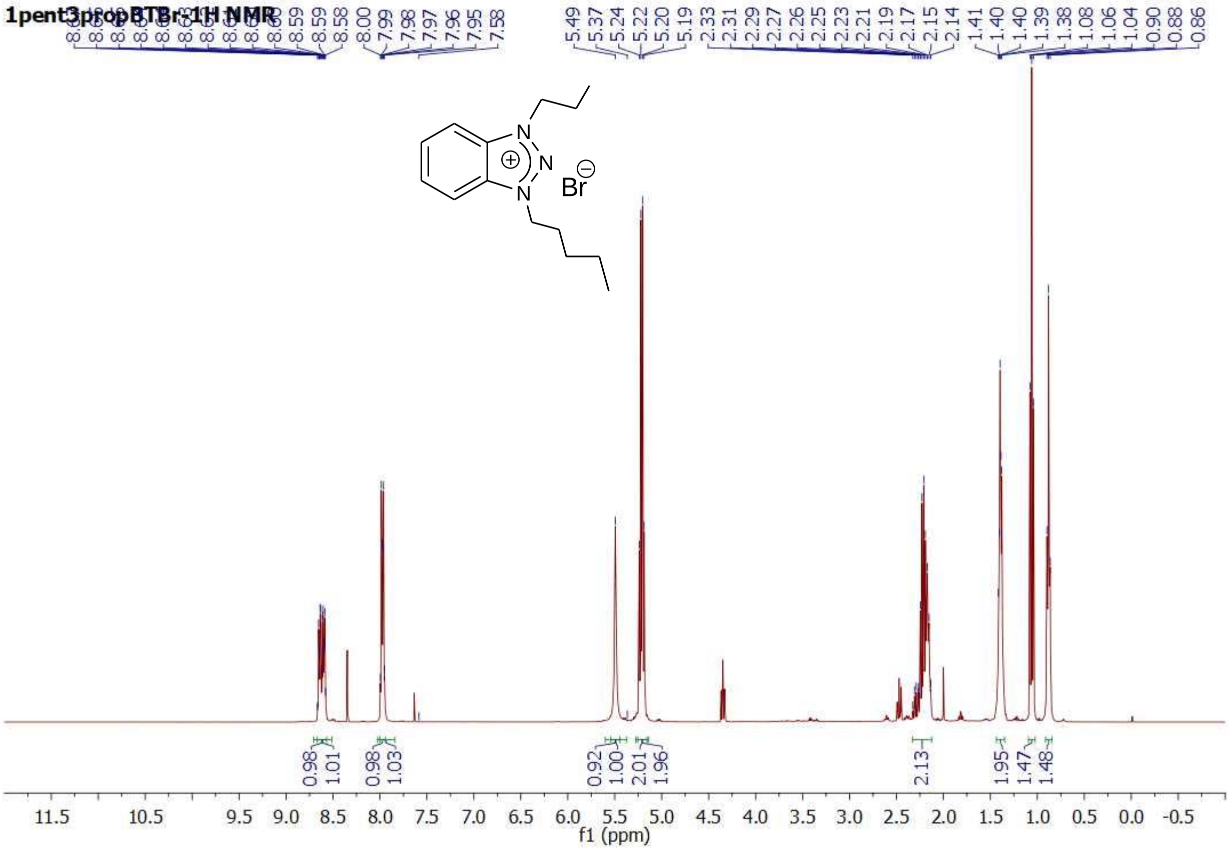

Figure S36. 1pent3propBTBr-^{1}H NMR

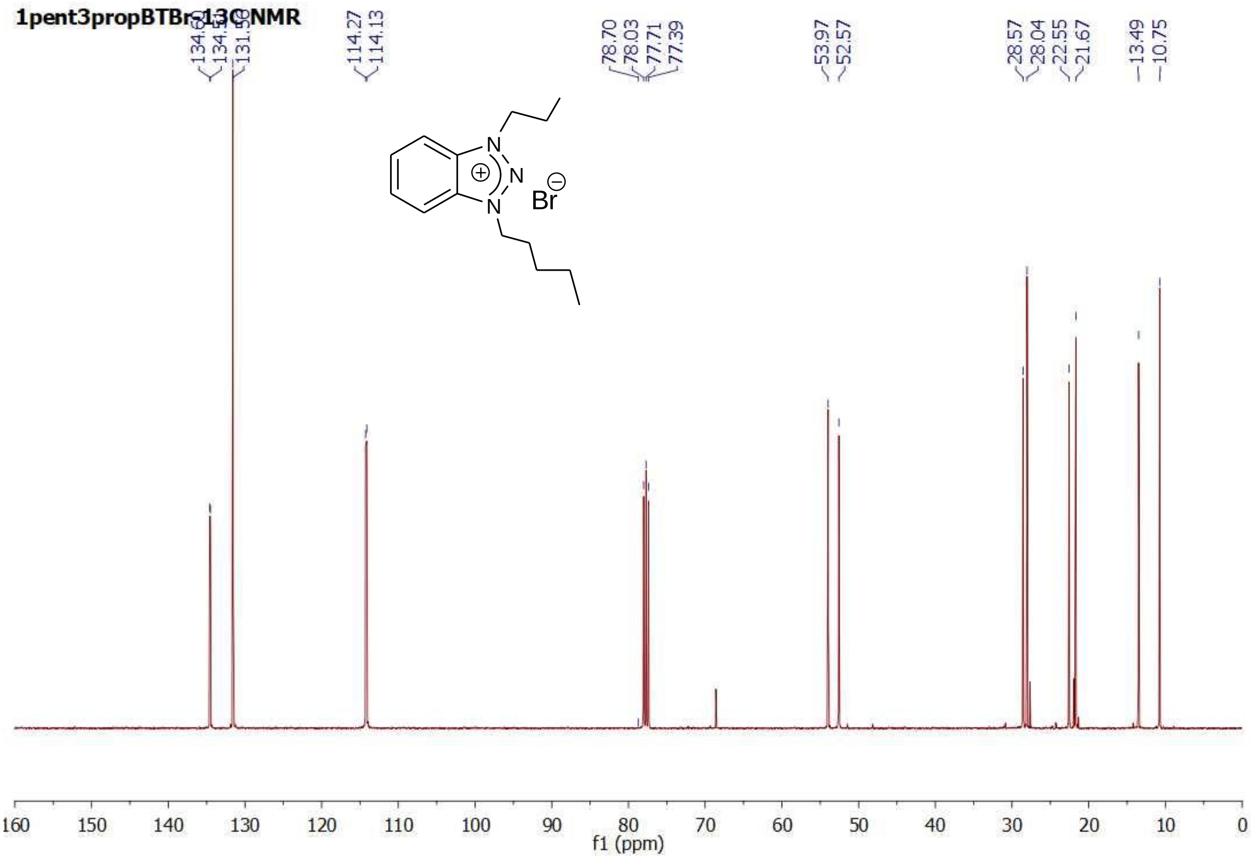

Figure S37. 1pent3propBTBr-^{13}C NMR

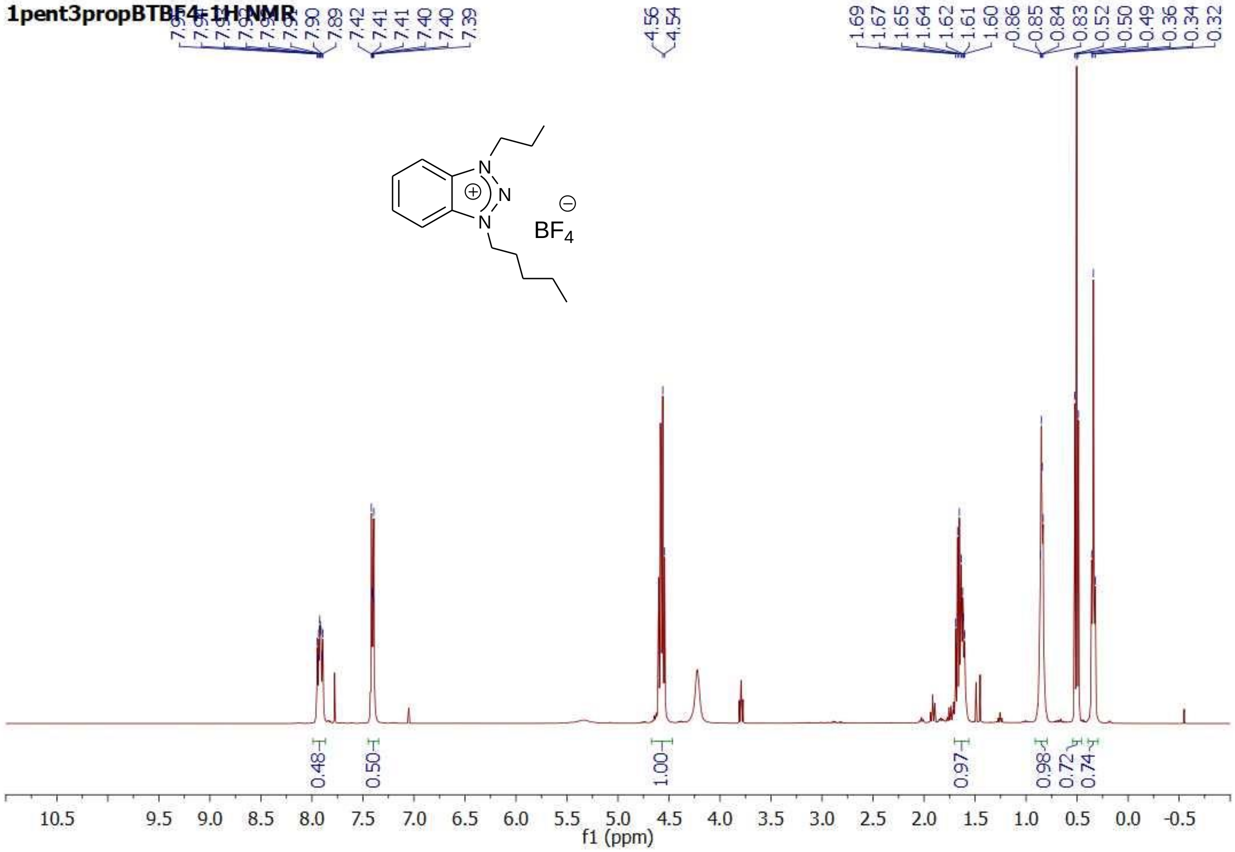

Figure S38. 1pent3propBTBF_4-^{1}H NMR

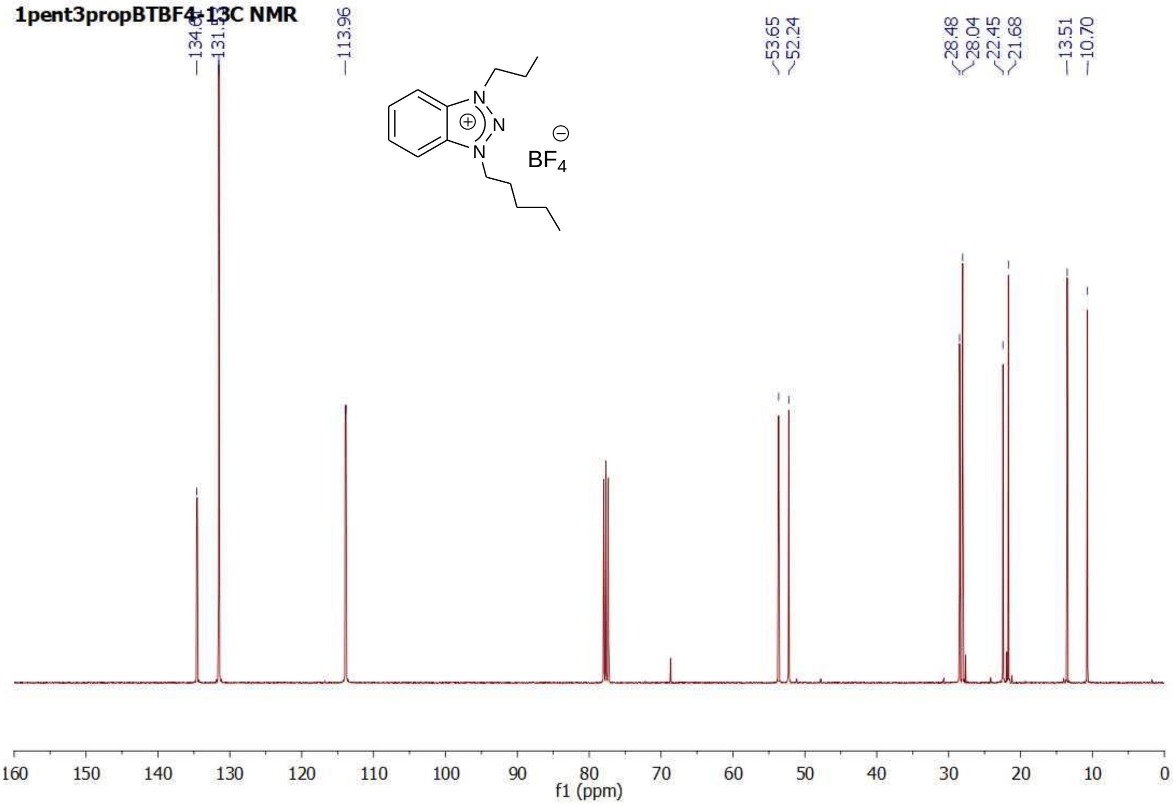

Figure S39.1pent3propBTBF$_4$-^{13}C NMR

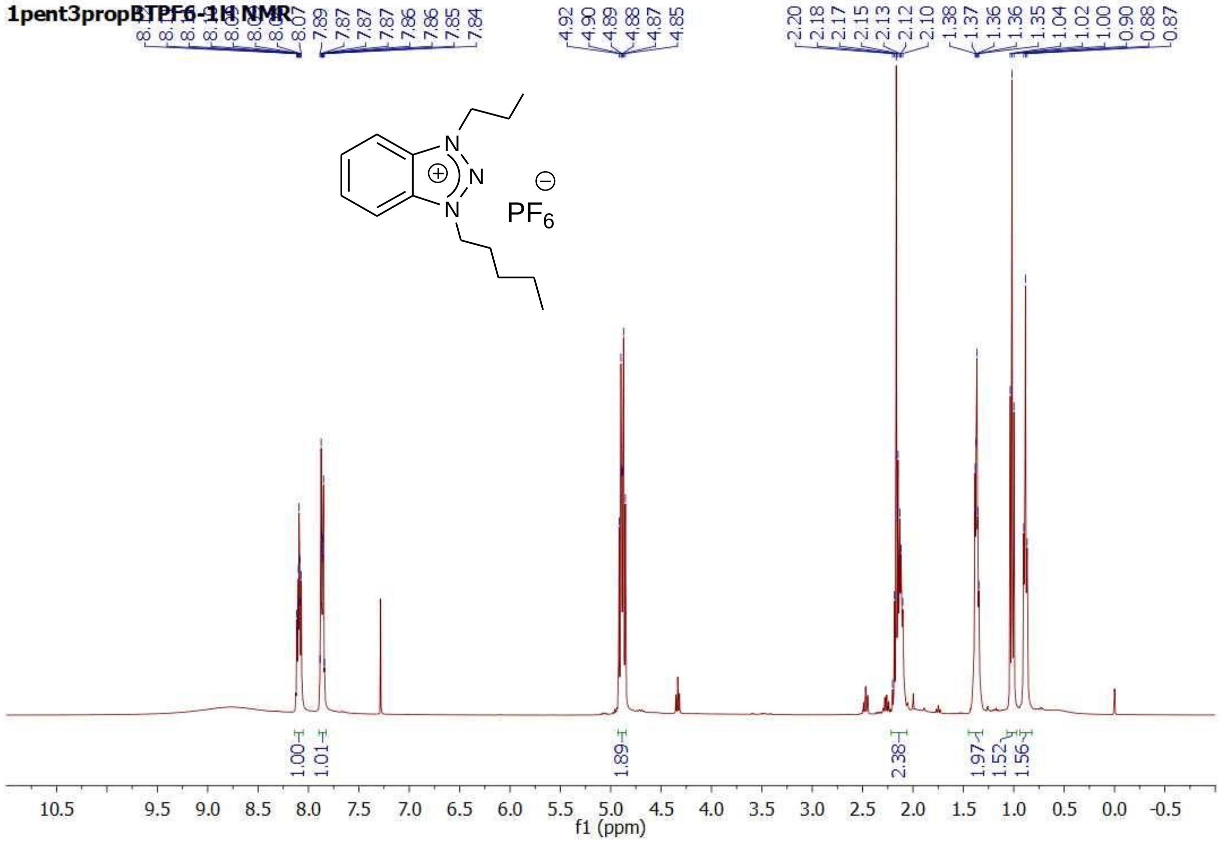

Figure S40. 1pent3propBTPF$_6$-^{1}H NMR

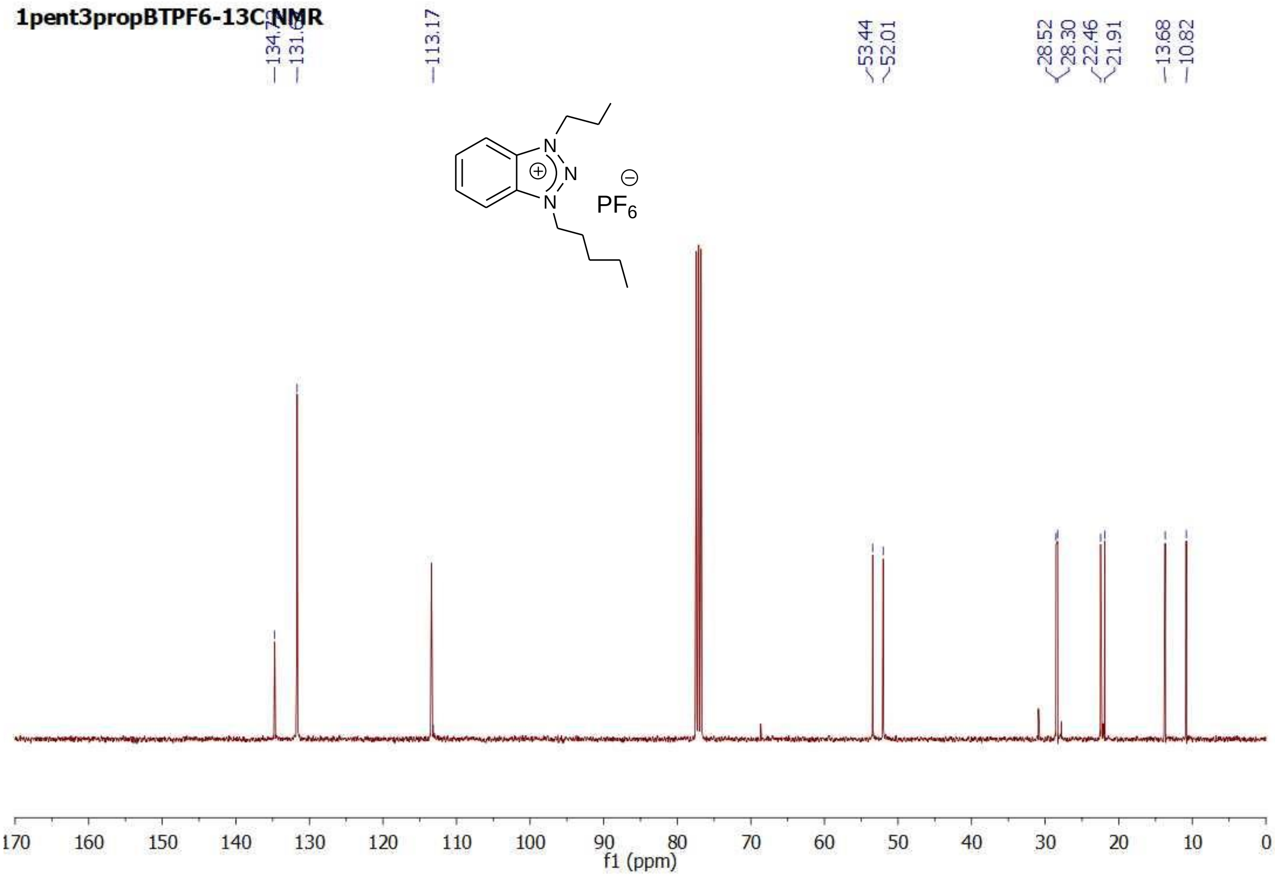

Figure S41. 1pent3propBTPF$_6$-^{13}C NMR

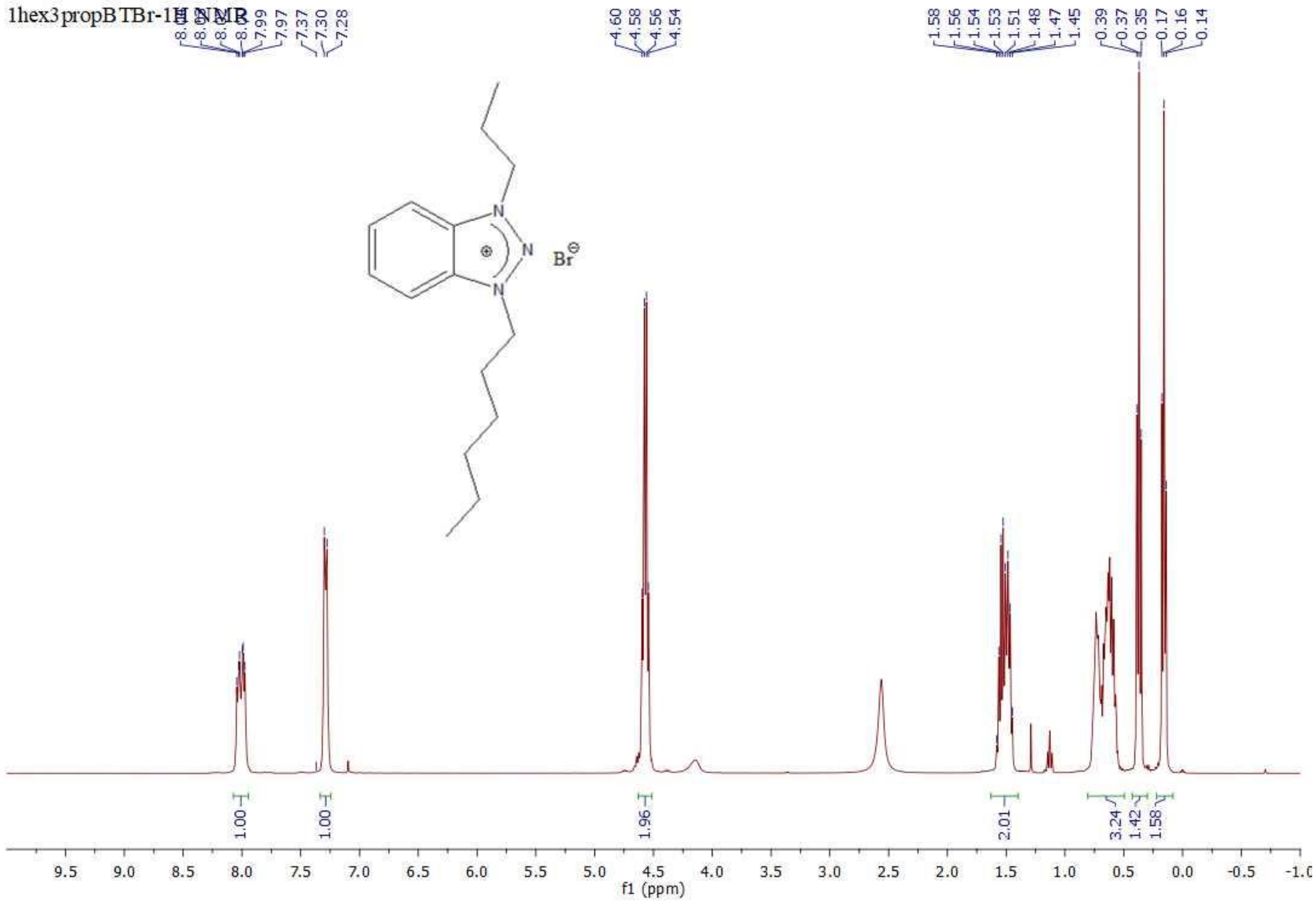

Figure S42.1hex3propBTBr-^{1}H NMR

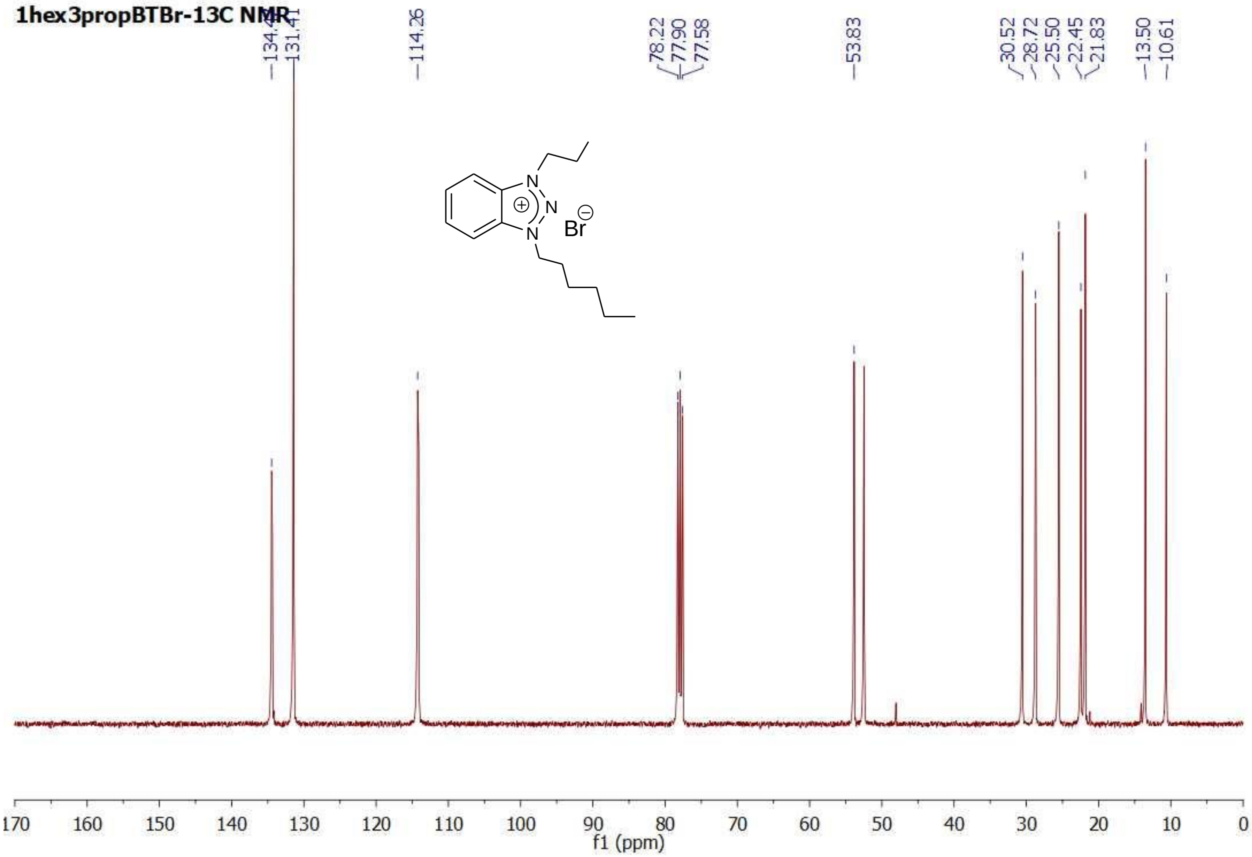

Figure S43. 1hex3propBTBr-^{13}C NMR

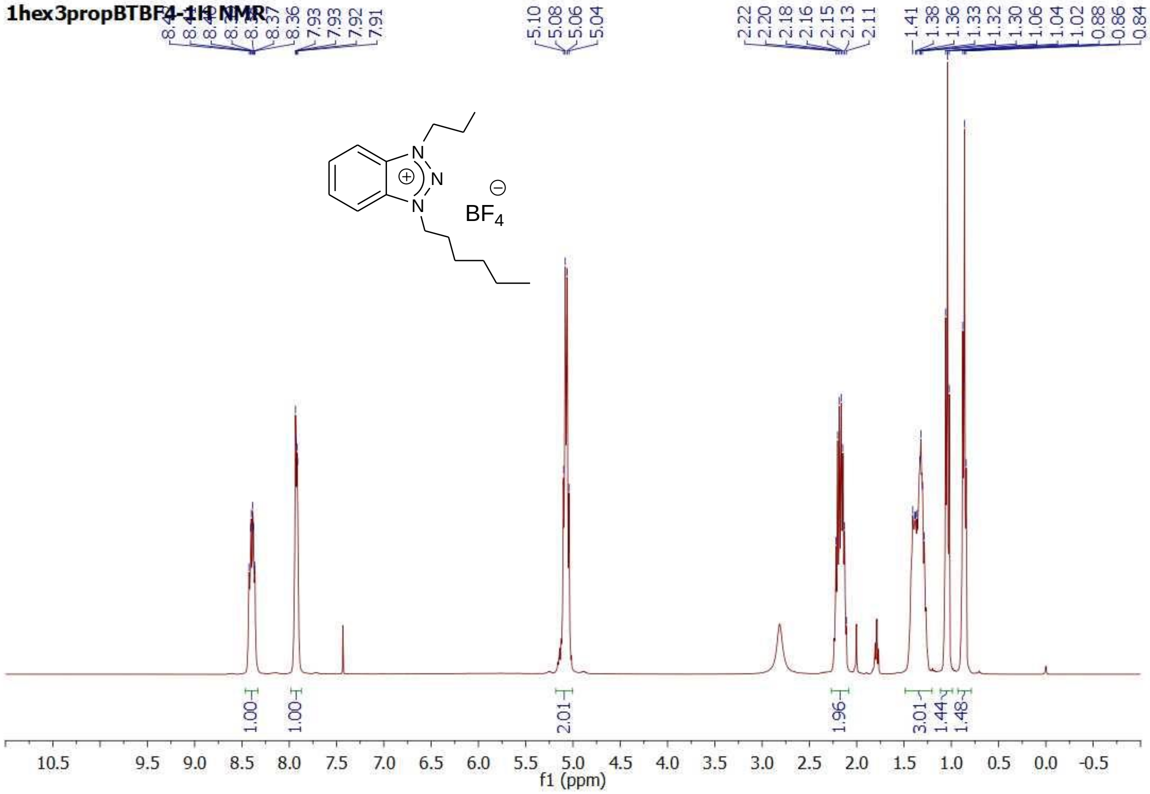

Figure S44. 1hex3propBTBF$_4$-^{1}H NMR

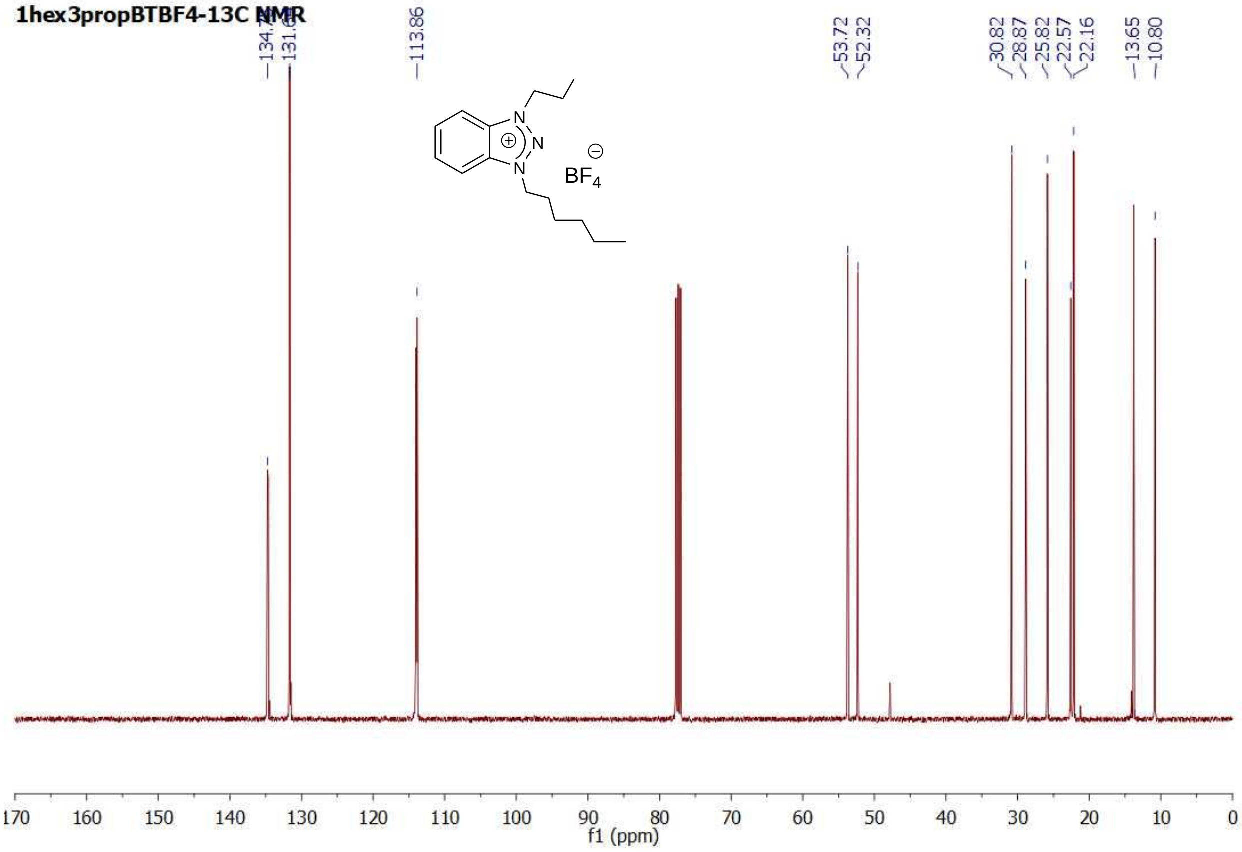

Figure S45. 1hex3propBTBF$_4$-^{13}C NMR

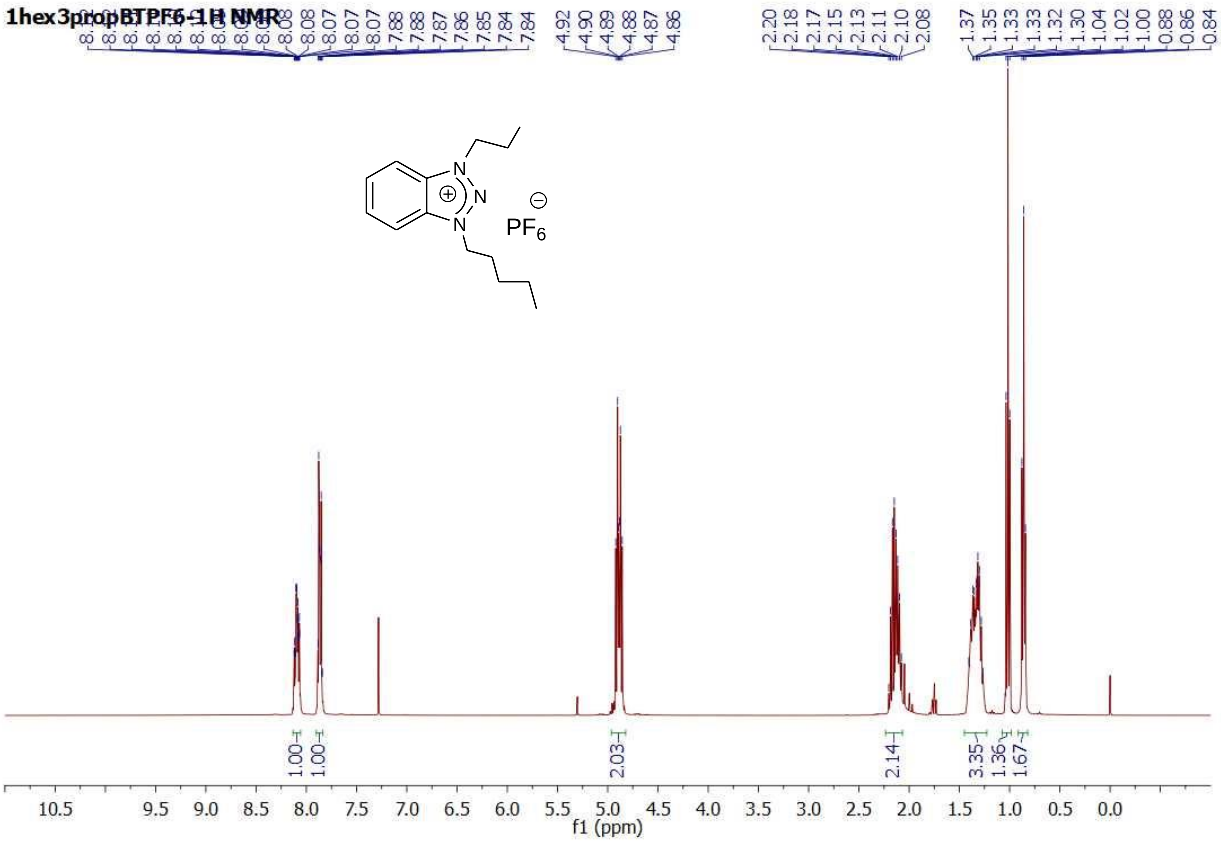

Figure S46. 1hex3propBTPF$_6$-^{1}H NMR

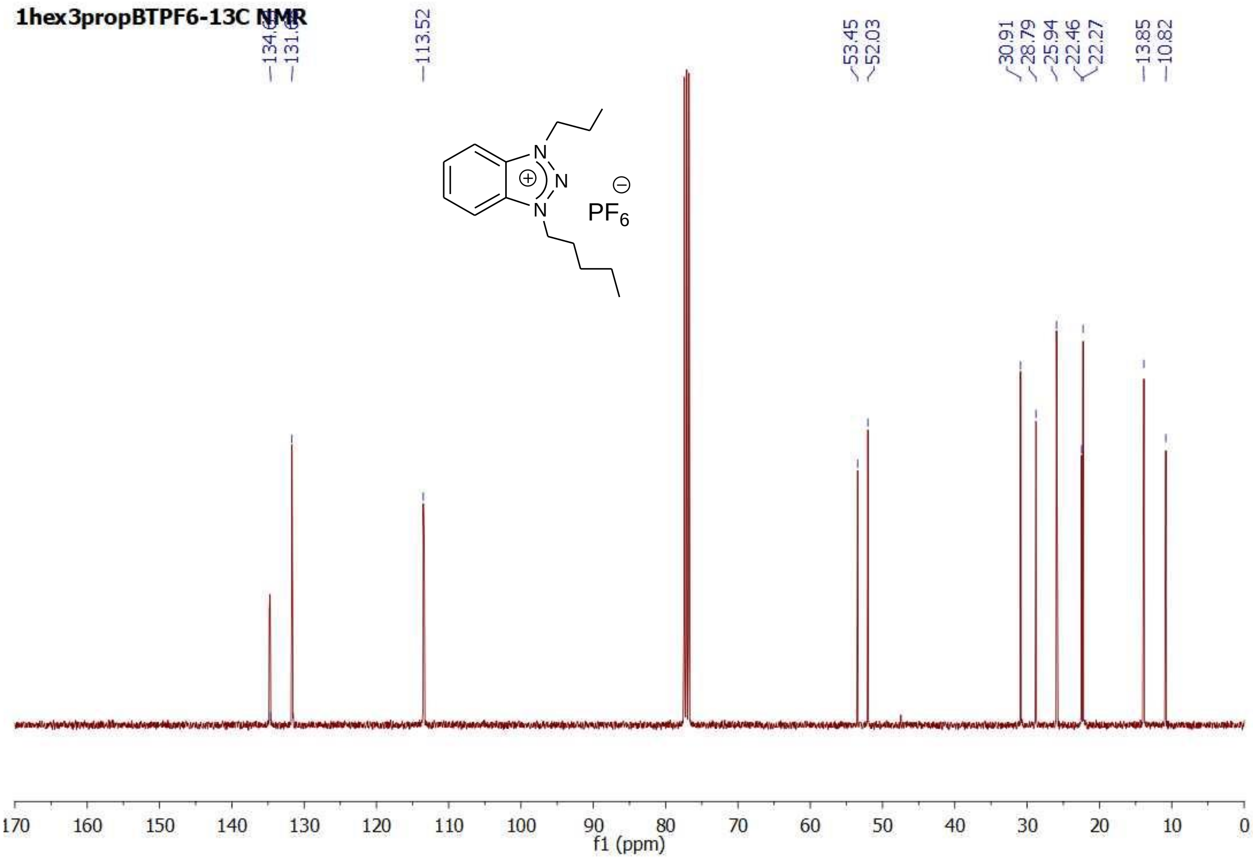

Figure S47. 1hex3propBTPF$_6$-^{13}C NMR

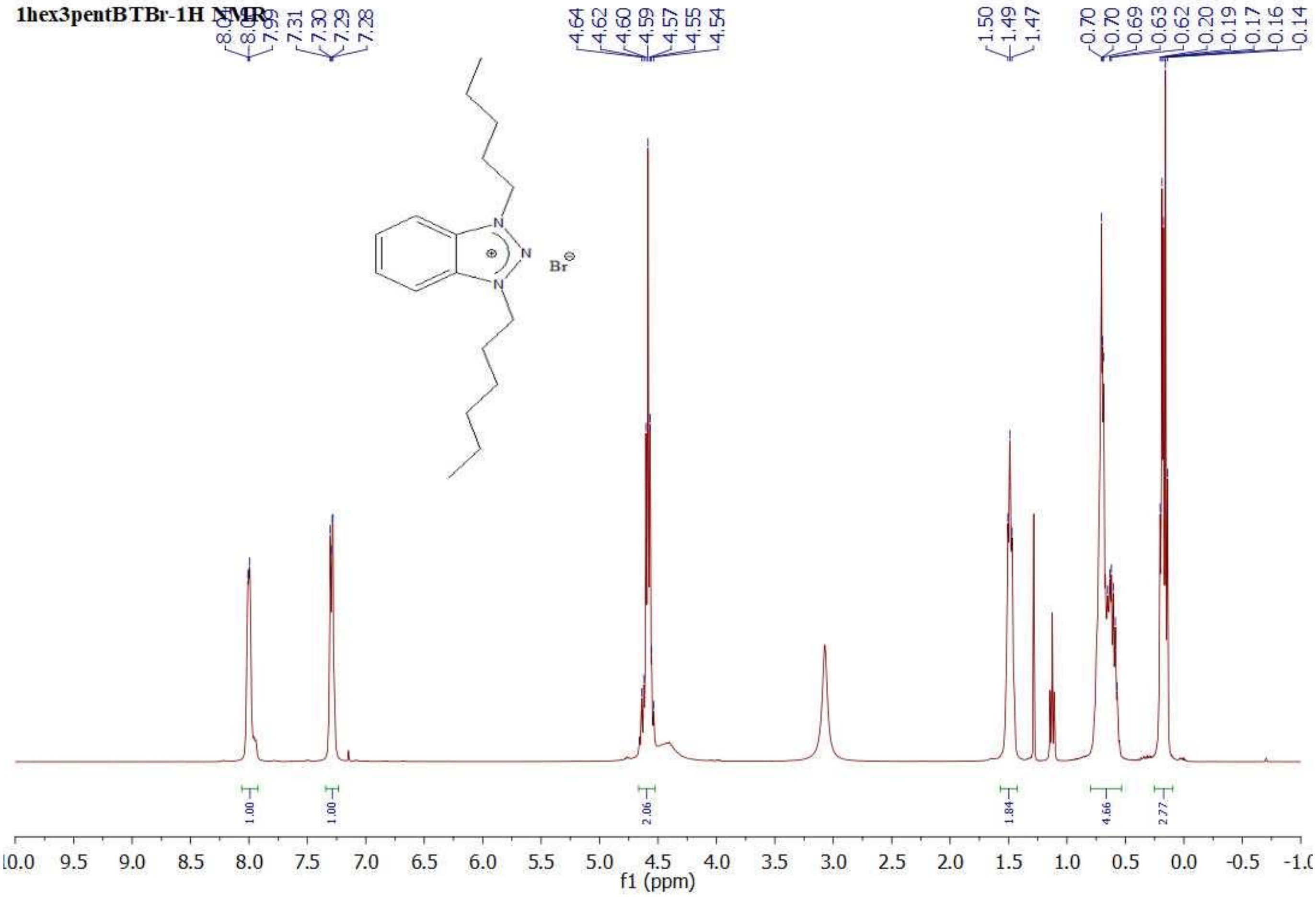

Figure S48. 1hex3pentBTBr-^{1}H NMR

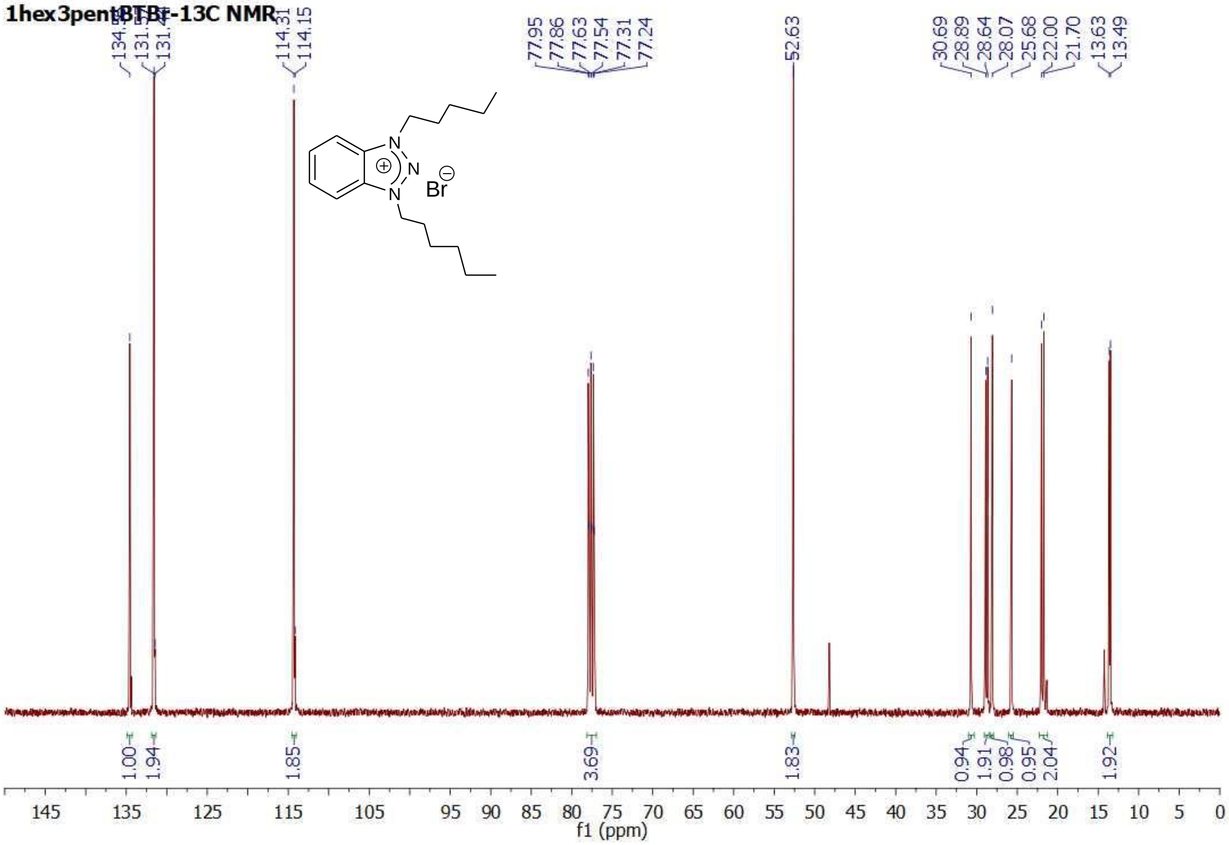

Figure S49. 1hex3pentBTBr-^{13}C NMR

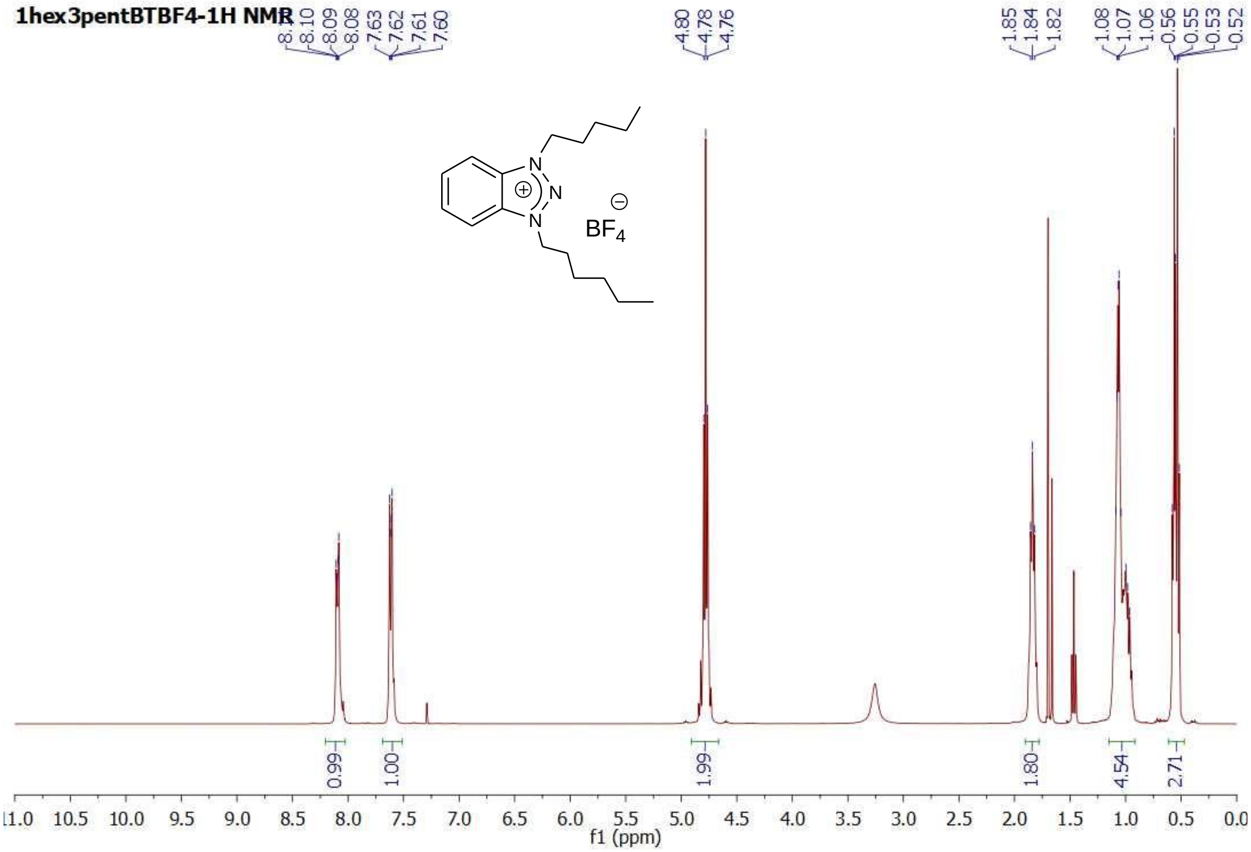

Figure S50. 1hex3pentBTBF_4-^{1}H NMR

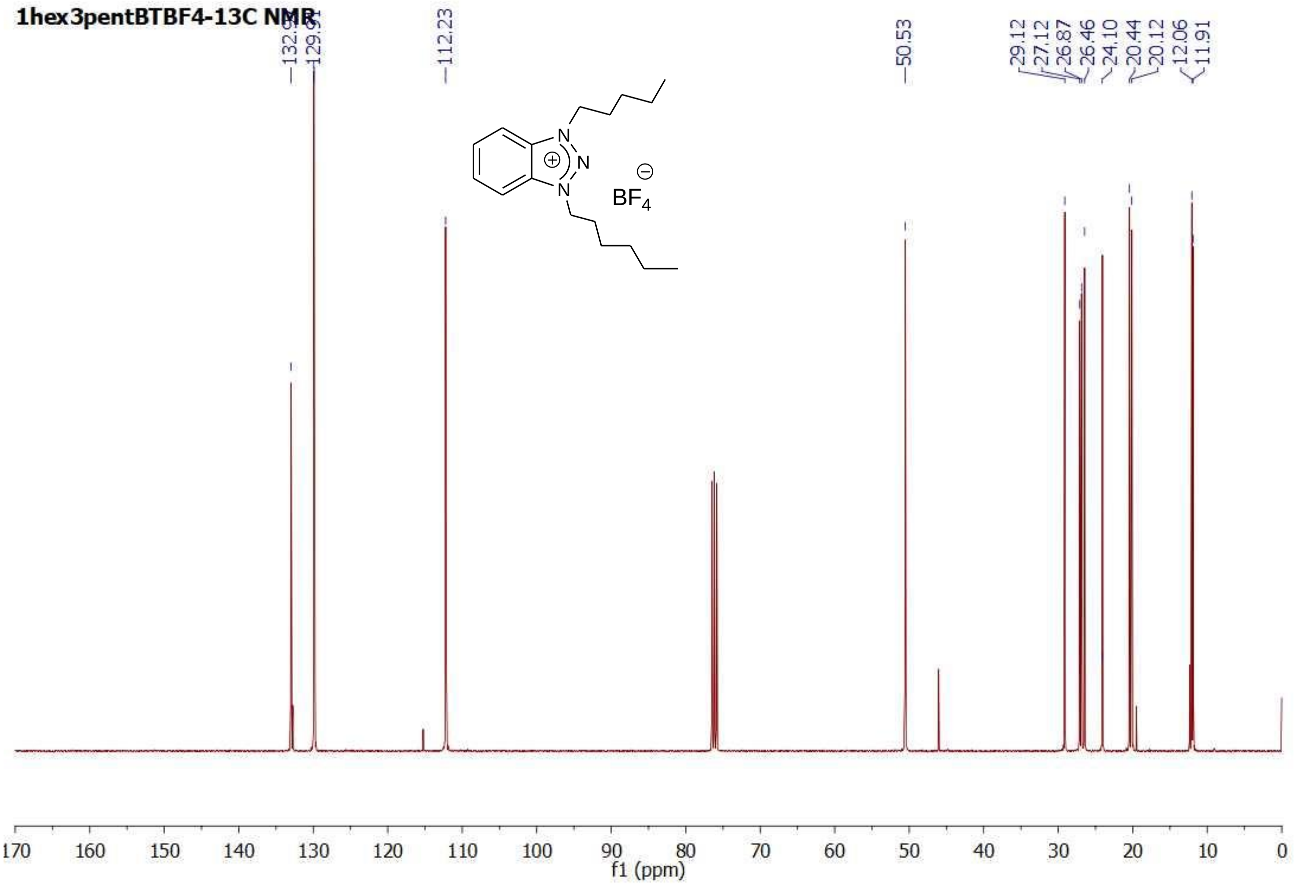

Figure S51. 1hex3pentBTBF$_4$-^{13}C NMR

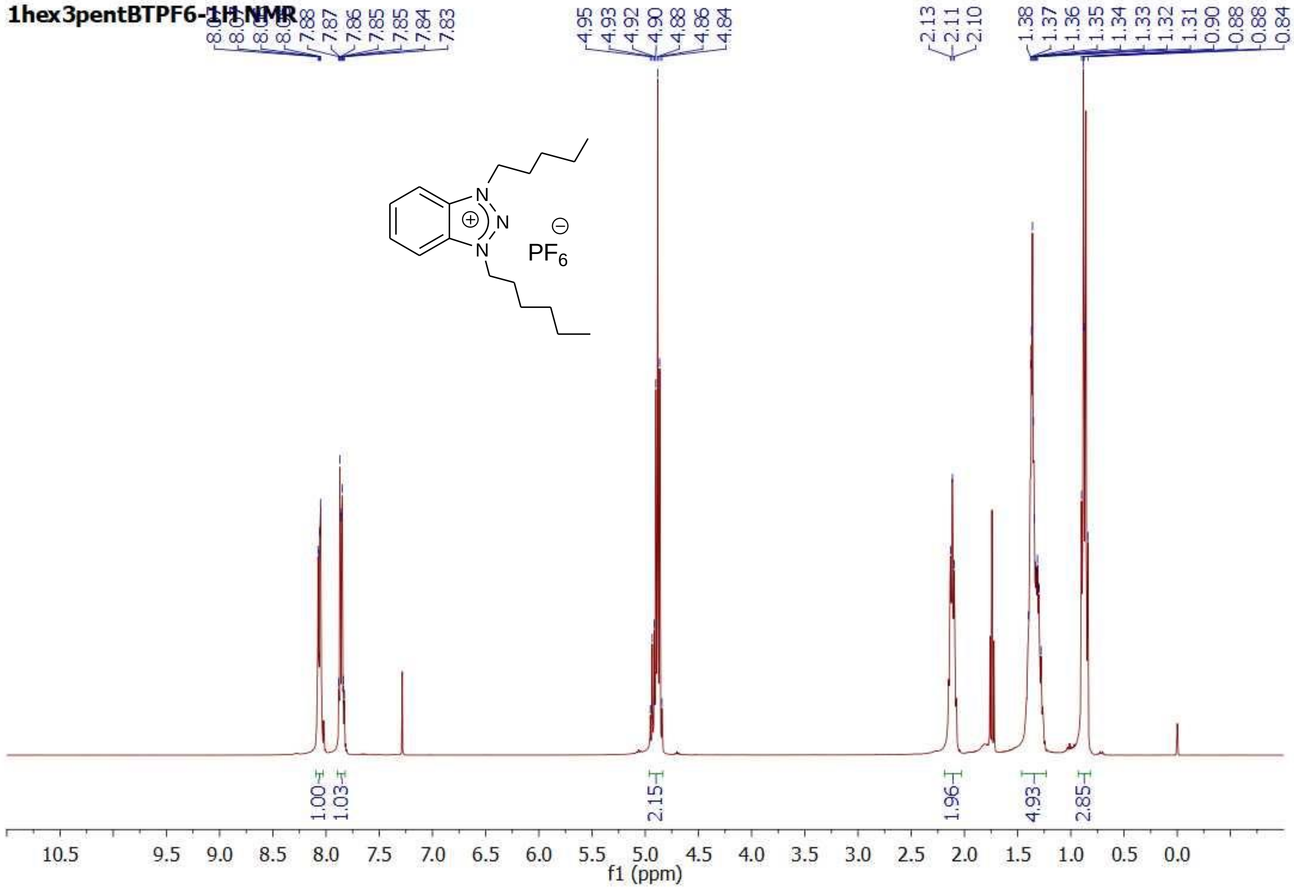

Figure S52. 1hex3pentBTPF_6-^{1}H NMR

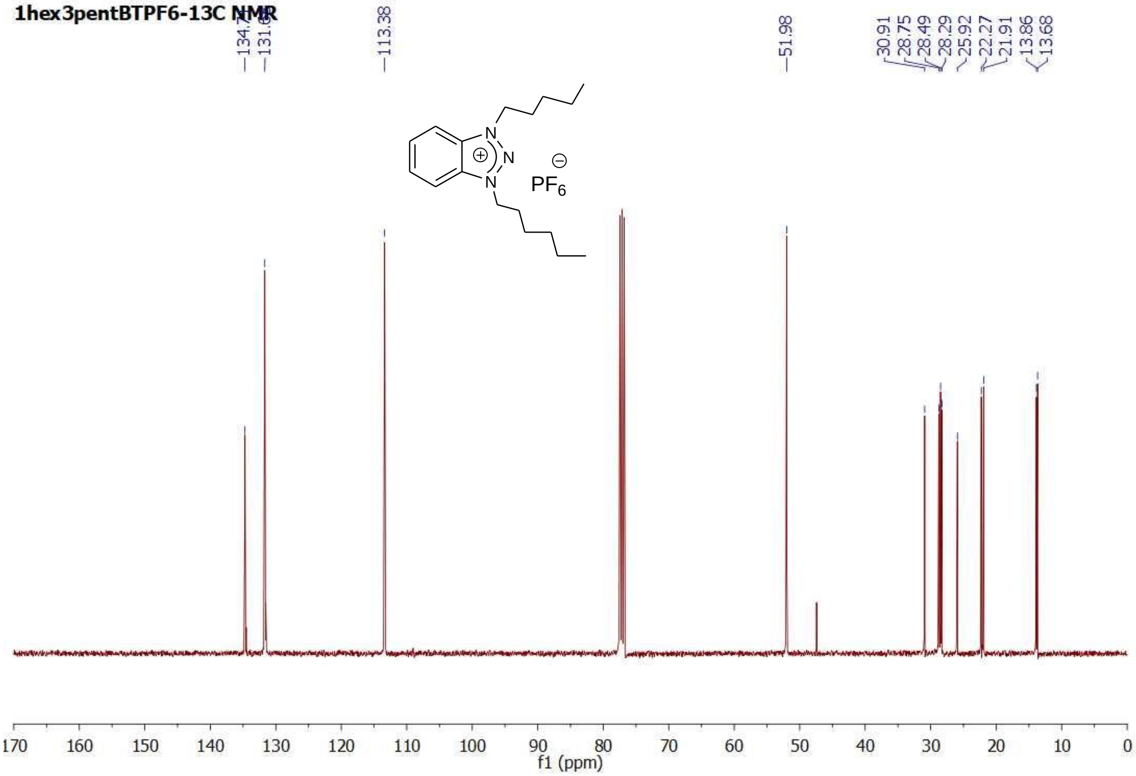

Figure S53. 1hex3pentBTPF$_6$-^{13}C NMR

S3. FTIR

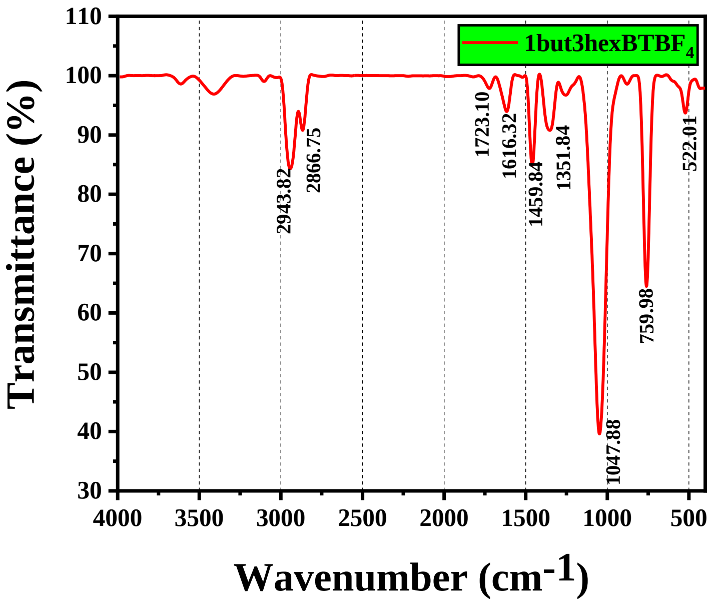

Figure S54. 1but3hex$BTBF_4$

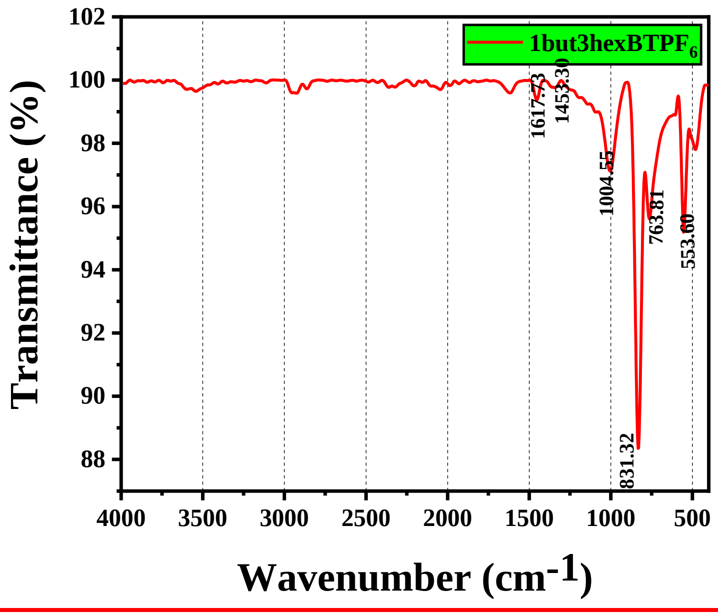

Figure S55. 1but3hex$BTPF_6$

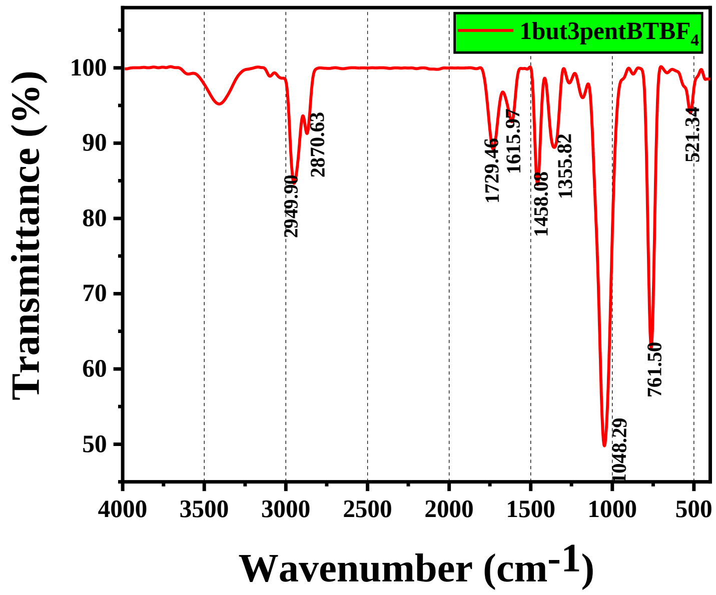

Figure S56. 1but3pentBTBF$_4$

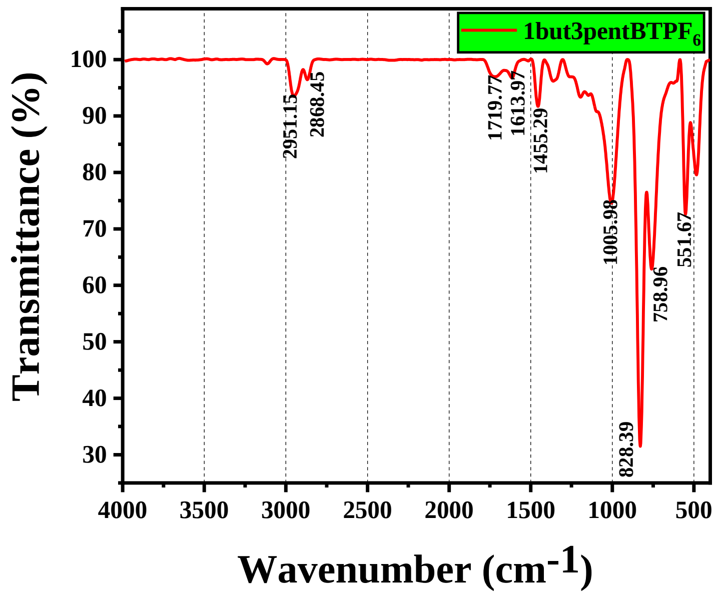

Figure S57. 1but3pentBTPF$_6$

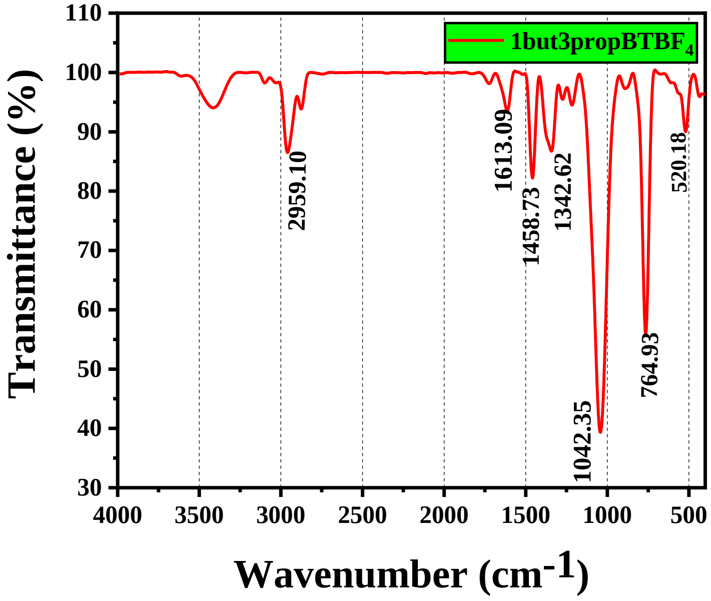

Figure S58. 1but3propBTBF_4

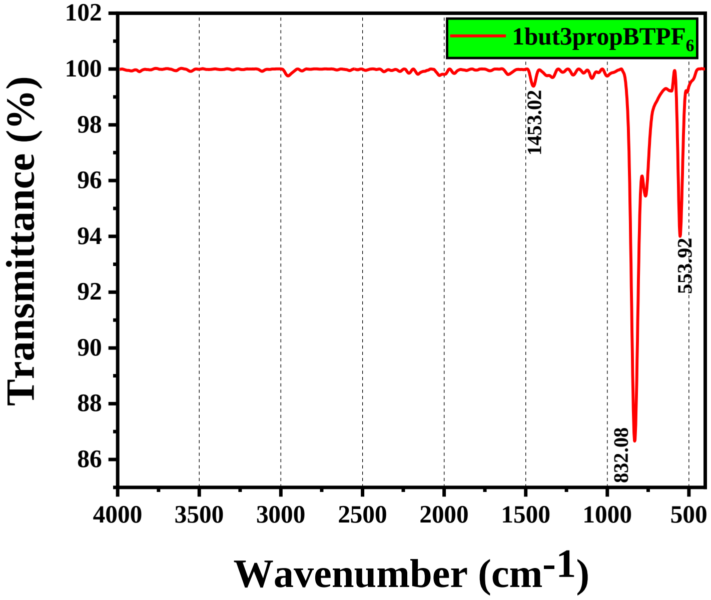

Figure S59. 1but3propBTPF_6

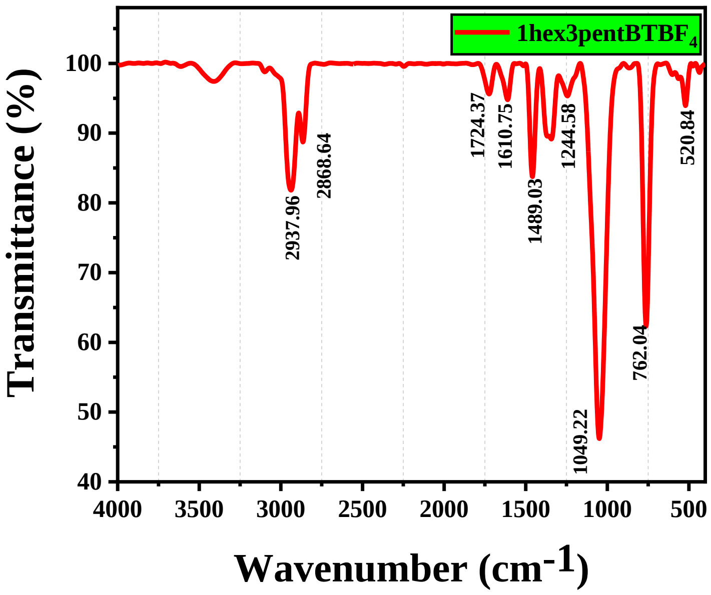

Figure S60. 1hex3pentBTBF$_4$

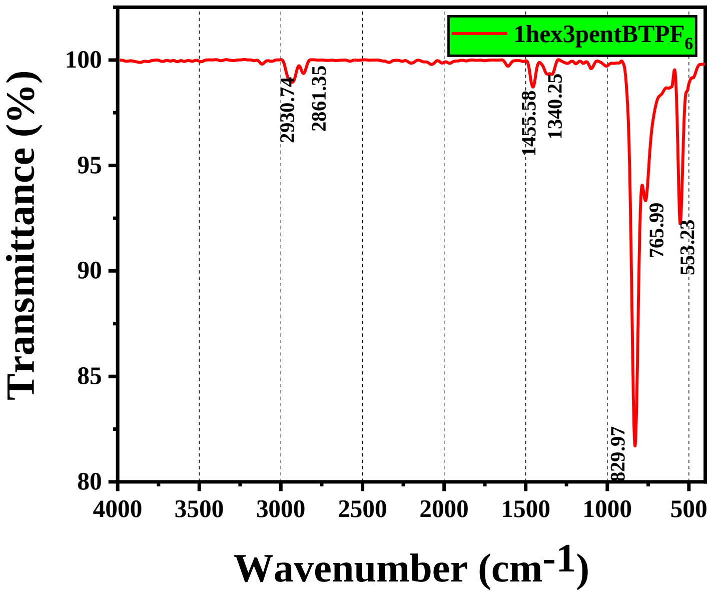

Figure S61. 1hex3pentBTPF$_6$

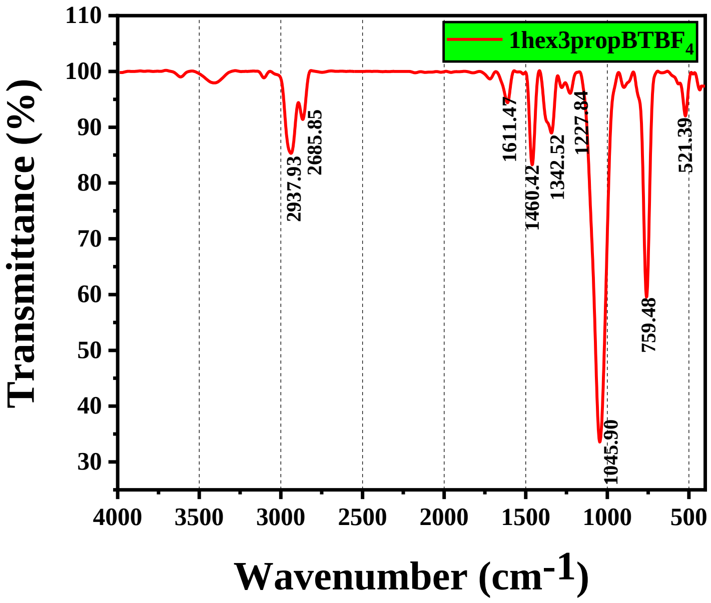

Figure S62. 1hex3propBTBF$_4$

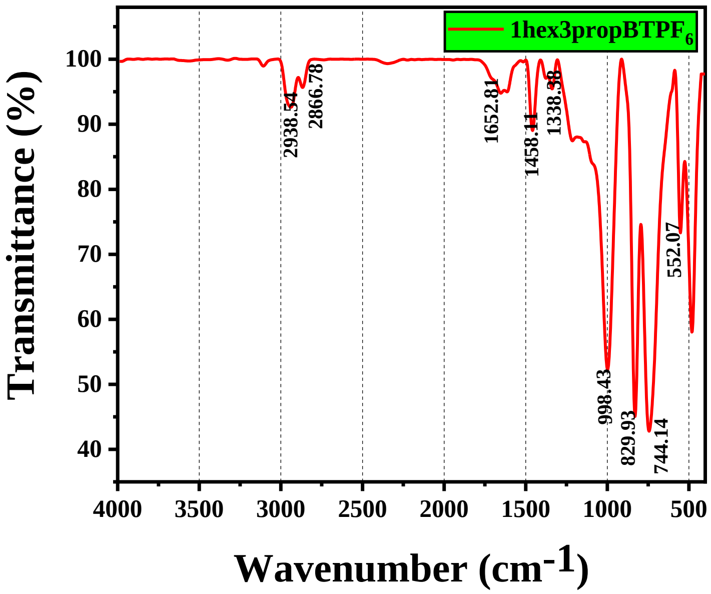

Figure S63. 1hex3pentBTPF$_6$

S4. EIS

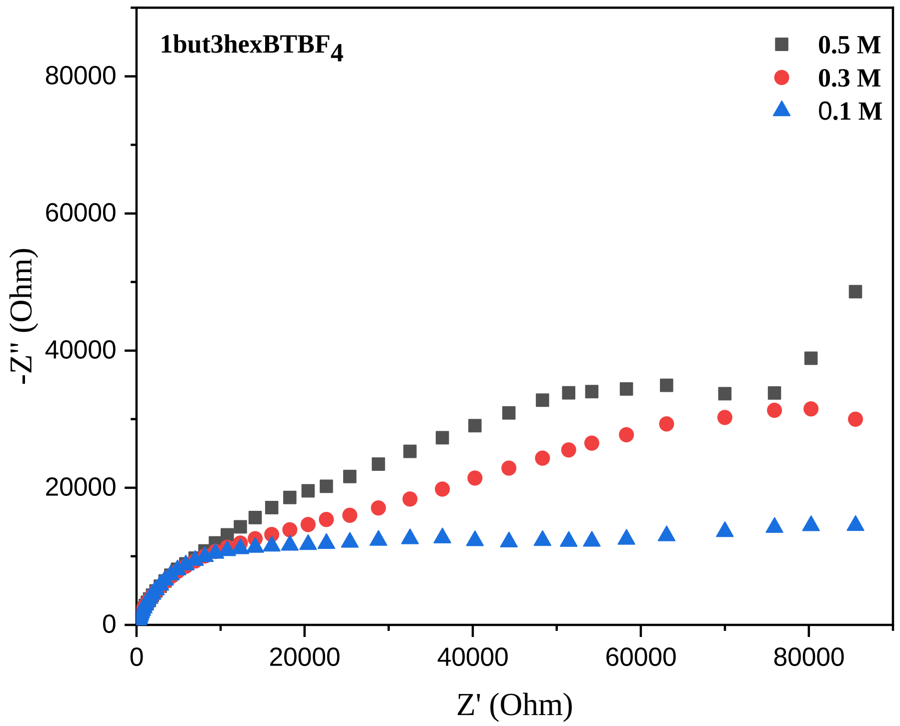

Figure S64. Impedance graph of 1but3hexBTBF$_4$ with 3 different concentration 0.5, 0.3,and 0.1 M

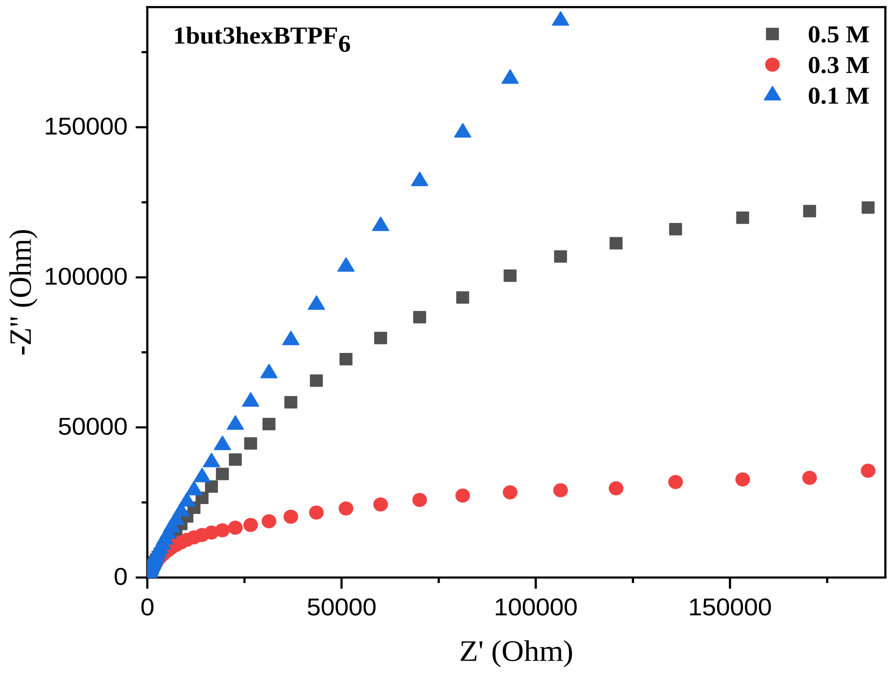

Figure S65. Impedance graph of 1but3hexBTPF6 with 3 different concentration 0.5, 0.3,and 0.1 M

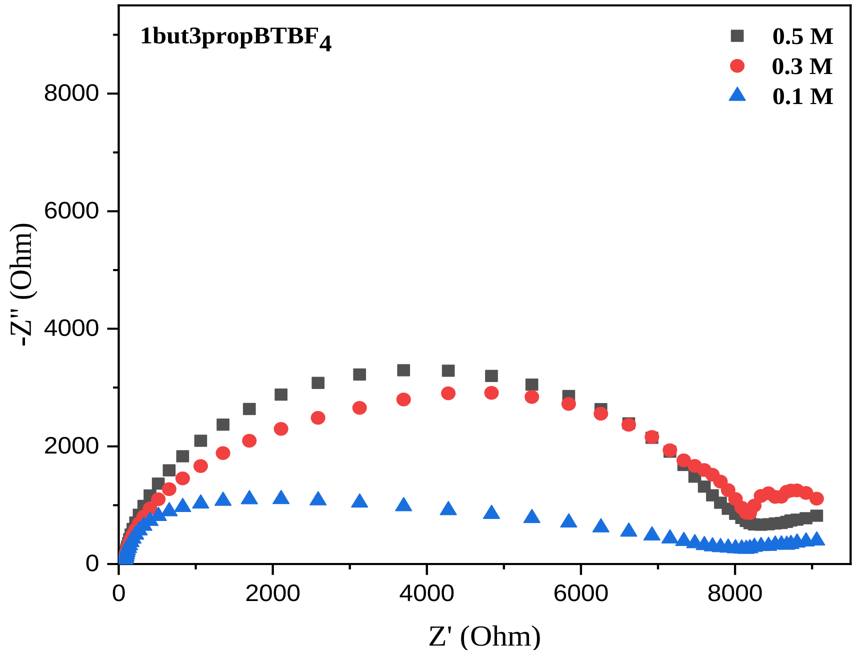

Figure S66. Impedance graph of 1but3propBTBF4 with 3 different concentration 0.5, 0.3,and 0.1 M

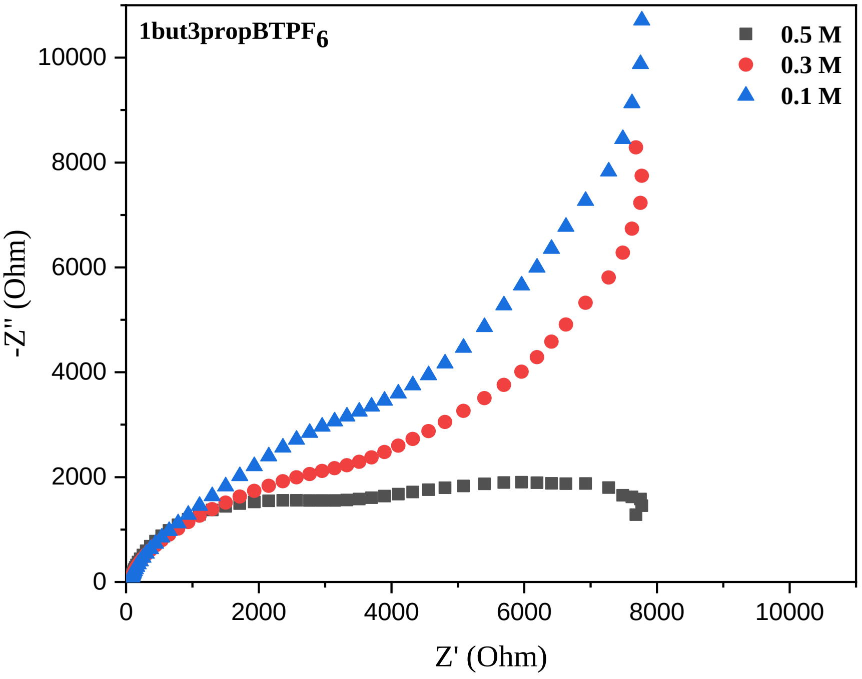

Figure S67. Impedance graph of 1but3propBTPF6 with 3 different concentration 0.5, 0.3,and 0.1 M

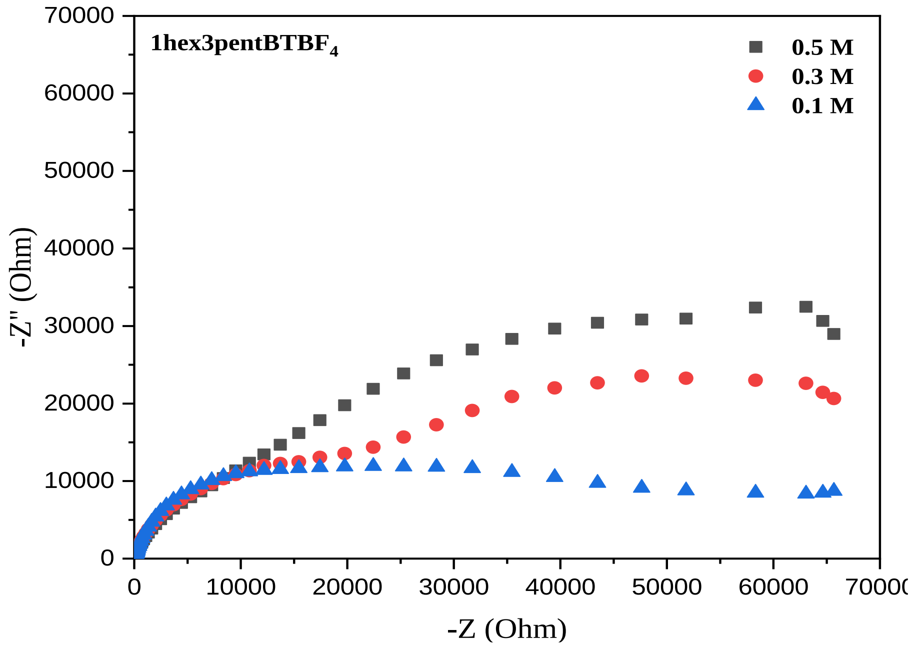

Figure S68. Impedance graph of 1hex3pentBTBF$_4$ with 3 different concentration 0.5, 0.3,and 0.1 M

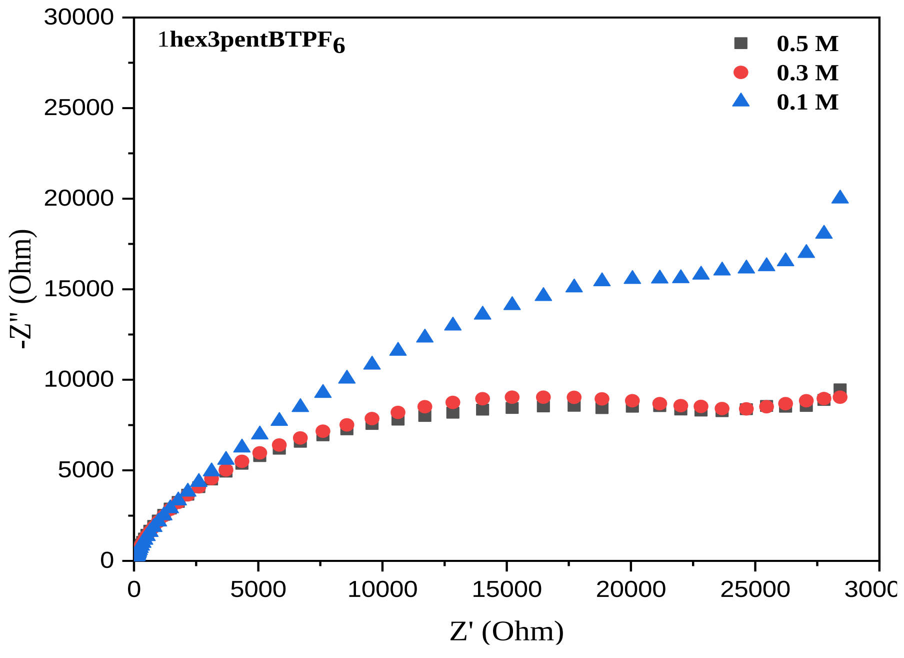

Figure S69. Impedance graph of 1hex3pentBTPF$_6$ with 3 different concentration 0.5, 0.3,and 0.1 M

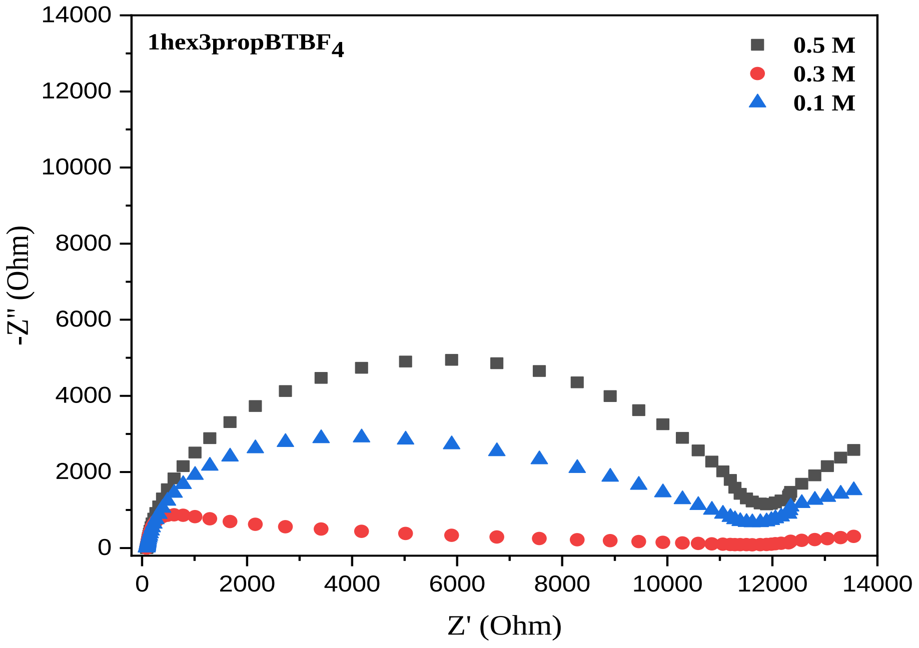

Figure S70. Impedance graph of 1hex3propBTBF$_4$ with 3 different concentration 0.5, 0.3,and 0.1 M

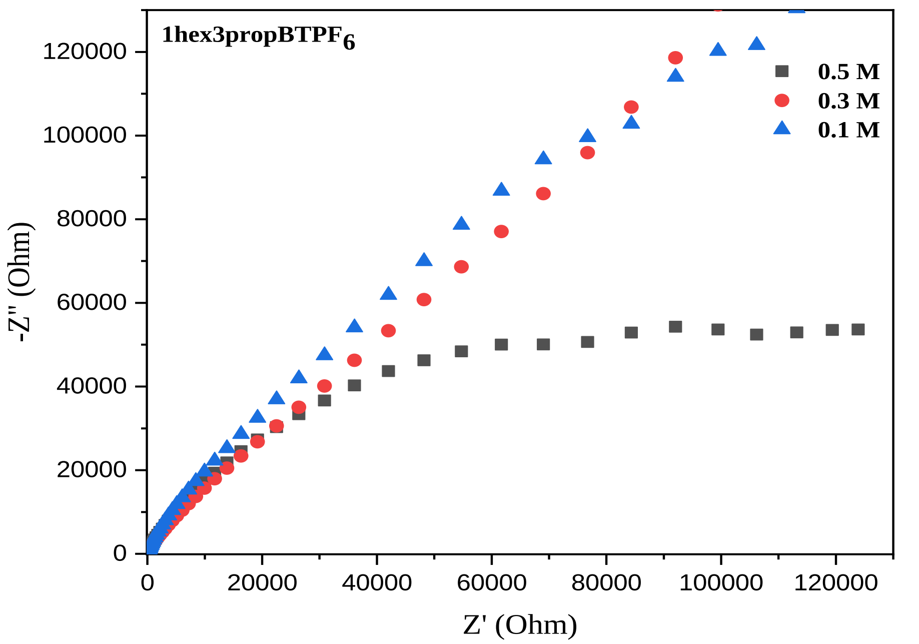

Figure S71. Impedance graph of 1hex3propBTPF$_6$ with 3 different concentration 0.5, 0.3,and 0.1 M

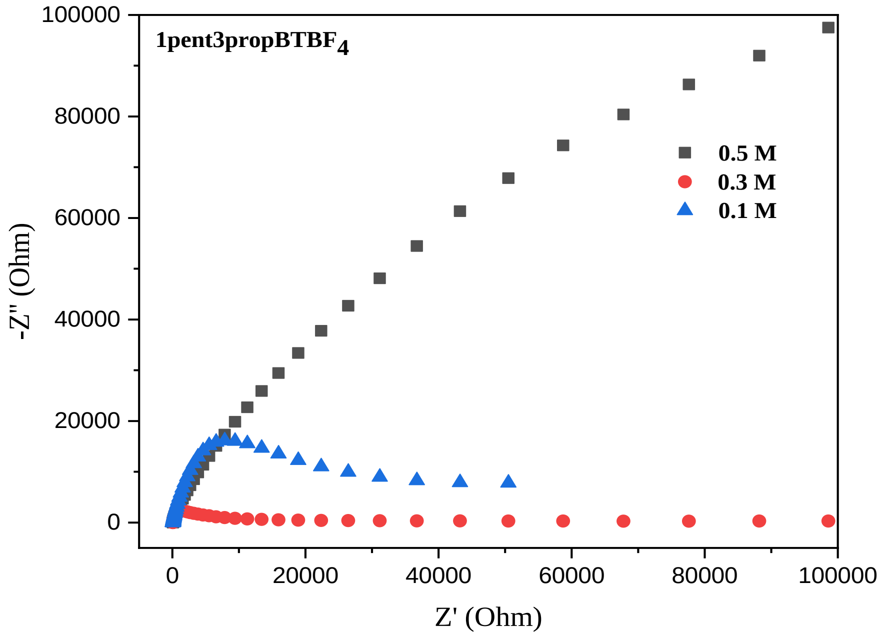

Figure S72. Impedance graph of 1pent3propBTBF_4 with 3 different concentration 0.5, 0.3,and 0.1 M

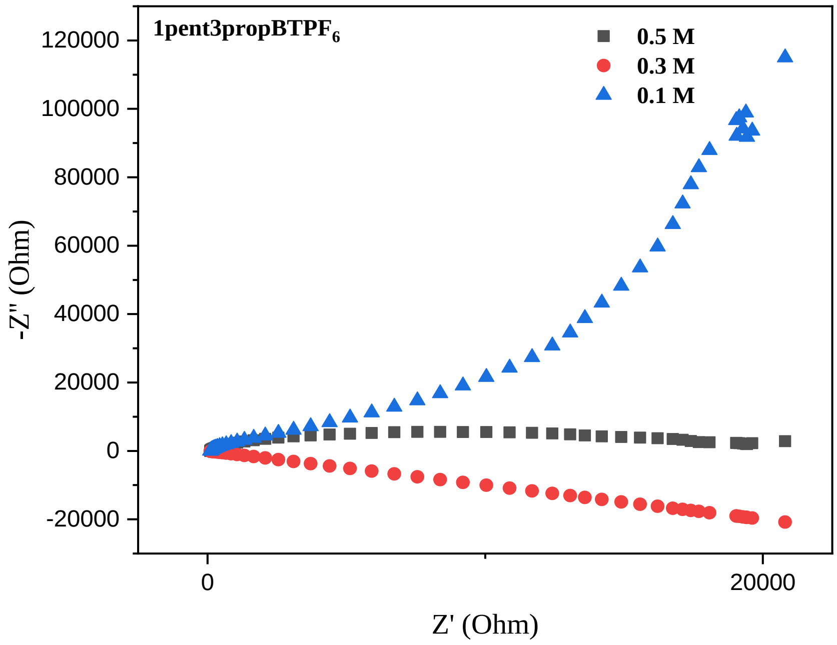

Figure S73. Impedance graph of 1pent3propBTPF_6 with 3 different concentration 0.5, 0.3,and 0.1 M

Table S2. Calculated resistance and conductance variation with different concentrations from EIS graphs

Compound	Conc. (M)	R_s (Ω)	Q_{dl}	R_{ct} (Ω)	R_W (S s0.5)	C_f	R_f (Ω)	Conductance (Ω^{-1})
1but3propBTBF$_4$	0.5	170.8	0.0001888	5.63E+01	0.0001077	7.84E-07	5180	0.017761989
	0.3	89.01	1.59E-06	2677	0.002039	0.0006506	274.1	0.000373552
	0.1	72.19	1.44E-06	6331	0.000749	1.54E-06	1553	0.00014637
1but3propBTPF$_6$	0.5	67.98	2.01E-06	3359	0.0004473	2.68E-05	2867	0.000297708
	0.3	89.62	2.51E-06	3690	7.95E-05	6.25E-05	1178	0.000271003
	0.1	190.6	6.01E-06	6810	5.67E-05	3.44E-06	1201	0.000146843
1hex3propBTBF$_4$	0.5	107.1	8.94E-06	799.9	0.002167	4.00E-07	1328	0.001250156
	0.3	232.1	1.01E-05	1891	0.0004404	5.19E-07	4742	0.000528821
	0.1	90.27	4.90E-07	1.10E+04	0.000286	1.55E-06	2.128	9.10E-05
1hex3propBTPF$_6$	0.5	112.3	1.05E-06	1.19E+04	2.88E-06	2.96E-06	804.1	8.38E-05
	0.3	237.1	6.68E-07	1.26E+04	5.44E-06	3.52E-06	8.08E+04	7.94E-05
	0.1	85.27	1.45E-06	1.01E+05	1.70E-05	1.17E-06	2575	9.90E-06
1but3hexBTBF$_4$	0.5	87.68	8.91E-07	1.48E+04	1.67E-05	4.48E-06	2.72E+04	6.76E-05
	0.3	99.75	9.00E-07	1.55E+04	3.58E-05	9.34E-06	1.83E+04	6.45E-05
	0.1	223.5	9.23E-07	1.98E+04	4.92E-05	8.25E-06	1.17E+04	5.06E-05
1but3hexBTPF$_6$	0.5	88.56	6.24E-07	4069	5.92E-06	3.23E-06	7.92E+04	0.000245761
	0.3	93.56	8.65E-07	7246.37	3.78E-06	2.22E-06	3.01E+04	0.000137893
	0.1	239.7	1.21E-06	2.21E+05	1.95E-06	1.49E-06	4101	4.52E-06
1hex3pentBTBF$_4$	0.5	88.65	1.51E-06	1.33E+04	2.85E-05	6.36E-06	3.34E+04	7.55E-05
	0.3	111.7	1.33E-06	1.74E+04	5.68E-05	9.33E-06	2.95E+04	5.74E-05

	0.1	227.7	1.23E-06	1.84E+04	9.55E-05	7.11E-06	1.34E+04	5.44E-05
1hex3pentBTPF$_6$	0.5	76.95	1.36E-06	8736	7.09E-05	5.65E-06	1.05E+04	0.000114469
	0.3	99.41	1.51E-06	9857	7.53E-05	6.04E-06	1.11E+04	0.000101451
	0.1	209.7	1.98E-06	2.94E+04	3.41E-05	0.7771	0.8198	3.40E-05
1but3pentBTBF$_4$	0.5	122.9	0.0002705	97.23	0.01849	2.96E-07	142.7	0.010284891
	0.3	235.5	2.06E-05	250.2	0.006559	3.99E-07	283	0.003996803
	0.1	98.91	4.83E-07	4634	5.14E+12	0.0001403	339.2	0.000215796
1but3pentBTPF$_6$	0.5	173.8	0.000399	210.7	5.19E-05	2.99E-07	3508	0.004746084
	0.3	137.4	5.98E-06	2500	0.001629	4.23E-07	2958	0.0004
	0.1	99.53	1.63E-06	1.69E+04	0.0003333	7.14E-07	4080	5.91E-05
1pent3propBTBF$_4$	0.5	127.3	3.44E-06	3316	0.001627	4.98E-07	2965	0.000301568
	0.3	95.24	9.97E-07	1.83E+04	5.50E-06	2.99E-06	6.79E+04	5.46E-05
	0.1	88.67	8.65E-06	2.63E+04	4.90E-06	4.45E-06	7.99E+04	3.79E-05
1pent3propBTPF$_6$	0.5	102.4	6.19E-07	9261	0.0002605	3.83E-06	7589	0.00010798
	0.3	147.3	2.64E-06	1.18E+04	0.0002172	4.89E-07	4842	8.45E-05
	0.1	418.8	1.27E-06	1.77E+05	9.95E-06	1.12E-06	8254	5.64E-06

S5. Cyclic Voltammogram

Cyclic voltammograms of asymmetrically substituted 1,3-dialkyl-1,2,3-benzotriazolium salts with 0.5, 0.3, and 0.1 M concentrations in acetonitrile recorded at different scan rates, with platinum disc as working electrode and Pt wire as counter and reference electrodes.

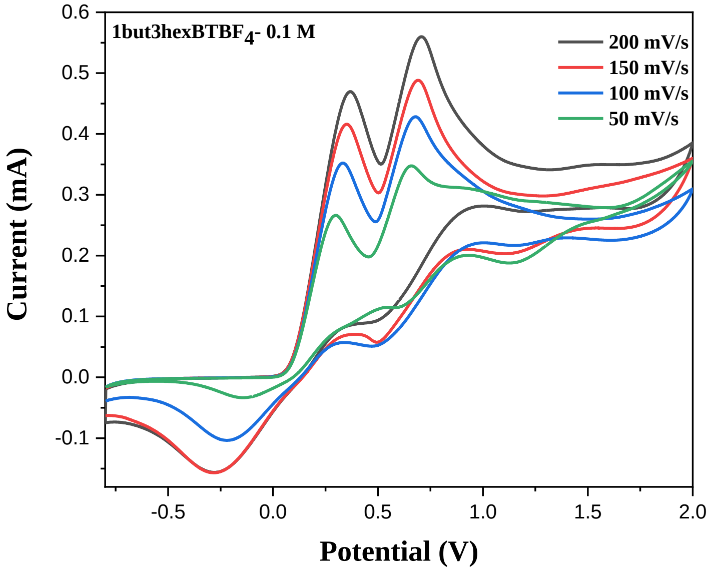

Figure S74. Cyclic Voltammogram of 1but3hexBTBF$_4$ with concentration 0.1 M and 4 different scan rates 200 mV/s, 150 mV/s, 100 mV/s and 50 mV/s

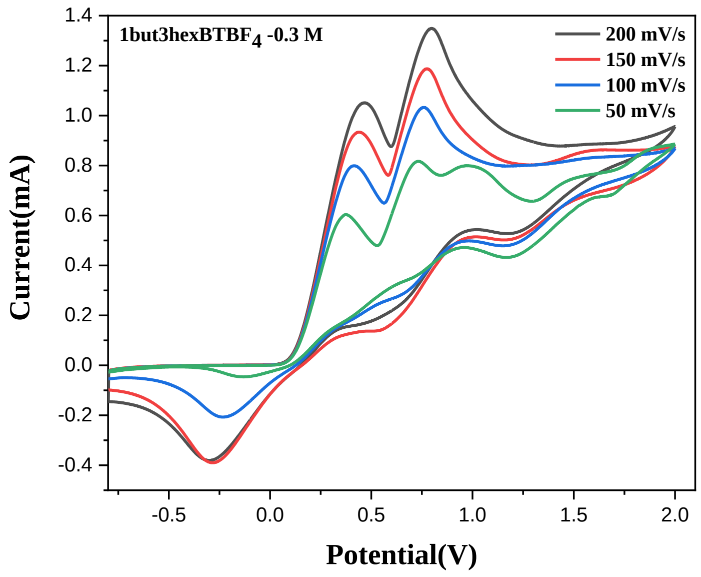

Figure S75. Cyclic Voltammogram of 1but3hexBTBF$_4$ with concentration 0.3 M and 4 different scan rates 200 mV/s, 150 mV/s, 100 mV/s and 50 mV/s

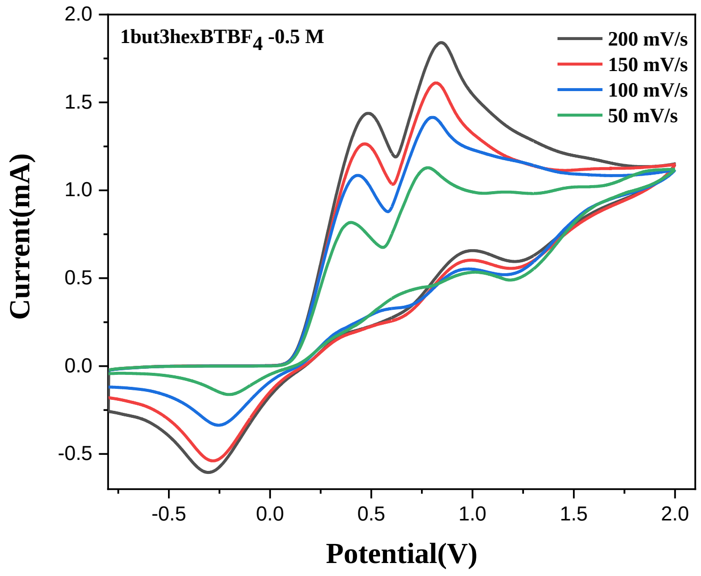

Figure S76. Cyclic Voltammogram of 1but3hexBTBF$_4$ with concentration 0.5 M and 4 different scan rates 200 mV/s, 150 mV/s, 100 mV/s and 50 mV/s

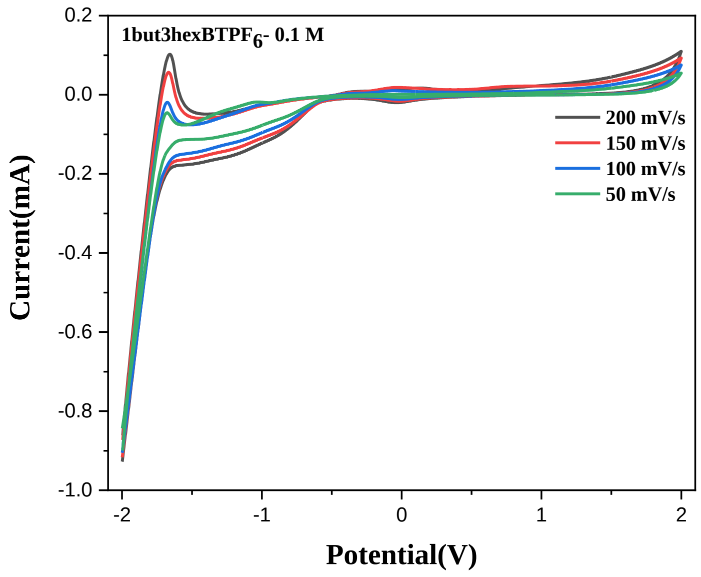

Figure S77. Cyclic Voltammogram of 1but3hexBTPF$_6$ with concentration 0.1 M and 4 different scan rates 200 mV/s, 150 mV/s, 100 mV/s and 50 mV/s

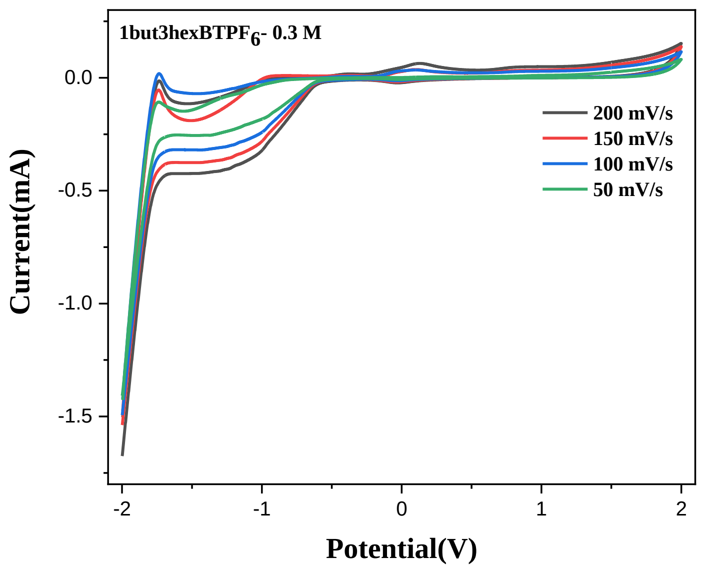

Figure S78. Cyclic Voltammogram of 1 but3hexBTPF$_6$ with concentration 0.3 M and 4 different scan rates 200 mV/s, 150 mV/s, 100 mV/s and 50 mV/s

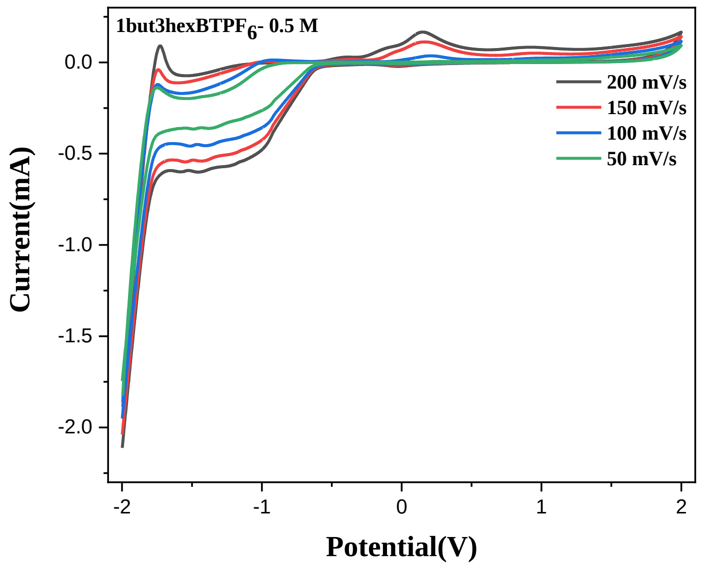

Figure S79. Voltammogram of 1but3hexBTPF_6 with concentration 0.5 M and 4 different scan rates 200 mV/s, 150 mV/s, 100 mV/s and 50 mV/s

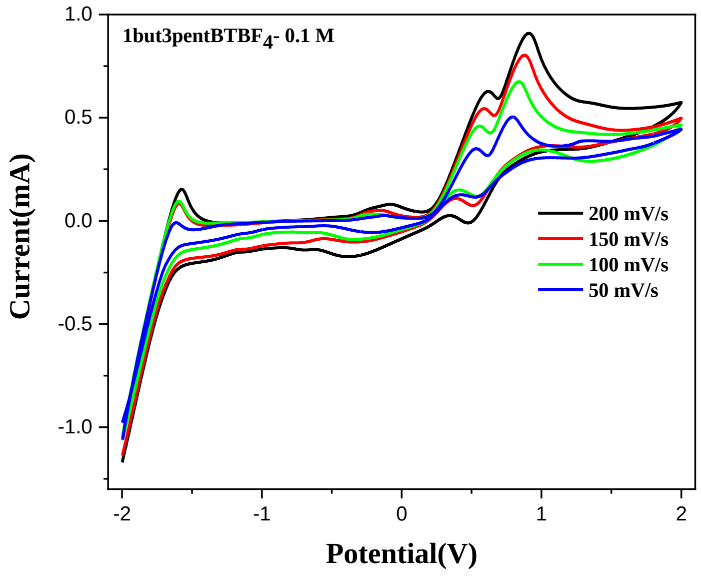

Figure S80. Cyclic Voltammogram of 1but3pentBTBF_4 with concentration 0.1 M and 4 different scan rates 200 mV/s, 150 mV/s, 100 mV/s and 50 mV/s

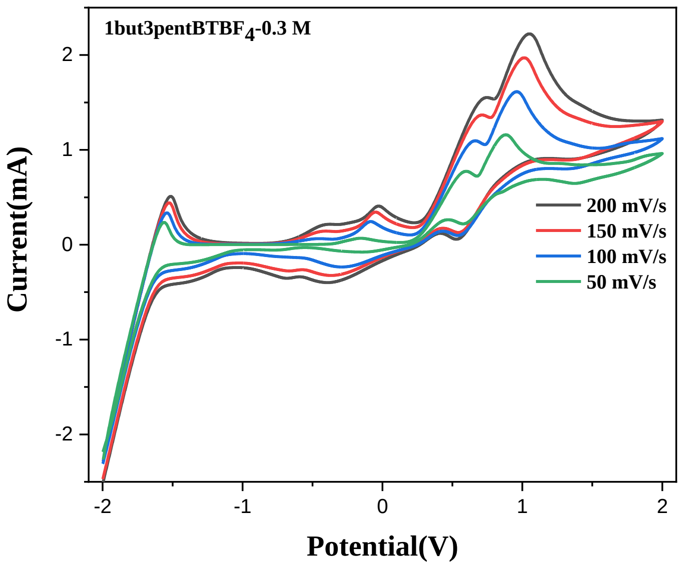

Figure S81. Cyclic Voltammogram of 1but3pentBTBF$_4$ with concentration 0.3 M and 4 different scan rates 200 mV/s, 150 mV/s, 100 mV/s and 50 mV/s

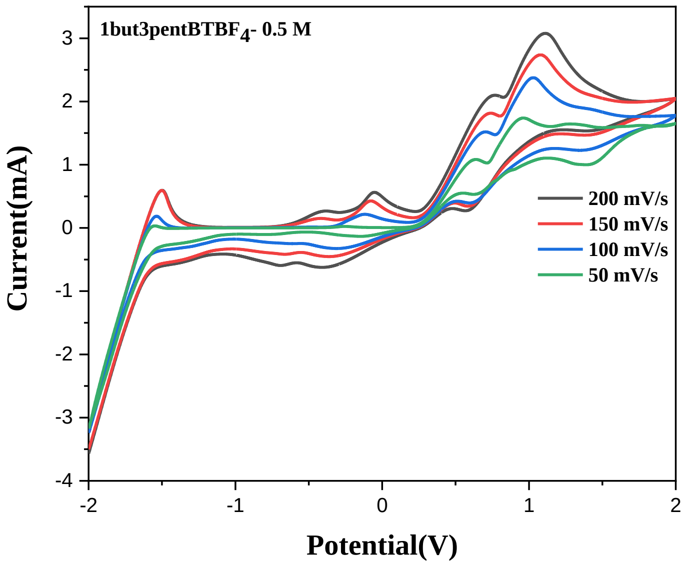

Figure S82. Cyclic Voltammogram of 1but3pentBTBF$_4$ with concentration 0.5 M and 4 different scan rates 200 mV/s, 150 mV/s, 100 mV/s and 50 mV/s

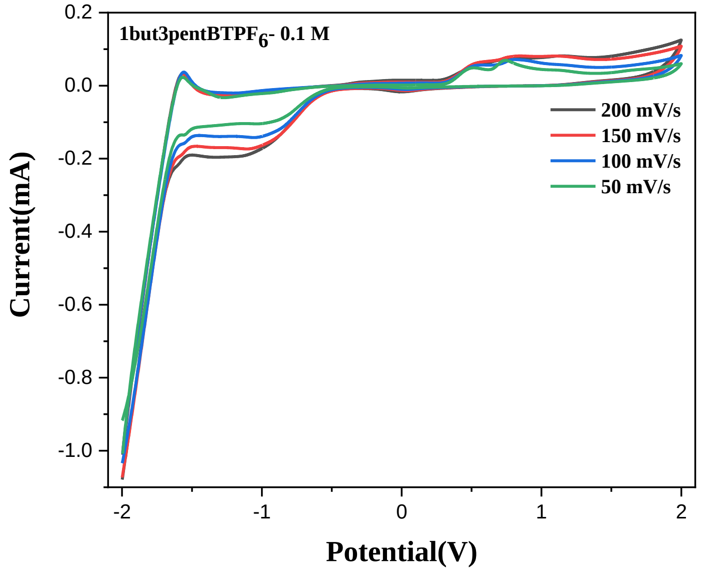

Figure S83. Cyclic Voltammogram of 1 but3pentBTPF$_6$ with concentration 0.1 M and 4 different scan rates 200 mV/s, 150 mV/s, 100 mV/s and 50 mV/s

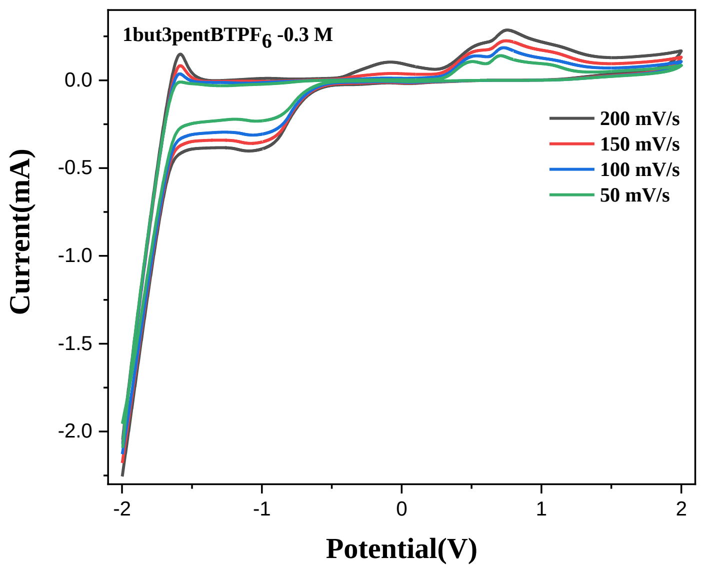

Figure S84. Cyclic Voltammogram of 1but3pentBTPF$_6$ with concentration 0.3 M and 4 different scan rates 200 mV/s, 150 mV/s, 100 mV/s and 50 mV/s

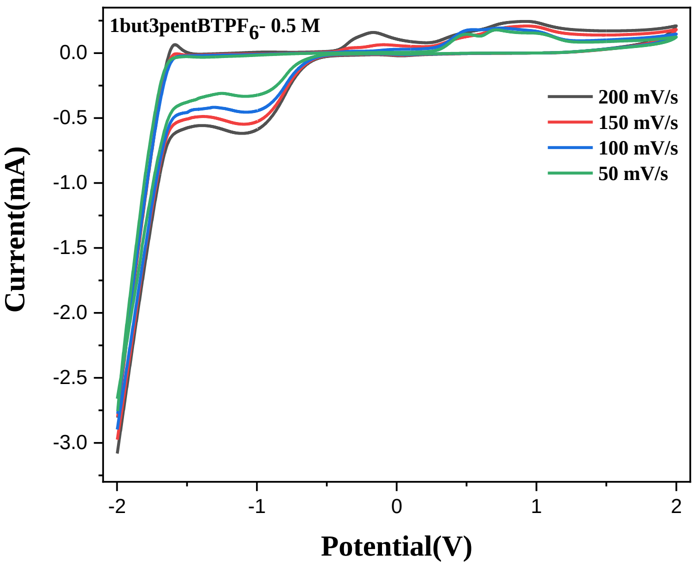

Figure S85. Cyclic Voltammogram of 1but3pentBTPF$_6$ with concentration 0.5 M and 4 different scan rates 200 mV/s, 150 mV/s, 100 mV/s and 50 mV/s

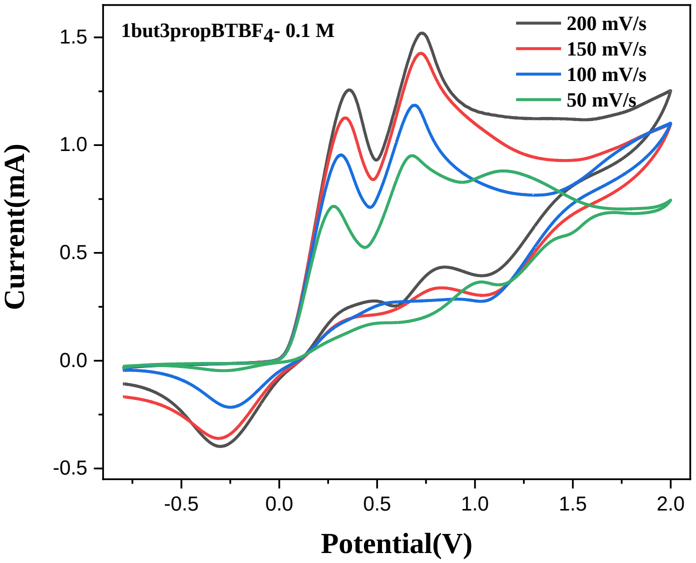

Figure S86. Cyclic Voltammogram of 1but3propBTBF$_4$ with concentration 0.1 M and 4 different scan rates 200 mV/s, 150 mV/s, 100 mV/s and 50 mV/s

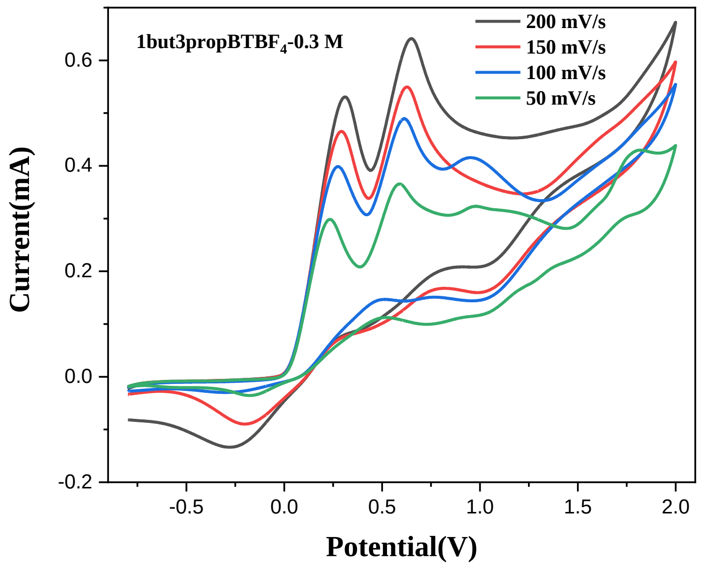

Figure S87. Cyclic Voltammogram of 1but3propBTBF$_4$ with concentration 0.3 M and 4 different scan rates 200 mV/s, 150 mV/s, 100 mV/s and 50 mV/s

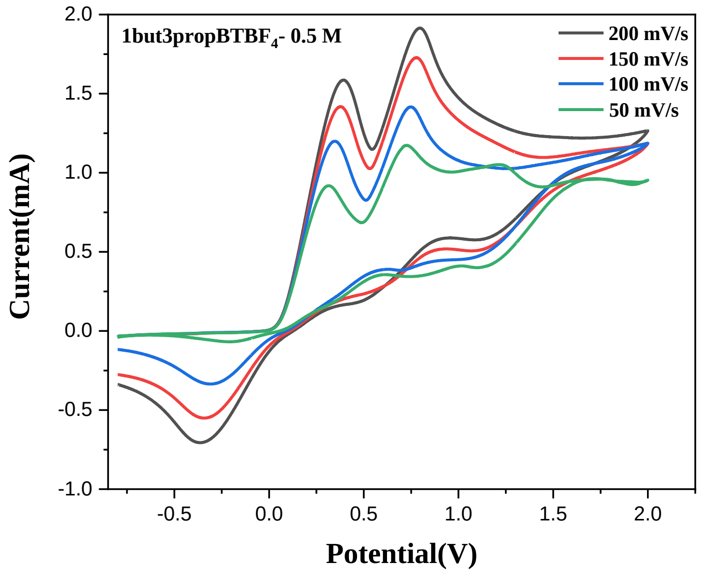

Figure S88. Cyclic Voltammogram of 1but3propBTBF$_4$ with concentration 0.5 M and 4 different scan rates 200 mV/s, 150 mV/s, 100 mV/s and 50 mV/s

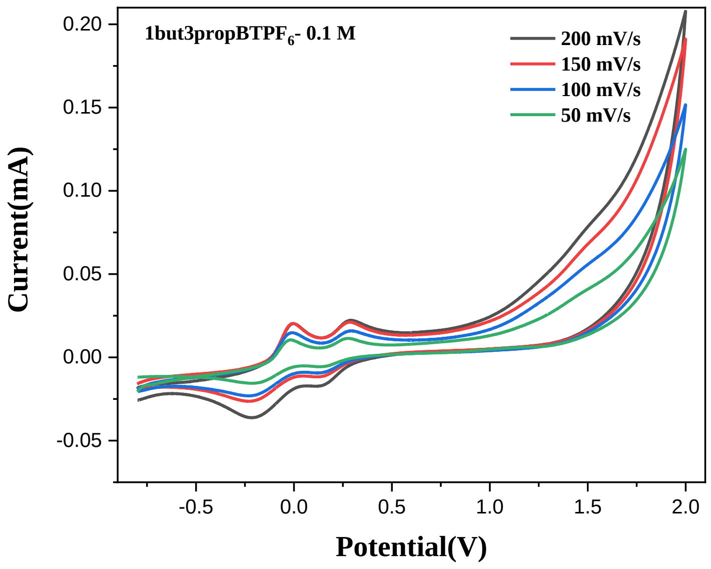

Figure S89. Cyclic Voltammogramof 1but3propBTPF$_6$ with concentration 0.1 M and 4 different scan rates 200 mV/s, 150 mV/s, 100 mV/s and 50 mV/s

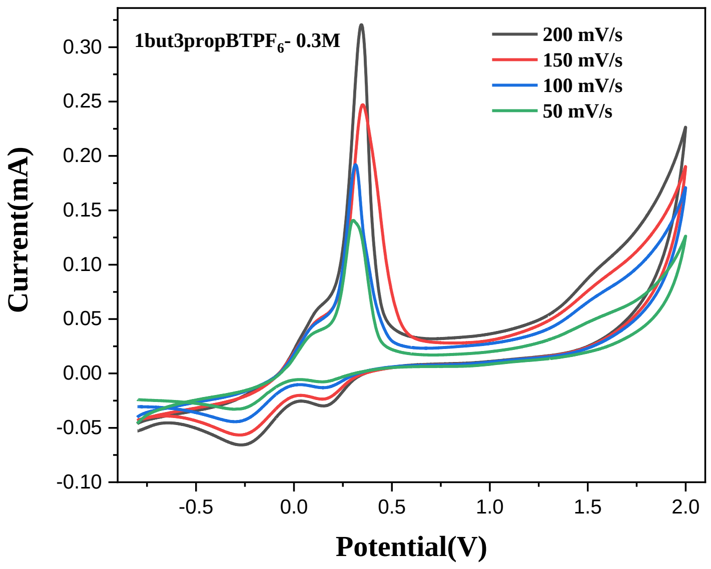

Figure S90. Cyclic Voltammogramof 1but3propBTPF$_6$ with concentration 0.3 M and 4 different scan rates 200 mV/s, 150 mV/s, 100 mV/s and 50 mV/s

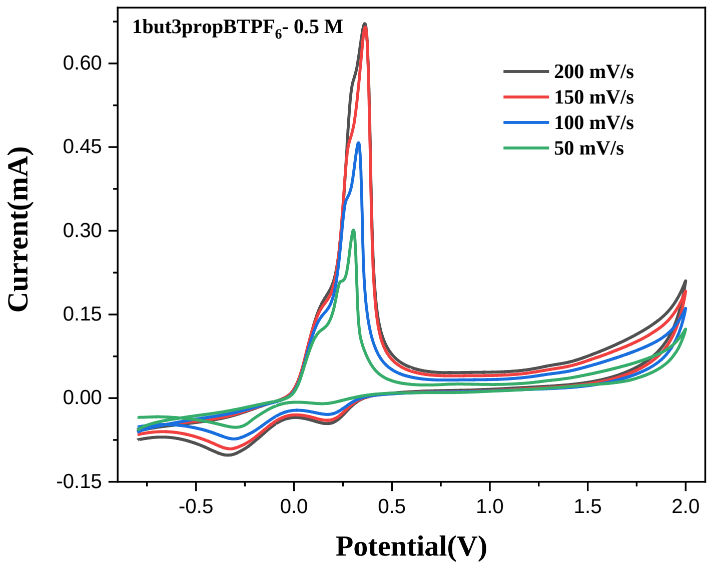

Figure S91. Cyclic Voltammogram of 1 but3propBTPF$_6$ with concentration 0.5 M and 4 different scan rates 200 mV/s, 150 mV/s, 100 mV/s and 50 mV/s

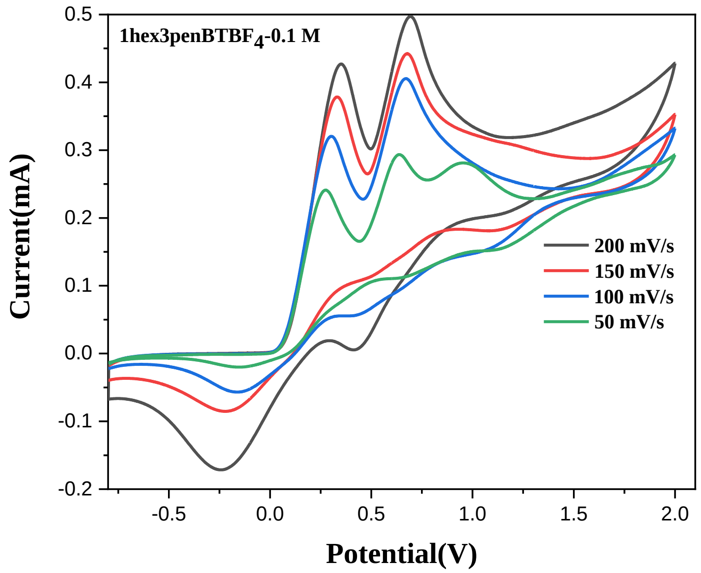

Figure S92. Cyclic Voltammogram of 1hex3pentBTBF$_4$ with concentration 0.1 M and 4 different scan rates 200 mV/s, 150 mV/s, 100 mV/s and 50 mV/s

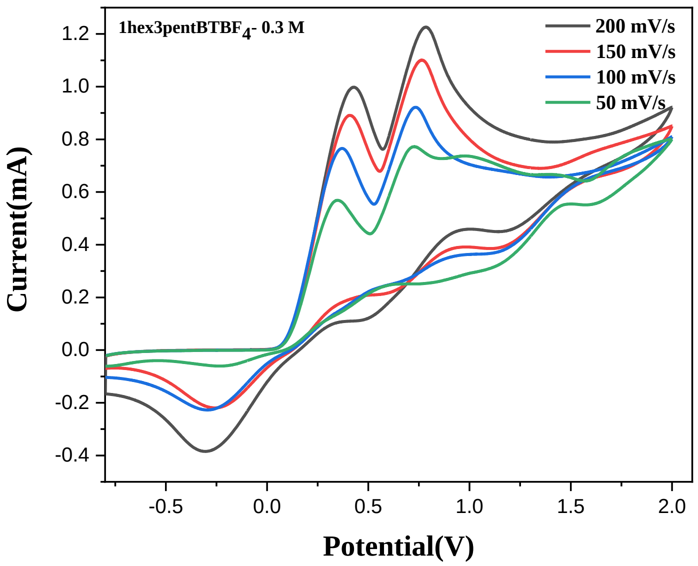

Figure S93. Cyclic Voltammogram of 1hex3pentBTBF$_4$ with concentration 0.3 M and 4 different scan rates 200 mV/s, 150 mV/s, 100 mV/s and 50 mV/s

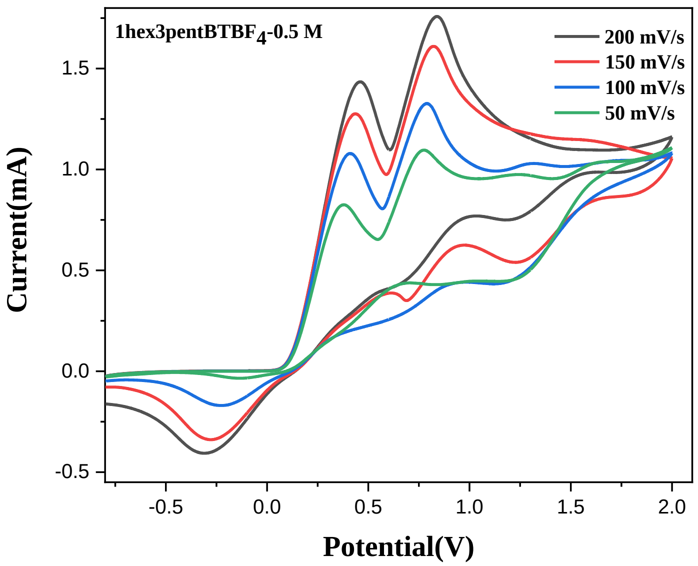

Figure S94. Cyclic Voltammogram of 1hex3pentBTBF$_4$ with concentration 0.5 M and 4 different scan rates 200 mV/s, 150 mV/s, 100 mV/s and 50 mV/s

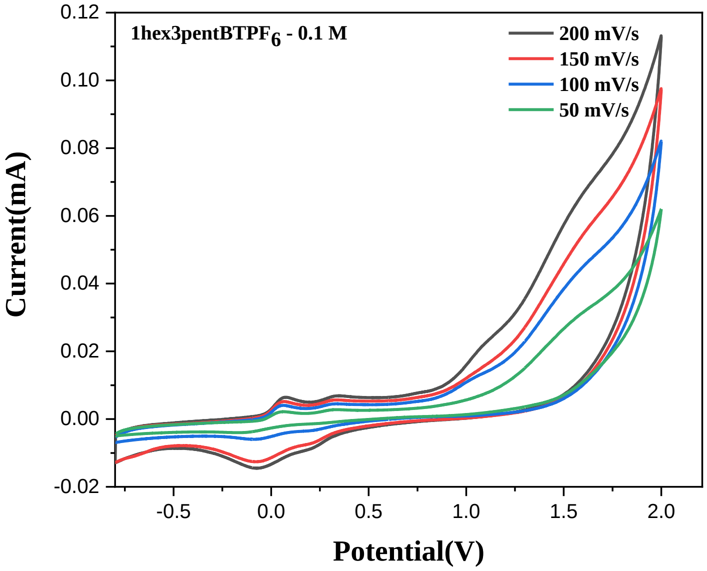

Figure S95. Cyclic Voltammogram of 1hex3pentBTPF$_6$ with concentration 0.1 M and 4 different scan rates 200 mV/s, 150 mV/s, 100 mV/s and 50 mV/s

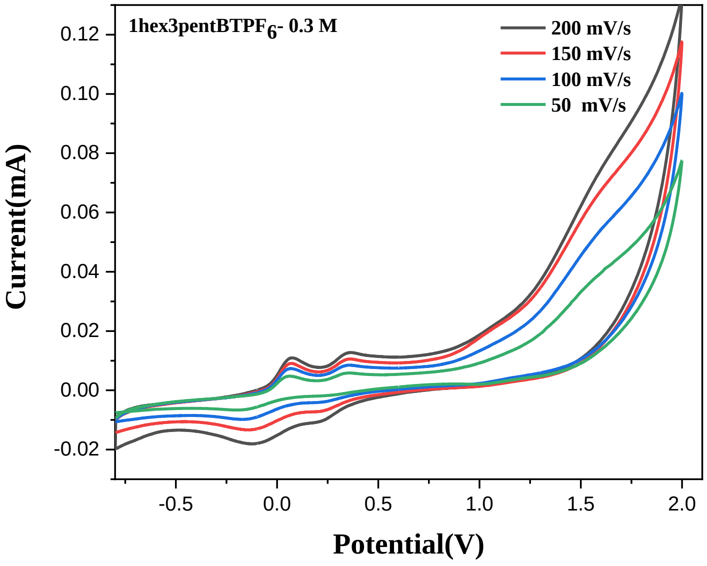

Figure S96. Cyclic Voltammogram of 1hex3pentBTPF$_6$ with concentration 0.3 M and 4 different scan rates 200 mV/s, 150 mV/s, 100 mV/s and 50 mV/s

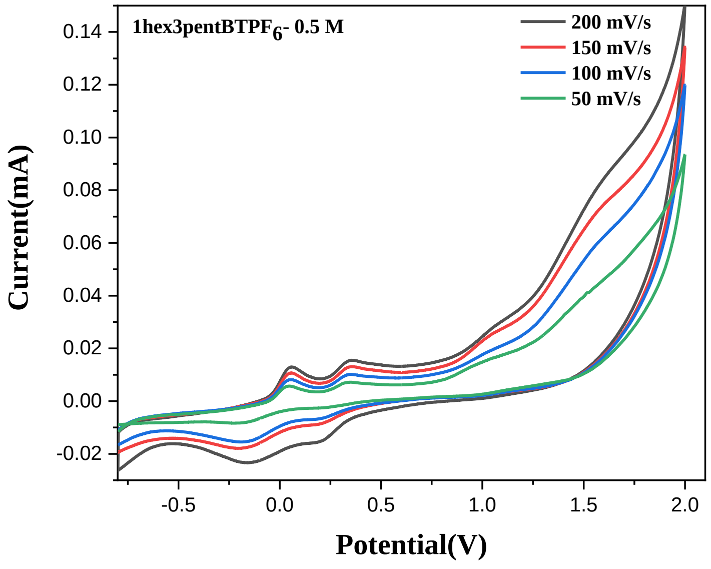

Figure S97. Cyclic Voltammogram of 1hex3pentBTPF$_6$ with concentration 0.5 M and 4 different scan rates 200 mV/s, 150 mV/s, 100 mV/s and 50 mV/s

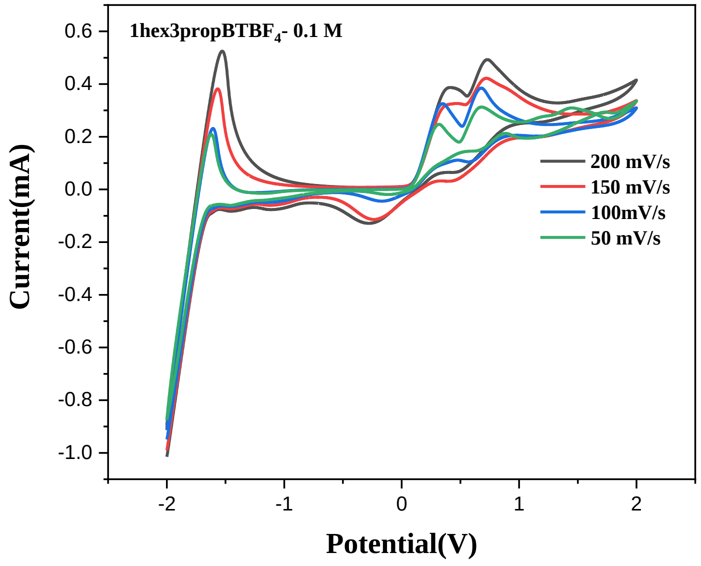

Figure S98. Cyclic Voltammogram of 1hex3propBTBF$_4$ with concentration 0.1 M and 4 different scan rates 200 mV/s, 150 mV/s, 100 mV/s and 50 mV/s

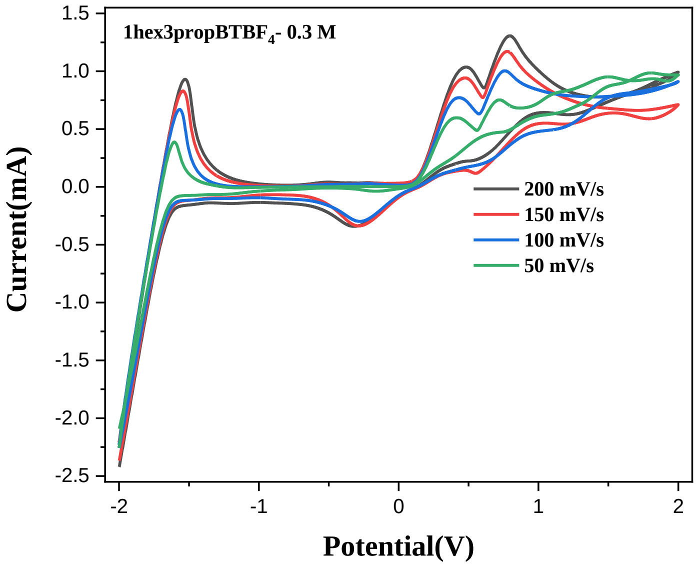

Figure S99. Cyclic Voltammogram of 1hex3propBTBF$_4$ with concentration 0.3 M and 4 different scan rates 200 mV/s, 150 mV/s, 100 mV/s and 50 mV/s

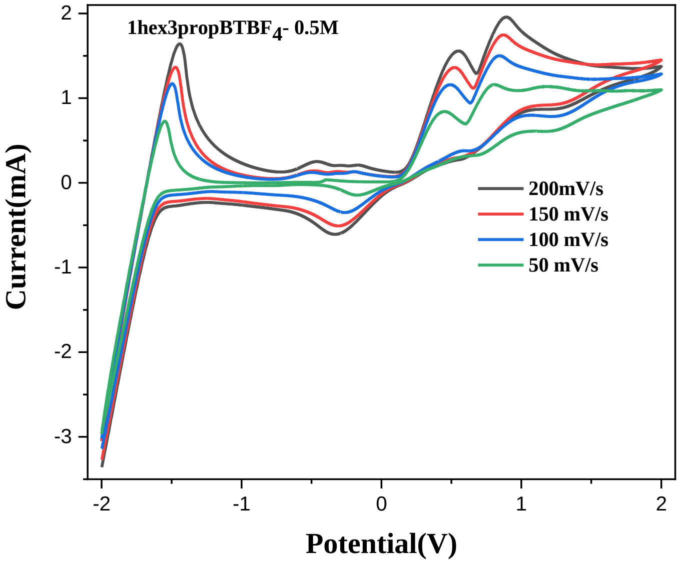

Figure S100. Cyclic Voltammogram of 1hex3propBTBF$_4$ with concentration 0.5 M and 4 different scan rates 200 mV/s, 150 mV/s, 100 mV/s and 50 mV/s

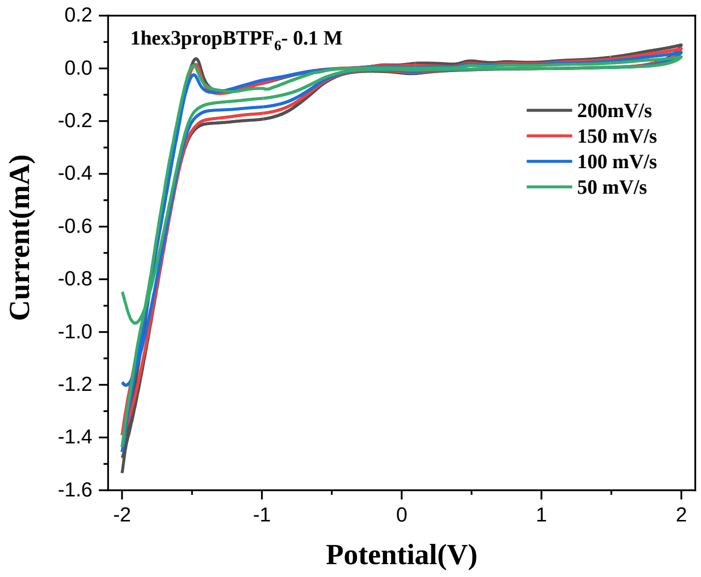

Figure S101. Cyclic Voltammogram of 1hex3propBTPF$_6$ with concentration 0.1 M and 4 different scan rates 200 mV/s, 150 mV/s, 100 mV/s and 50 mV/s

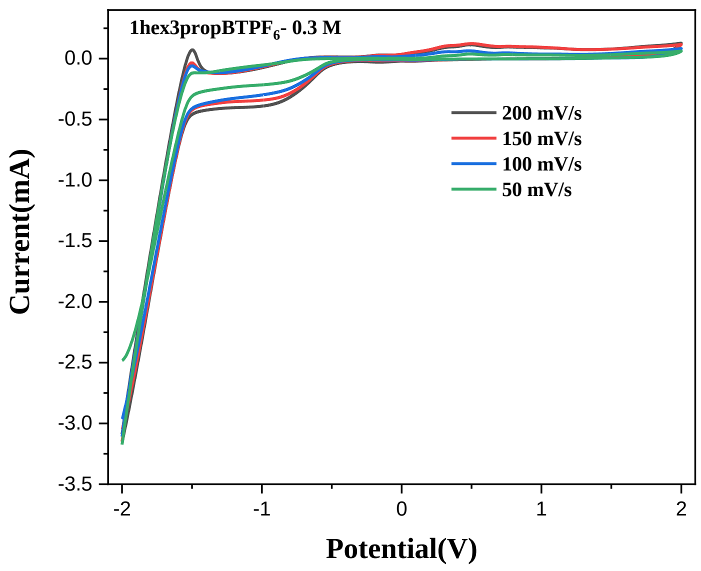

Figure S102. Cyclic Voltammogram of 1hex3propBTPF$_6$ with concentration 0.3 M and 4 different scan rates 200 mV/s, 150 mV/s, 100 mV/s and 50 mV/s

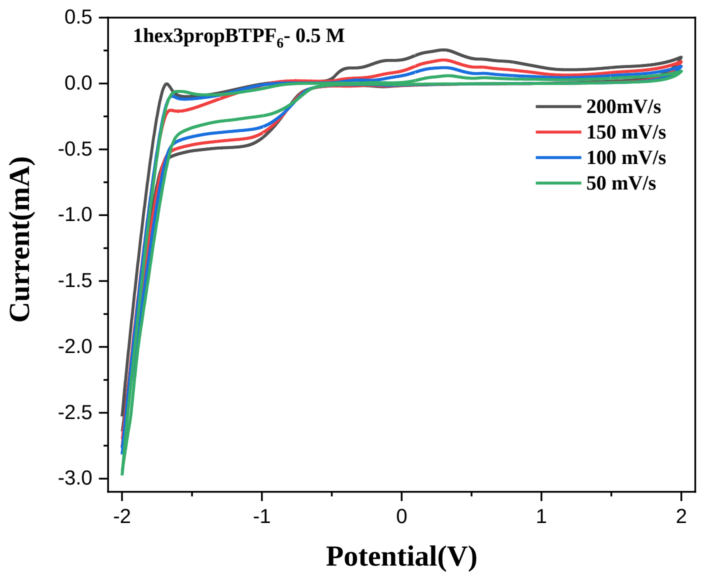

Figure S103. Cyclic Voltammogram of 1hex3propBTPF_6 with concentration 0.5 M and 4 different scan rates 200 mV/s, 150 mV/s, 100 mV/s and 50 mV/s

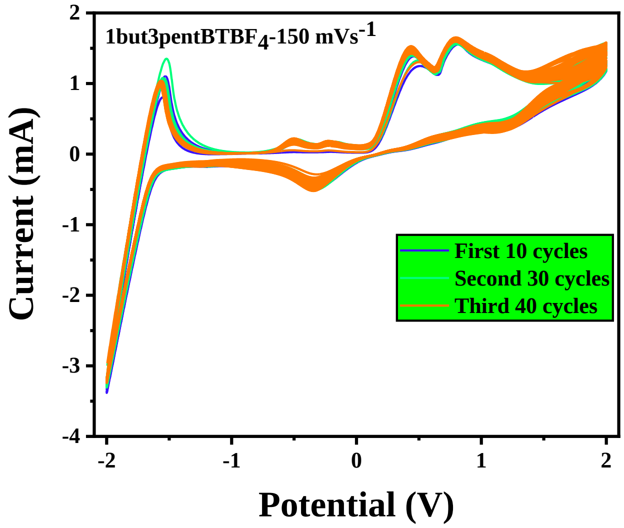

Figure S104. Cyclic stability of the asymmetrically substituted 1-butyl-3-pentyl-1,2,3-benzotriazolium tetrafluoroborate in acetonitrile solution of 0.5 M concentration with cyclic voltammetry recorded with platinum disc as working electrode and platinum wire as counter and reference electrodes, at a scan rate of 150 mV/s

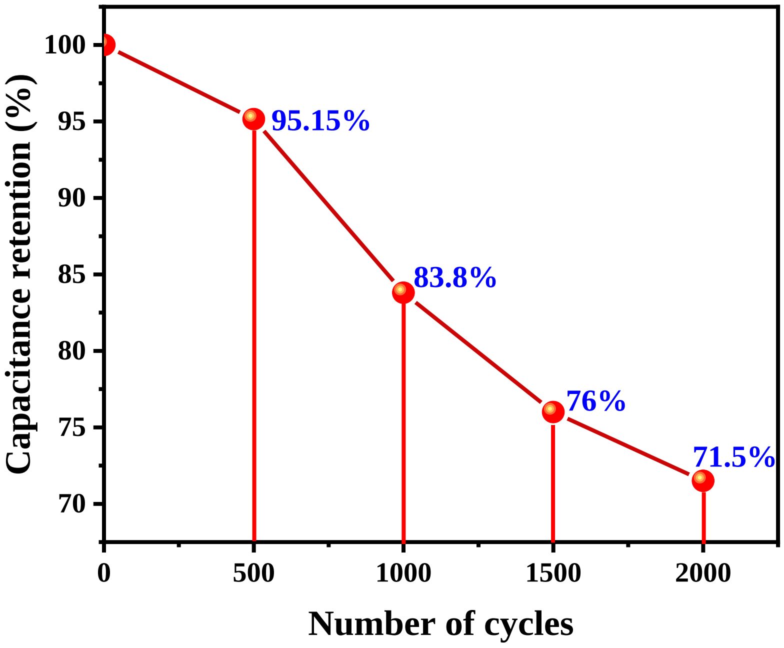

Figure S105. % capacitance retention with varying cycles

S6. Equations for calculating supercapacitor performance

$$\text{Specific capacitance (CV)} = \frac{\int \mathrm{I\,dV}}{2\Delta \mathrm{V}\, v\, \mathrm{m}} \quad \text{........ (4)}$$

$$\text{Specific capacitance (GCD)} = \frac{I X \Delta\, t}{\Delta V\ X\ m} \quad \text{........... (5)}$$

$$\mathrm{E} = \frac{\mathrm{C\ X\ V^2}}{7200} \quad \text{............... (6)}$$

$$\mathrm{P} = \frac{\mathrm{E}}{\Delta \mathrm{t}}\ \mathrm{x}\ 3600 \quad \text{............ (7)}$$

Where, $\int \mathrm{I\,dV}$ – integral area, ΔV – potential window, v- scan rate, m – mass of active material, I – discharge current, and $\Delta\, t$ the discharging time.

Table S3. Comparison table with previously reported works

Electrode	Electrolyte	Working window	Capacitance	Reference no.
AC	PVdF-HFP/[PMpyr][NTf_2]	0-2V	93.72 Fg^{-1}	[1]
AC	PVdF/PVAc/$BMIMBF_4$	0-3V	93.3 Fg^{-1}	[2]
Porous carbon	PVP/PVdF-HFP/$Mg(CF_3SO_3)_2$/[bdmim][BF_4]	0-0.8V	133 Fg^{-1}	[3]
Graphene	Polyacrylonitrile (PAN)/[BMIM][TFSI]	0-3V	108 Fg^{-1}	[4]
f-MWCNTs	PVdF-HFP/EMImFAP/LiPF6	4V(-2 to 2V)	127 Fg^{-1}	[5]
Porous carbon	PVdF-HFP/MgTr	0-1V	150 Fg^{-1}	[6]
Graphite-PTFE **(present work)**	PVDF-HFP/1but3pent$BTBF_4$	0-6V	8.85 Fg^{-1}	

References

[1] R. Muchakayala, S. Song, J. Wang, Y. Fan, M. Bengeppagari, J. Chen, M. Tan, Development and supercapacitor application of ionic liquid-incorporated gel polymer electrolyte films, J. Ind. Eng. Chem. 59 (2018) 79–89. https://doi.org/10.1016/j.jiec.2017.10.009.

[2] L. Yang, J. Hu, G. Lei, H. Liu, Ionic liquid-gelled polyvinylidene fluoride/polyvinyl acetate polymer electrolyte for solid supercapacitor, Chem. Eng. J. 258 (2014) 320–326. https://doi.org/10.1016/j.cej.2014.05.149.

[3] S.N. Syahidah, S.R. Majid, Super-capacitive electro-chemical performance of polymer blend gel polymer electrolyte (GPE) in carbon-based electrical double-layer capacitors, Electrochim. Acta.

112 (2013) 678–685. https://doi.org/10.1016/j.electacta.2013.09.008.

[4] G.P. Pandey, T. Liu, C. Hancock, Y. Li, X.S. Sun, J. Li, Thermostable gel polymer electrolyte based on succinonitrile and ionic liquid for high-performance solid-state supercapacitors, J. Power Sources. 328 (2016) 510–519. https://doi.org/10.1016/j.jpowsour.2016.08.032.

[5] G.P. Pandey, S.A. Hashmi, Solid-state supercapacitors with ionic liquid based gel polymer electrolyte: Effect of lithium salt addition, J. Power Sources. 243 (2013) 211–218. https://doi.org/10.1016/j.jpowsour.2013.05.183.

[6] S.N. Syahidah, S.R. Majid, Ionic liquid-based polymer gel electrolytes for symmetrical solid-state electrical double layer capacitor operated at different operating voltages, Electrochim. Acta. 175 (2015) 184–192. https://doi.org/10.1016/j.electacta.2015.02.215.
